# Regenerative therapeutics for chronic obstructive pulmonary disease

**DOI:** 10.1016/j.pharmr.2026.100124

**Published:** 2026-02-04

**Authors:** Luke van der Koog, Henry R.D. Showell, Dyan F. Nugraha, Mareike Lehmann, Thomas M. Conlon, Ali Önder Yildirim, Rocío Fuentes-Mateos, Hoeke Baarsma, John-Poul Ng-Blichfeldt, Barbro N. Melgert, Antonella F.M. Dost, Janette K. Burgess, Stacy L.S. Yam, Irene H. Heijink, Sidrah Ahmed, Margherita Paschini, Evalyne M. Jansen, Wouter L.J. Hinrichs, Jill R. Johnson, Xinhui Wu, Anika Nagelkerke, Henderik W. Frijlink, Carla F. Kim, Reinoud Gosens

**Affiliations:** 1 Department of Molecular Pharmacology, Groningen Research Institute of Pharmacy, University of Groningen, Groningen, The Netherlands; 2 Department of Pharmaceutical Technology and Biopharmacy, Groningen Research Institute of Pharmacy, University of Groningen, Groningen, The Netherlands; 3 MimeCure BV, Eelde, The Netherlands; 4 Groningen Research Institute for Asthma and COPD (GRIAC), University Medical Center Groningen, University of Groningen, Groningen, The Netherlands; 5 Department of Pharmacy, Faculty of Health, Sari Mulia University, Banjarmasin, Indonesia; 6 Institute for Lung Research, Philipps-University Marburg, German Center for Lung Research (DZL), Marburg, Germany; 7 Institute for Lung Health (ILH), German Center for Lung Research (DZL), Giessen, Germany; 8 Institute of Lung Health and Immunity (LHI), Helmholtz Munich, Comprehensive Pneumology Center (CPC-M), German Center for Lung Research (DZL), Munich, Germany; 9 Institute of Experimental Pneumology, Ludwig-Maximilian University of Munich, Munich, Germany; 10 Cambridge Stem Cell Institute, Jeffrey Cheah Biomedical Centre, Cambridge Biomedical Campus, Cambridge, United Kingdom; 11 Hubrecht Institute for Developmental Biology and Stem Cell Research-KNAW, University Medical Centre Utrecht, Utrecht, The Netherlands; 12 Department of Pathology and Medical Biology, University Medical Center Groningen, University of Groningen, Groningen, The Netherlands; 13 Stem Cell and Regenerative Biology Program, Divisions of Hematology/Oncology and Pulmonary Medicine, Department of Pediatrics, Boston Children’s Hospital, Boston, Massachusetts; 14 Department of Genetics, Harvard Medical School, Boston, Massachusetts; 15 School of Biosciences, College of Health and Life Sciences, Aston University, Birmingham, United Kingdom; 16 Department of Physiology and Pathophysiology, University of Manitoba, Winnipeg, Manitoba, Canada; 17 Department of Pharmaceutical Analysis, Groningen Research Institute of Pharmacy, University of Groningen, Groningen, The Netherlands

## Abstract

Chronic obstructive pulmonary disease (COPD) is one of the most common lung diseases worldwide, characterized by an accelerated loss of lung function. A key problem underlying COPD is increased tissue destruction in combination with defective lung tissue repair. As current therapies do not modify the progression of the disease, new therapies aimed at restoring lung tissue repair in COPD need to be developed. In an attempt to address this major unmet need, there has been a surge in both preclinical and clinical studies, aiming to identify key mechanisms underpinning defective lung repair and the ability to inhibit or even reverse this defect. This includes small molecules such as retinoids, as well as advanced therapy medicinal products such as cell therapies or therapies with cell-derived products such as extracellular vesicles, or secreted proteins. The results of these endeavors have been variable with failures as well as successful proof-of-concepts. In this review, we provide an overview of the current state of the field, including modes of action of the therapeutics that are or have been considered for lung regeneration, including a discussion on the reasons for failure where relevant. In addition, we discuss hurdles in the clinical development of regenerative therapeutics for COPD including clinical outcomes, route of administration and formulation as these are pivotal considerations moving forward.

**Significance Statement:**

Chronic obstructive pulmonary disease is characterized by progressive alveolar destruction and defective epithelial regeneration. Targetable mechanisms, including cellular senescence, altered mesenchymal-epithelial signaling, and chronic inflammation, impair progenitor function and niche integrity. Therapeutic strategies that restore epithelial repair, including small molecules, biologics, and cell-based approaches, represent a promising path toward disease modification and long-term lung function restoration.

## I Introduction

### A Chronic obstructive pulmonary disease

Chronic obstructive pulmonary disease (COPD) is a major global health challenge, affecting over 300 million people and is ranked as the fourth leading cause of death worldwide, according to the World Health Organization.^1^^,^^2^ The disease is characterized by a progressive and largely irreversible loss of lung function and airflow limitation, leading to symptoms such as chronic cough, dyspnea, excessive mucus production, and, in some cases, wheezing and chest tightness.^3^, ^4^, ^5^ These symptoms significantly impact the daily functioning and quality of life of patients with COPD. COPD primarily affects individuals over 60 years old and is mainly caused by chronic exposure to airborne toxic substances, including tobacco smoke, air pollution, and occupational exposures to dust or wood particles.^3^^,^^4^^,^^6^, ^7^, ^8^ Additional risk factors include lung infections, abnormal lung development, and genetic predisposition. Globally, the burden of COPD is expected to rise further owing to continued population aging and increasing exposure to risk factors such as air pollution and smoking in low- and middle-income countries.^1^^,^^9^

COPD is a heterogeneous disease with 2 primary pathological components: chronic bronchitis and emphysema. Chronic bronchitis is characterized by persistent airway inflammation, mucus hypersecretion, small airway wall fibrosis, and epithelial remodeling.^3^, ^4^, ^5^^,^^10^ Emphysema involves the destruction of alveolar structures, reducing the surface area available for gas exchange and leading to progressive respiratory impairment.^3^, ^4^, ^5^ In addition to these changes, small airway disease (SAD) has emerged as a key driver of early COPD and a major contributor to airflow limitation. SAD involves inflammation, fibrosis, and luminal narrowing of terminal bronchioles (<2 mm in diameter), leading to obstruction well before emphysema becomes radiographically apparent.^11^, ^12^, ^13^ By contrast, emphysema typically develops later and predominates in advanced stages, when alveolar destruction and impaired gas transfer become more pronounced.^12^^,^^13^ Chronic exposure to harmful substances induces persistent inflammation and oxidative stress, driving tissue destruction and abnormal tissue repair. These processes contribute to small airway obstruction, alveolar wall damage, and ultimately to progressive lung function decline.^3^^,^^4^^,^^6^, ^7^, ^8^ It is important to emphasize that lung function decline in COPD is multifactorial, and the result of both inflammation, bacterial and viral infections, SAD, emphysema development, and mucus obstruction. A critical and relatively recent insight into COPD progression is that lung function decline does not occur in a steady, linear manner but rather in episodic phases, partially due to exacerbations.^14^ Many patients with COPD experience these exacerbations, which are defined as an acute worsening of COPD symptoms beyond normal day-to-day variation that requires additional treatments.^15^^,^^16^ Recurrent bacterial and viral infections account for approximately 50% of the total accelerated lung function loss throughout the life of a patient with COPD.^16^, ^17^, ^18^ Furthermore, mucus plugging appears to play a crucial role as longitudinal analysis of chest computed tomography (CT) of patients with COPD indicated that those patients without notable mucus plugs on chest CT or those with resolvable mucus plugs have similar rates of lung function decline, whereas patients with persistent presence of mucus plugs have substantially accelerated decline of lung function.^19^ This implicates that targeting these pathological processes in COPD has the potential to slow down disease progression.

At a population level, the only proven interventions to slow lung function decline in patients with COPD are tobacco control measures and reductions in air pollution.^20^^,^^21^ For individual patients, pharmacological treatment primarily focuses on symptom relief, exacerbation prevention, and quality-of-life improvement, rather than modifying the underlying disease. Current pharmacological options include bronchodilators, inhaled corticosteroids, phosphodiesterase-4 (PDE4) inhibitors, systemic corticosteroids, biologics, and antibiotics.^21^, ^22^, ^23^ Bronchodilators are the cornerstone of COPD management, with short-acting and long-acting *β*-agonists or muscarinic antagonists used to relieve airway constriction and improve lung function. Long-acting bronchodilators are preferred for most patients because of their superior efficacy in symptom control and exacerbation reduction.^14^^,^^21^ Inhaled corticosteroids are added for selected patients, particularly those with elevated eosinophil counts, to further reduce exacerbation risks. During acute exacerbations, systemic corticosteroids, and antibiotics are commonly used to manage airway inflammation and secondary infections.^14^^,^^21^ In addition to pharmacological therapy, several nonpharmacological interventions play a crucial role in COPD management.^24^^,^^25^ Pulmonary rehabilitation, which includes exercise training, nutritional guidance, and patient education, has been shown to improve physical function and quality of life.^25^ Long-term oxygen therapy is indicated for patients with chronic hypoxemia, whereas noninvasive ventilation can be beneficial for individuals with respiratory failure.^25^ In advanced COPD, surgical interventions such as lung volume reduction surgery, endobronchial valves and coils, or lung transplantation may be considered, but these invasive treatments are only suitable for a highly select group of patients.^25^, ^26^, ^27^, ^28^, ^29^, ^30^

Despite the availability of both pharmacological and nonpharmacological treatments, no currently approved intervention can reverse established COPD or fundamentally slow disease progression.^31^, ^32^, ^33^ The underlying challenge lies in the chronic imbalance between tissue injury and insufficient repair, particularly in the alveolar compartment. This imbalance stems from dysregulated interactions between inflammation, progenitor cell dysfunction, and aberrant tissue remodeling. As a result, the long-term prognosis for patients with COPD remains poor. Alternatively, a pharmacological strategy that reactivates endogenous lung repair mechanisms could offer a scalable and noninvasive solution to modifying disease progression. Such a therapy could modify the disease trajectory and improve long-term outcomes for patients with COPD.^32^^,^^33^ Given the increasing burden of COPD and the limitations of current treatment approaches, the development of regenerative pharmacological therapies represents a crucial research priority.^33^^,^^34^

To advance development of a pharmacological strategy with the capacity to reactivate endogenous lung repair mechanisms it is first necessary to understand the central elements that have key roles in this process in health and disease. In the next section a summary of current knowledge is provided.

### B Alveolar epithelial repair

The long-standing belief that the adult human lung lacks regenerative capacity is challenged by emerging evidence, including case reports, showing lung regrowth after surgical resection.^35^^,^^36^ Although the lung exhibits a low level of structural turnover during homeostasis, it possesses significant repair capacity after injury.^37^ Although the cellular mechanisms of adult lung regeneration remain incompletely understood, studies in animal models and human lung tissue have identified multiple stem and progenitor cell populations capable of responding to injury and facilitating repair.^37^, ^38^, ^39^ In vivo lineage-tracing studies have demonstrated that mature lung epithelial cells act as regionally restricted progenitors, maintaining and repairing tissue after mild injury in animal models.^40^ In the proximal airways, basal cells within the pseudostratified epithelium act as multipotent progenitors that self-renew and give rise to both secretory and ciliated epithelial cells.^41^, ^42^, ^43^, ^44^ In the terminal bronchioles, respiratory airway secretory cells function as progenitors for alveolar type (AT)2 cells, which are essential for maintaining and regenerating the alveolar niche.^45^^,^^46^ In addition, bronchioalveolar stem cells are crucial for bronchioalveolar epithelial repair and are suggested to contribute to the regeneration of both proximal and alveolar epithelial cell types after injury.^46^^,^^47^ Within the alveoli, AT2 cells play a central role in repair, serving as progenitors capable of self-renewal and differentiation into AT1 cells, which are critical for gas exchange.^48^^,^^49^ After lung injury, apoptosis of AT1 cells triggers the activation and proliferation of AT2 cells, which then differentiate into new AT1 cells to restore alveolar integrity.^48^^,^^49^ Functional epithelial regeneration requires both the proliferation of progenitor cells to replace lost cells and their differentiation into mature cell types, including surfactant-producing AT2 cells and barrier-forming AT1 cells. Recent studies have identified distinct AT2 subpopulations with specialized roles in regeneration. One such subpopulation, Axin2^+^ alveolar epithelial progenitors, is quiescent during homeostasis but proliferates rapidly after injury.^48^^,^^50^ Another population is the distal lung progenitor (integrin *α*6/*β*4^+^, surfactant protein C^−^), which replenish the AT2 cell pool after lung injury in mice.^51^ Additionally, a quiescent, immature AT2 subpopulation marked by programmed death-ligand 1 expands after pneumonectomy in mice and has also been identified in humans.^52^ Moreover, emerging data indicate that Club cells, secretory cells in the bronchiolar airways, act as facultative progenitor cells during alveolar repair. In murine models and in vitro 3-D culture systems, club cells (Scgb1a1^+^) have been shown to proliferate and differentiate into AT2- and AT1-like cells, forming alveolar-like structures.^53^ Furthermore, lineage-tracing studies identified a subpopulation of H2-K1^high^ club-derived progenitors that mobilize after bleomycin injury and contribute directly to alveolar cell lineages.^54^ More recently, airway secretory-cell derived p63^+^ progenitors (within the club/secretory cell compartment) were shown to enter distal alveolar regions and aid repair in severe lung injury.^55^ These findings suggest that under significant injury, club cells may act as a facultative alveolar progenitor pool, potentially compensating when classical alveolar progenitors are compromised. Together, these findings reveal an unexpectedly dynamic and plastic epithelial progenitor population within the distal lung, capable of coordinating repair after injury.

In COPD, disruption in the natural processes of alveolar epithelial repair, particularly in emphysema, results in an imbalance between tissue injury and the reduced capacity of alveolar progenitor cells to support repair.^56^^,^^57^ It is believed that repetitive injury in COPD leads to the depletion of the stem and progenitor cell pool, thereby limiting the regenerative capacity of the remaining epithelial progenitor cells.^56^ Although progenitor cell populations may survive in the lungs of patients with COPD, they may develop abnormalities that compromise their function. For instance, telomere shortening has been observed in both smokers with and without COPD, in addition to increased AT2 cell senescence in individuals with emphysema.^58^, ^59^, ^60^ Furthermore, the cellular composition of the alveolar compartment is altered in emphysema, with an increased proportion of AT2 cells and a reduction in AT1 cells, suggesting that many AT2 cells fail to undergo proper differentiation. On one hand this shift includes a numerical decline in functional epithelial cells, whereas on the other hand there is an accumulation of aberrant or arrested AT2 cells, which further impair regeneration.^61^, ^62^, ^63^ This suggests that chronic insults may push progenitor cells toward senescence or exhaustion, undermining their ability to support repair. Furthermore, critical signaling pathways that govern epithelial progenitor activity are dysregulated in COPD. For example, WNT signaling, a key pathway regulating AT2 cell behavior, is reduced in AT2 cells derived from patients with COPD, indicating impaired regenerative signaling.^64^^,^^65^ These alterations may further limit progenitor cell function and contribute to the progressive failure of alveolar repair observed in the disease.

Although much of the current research focuses on alveolar repair, it is increasingly recognized that regeneration of the small airways is equally important for restoring lung function. Early COPD is dominated by SAD, characterized by airway wall fibrosis, luminal narrowing, and loss of terminal bronchioles.^11^^,^^12^ Regeneration in this compartment will require both resolution of peribronchiolar fibrosis and re-epithelialization of the distal conducting airways, processes that remain poorly understood but represent critical targets for future repair-focused therapies.^66^

In summary, the alveolar epithelium contains several progenitor populations with significant reparative potential, but in COPD, these progenitors are impeded by cumulative damage, cellular senescence, and disrupted signaling. Understanding and reversing these dysfunctions is essential to restoring alveolar structure and function in affected individuals.

### C The alveolar progenitor niche

**Fig. 1:**
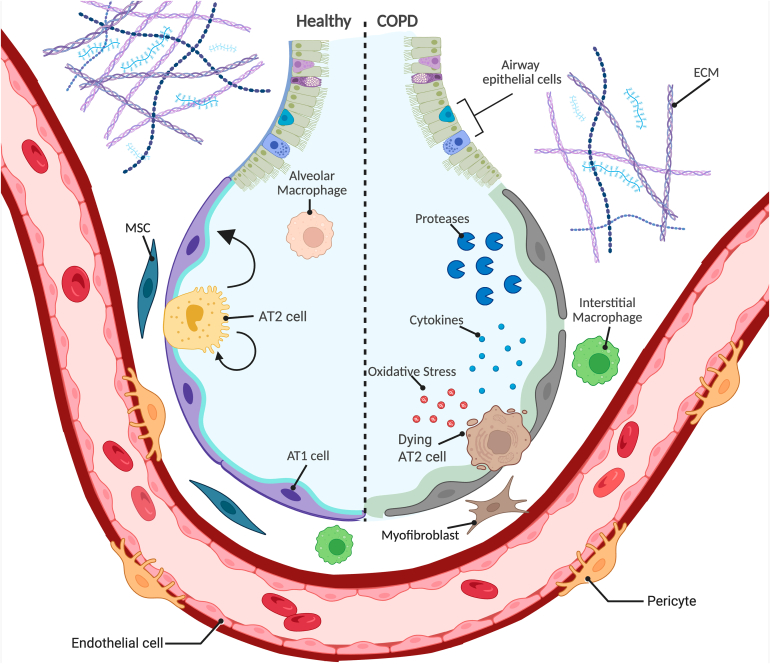
The healthy and disrupted alveolar niche in COPD. Alveolar epithelial progenitor cells do not function in isolation but reside within a specialized microenvironment, the alveolar progenitor niche, which orchestrates their behavior. This niche supports progenitor cell survival, regulates their proliferation and differentiation, and integrates repair signals after injury. In COPD, however, these niche interactions are disrupted, impairing repair capacity and contributing to progressive alveolar damage. SABAs, short-acting *β*2-adrenoceptor agonists; Created in BioRender (https://BioRender.com/cslgb1t) by Van der Koog, L.

Alveolar epithelial progenitor cells do not function in isolation but reside within a specialized microenvironment, the alveolar progenitor niche, which orchestrates their behavior. This niche supports progenitor cell survival, regulates their proliferation and differentiation, and integrates repair signals after injury.^38^^,^^67^^,^^68^ It is composed of various stromal cells and immune cells, microvascular endothelial cells (ECs), and the extracellular matrix (ECM), which together regulate progenitor cell behavior through complex cellular and molecular interactions.^67^^,^^68^ In healthy lungs, the niche provides the structural and biochemical cues necessary for epithelial regeneration and homeostasis. In COPD, however, these niche interactions are disrupted, impairing repair capacity and contributing to progressive alveolar damage.^57^^,^^69^^,^^70^ Dissecting the composition and function of this regenerative microenvironment, both in health and disease, offers critical insight for developing targeted therapies (Fig. 1). The following subsections explore the major components of the alveolar niche, including proinflammatory signals, macrophages, ECs, mesenchymal cells, and the ECM, and examine how each contributes to or hinders epithelial regeneration in COPD.

#### 1 Proinflammatory cytokines

COPD is characterized by persistent, sterile inflammation, with an increased presence of immune cells and elevated levels of proinflammatory cytokines, including interleukin (IL)-1, IL-6, IL-8, IL-17, tumor necrosis factor *α* (TNF-*α*), and type II interferon (IFN-*γ*).^71^, ^72^, ^73^, ^74^, ^75^, ^76^, ^77^ In addition, patients with COPD are more susceptible to infections, which can trigger inflammatory spikes that frequently lead to disease exacerbations.^15^^,^^78^ Although these cytokines are well known for their roles in modulating immune cell activity and sustaining inflammation, their direct effects on lung regeneration remain poorly understood. Elucidating how proinflammatory cytokines interfere with alveolar repair is critical for developing therapies that can effectively restore damaged alveolar tissue.

Several cytokines elevated in COPD, including IL-1*β*,^79^ IL-6,^67^^,^^80^ and TNF-*α*^79^ are typically viewed as inflammatory drivers, but emerging evidence suggests that their effects on epithelial regeneration are complex and context-dependent. In short-term assays such as organoid cultures, these cytokines promoted alveolar cell proliferation and survival.^67^^,^^79^ Moreover, transient IFN-*γ* exposure has been found to be necessary for effective epithelial repair after acute infection.^81^ Additionally, a recent study that modeled the complex inflammatory environment of a COPD exacerbation using a cytokine cocktail found increased epithelial proliferation under these conditions.^82^

In contrast, these same cytokines can also disrupt epithelial repair processes. TNF-*α* has been linked to alveolar dysfunction and impaired epithelial barrier integrity.^83^ IFN-*γ*, although beneficial in low doses, has been associated with emphysema development in overexpression models and can induce apoptosis in alveolar epithelial cells in both human and murine models.^84^, ^85^, ^86^, ^87^ Chronic IL-1*β* exposure has been shown to reprogram fibroblasts toward a proinflammatory state, diminishing their capacity to support epithelial growth in coculture organoid models.^88^ Additionally, IL-1*β* can drive AT2 cells to a transitional state between AT2 and AT1 identities, preventing full differentiation when exposure is prolonged.^89^ Notably, the previously mentioned cytokine exacerbation cocktail that enhanced proliferation also altered progenitor cell differentiation trajectories.^82^

Together, these findings underscore that the impact of COPD-associated cytokines on lung regeneration is complex and highly context-dependent. The same cytokine may exert either supportive or detrimental effects depending on its concentration, duration of exposure, and the affected cell type. This duality may help explain why COPD lungs often display disordered epithelial differentiation and accumulation of aberrant cell types.^45^^,^^90^ Consequently, therapies aimed solely at stimulating epithelial proliferation are unlikely to achieve functional repair fully. Instead, coordinated therapeutic strategies must aim to restore the critical balance between progenitor cell proliferation and differentiation within an appropriate alveolar niche.

#### 2 Macrophages

Although various immune cells are present in the alveolar progenitor niche, lung macrophages occupy a central and dual role, being capable of driving emphysema or promoting epithelial repair depending on their activation state. Historically, activated macrophages were described as polarized toward either proinflammatory (M1) or reparative (M2) phenotypes. However, several studies have demonstrated that alveolar macrophages from patients with COPD exhibit a mixed or aberrant activation profile, with features of both M1- and M2-like phenotypes rather than a simple polarization toward one subtype.^70^^,^^91^, ^92^, ^93^, ^94^, ^95^, ^96^ This complex activation state is often described as dysfunctional or reprogrammed, with impaired phagocytic and efferocytic capacity, altered protease–antiprotease balance, and ineffective inflammatory resolution.^97^ Consequently, the use of strict M1/M2 terminology is now discouraged in human studies, as macrophage activation represents a continuum rather than 2 discrete phenotypes.^98^

Lung macrophages are broadly categorized into alveolar and interstitial macrophages based on their anatomical location,^99^^,^^100^ with each population differing in origin, mode of replenishment, and contribution to inflammation and repair processes. Alveolar macrophages are derived from fetal liver progenitors and are long-lived, self-renewing cells that maintain surfactant balance and homeostasis in the lung under steady-state conditions.^101^^,^^102^ Upon injury, they are often depleted and can be replaced through local proliferation or by recruited alveolar macrophages originating from circulating monocytes.^103^ These monocyte-derived alveolar macrophages are highly plastic and can adopt either proinflammatory or reparative phenotypes.^104^ Although fetal-derived alveolar macrophages are linked to homeostatic and anti-inflammatory functions, monocyte-derived alveolar macrophages are thought to contribute most to COPD pathology and emphysema development.^105^^,^^106^ Interstitial macrophages, initially derived from yolk sac progenitors, are largely replaced postnatally by bone marrow-derived cells maintained by circulating monocytes.^107^, ^108^, ^109^ Located within the lung parenchyma or bronchial niches, interstitial macrophages contribute to immune regulation through antigen presentation and constitutively produce chemokines and immunosuppressive cytokines.^110^ Interstitial macrophages have been reported to be quantitatively and phenotypically altered in COPD and engage in immune-regulatory crosstalk with epithelial cells.^111^ Human and experimental data indicate that interstitial macrophages may protect against emphysema, positioning interstitial macrophages as modulators of chronic inflammation and tissue remodeling rather than passive bystanders,^112^ a perspective echoed by recent reviews calling for compartment-specific analysis of macrophage function in COPD.^113^

Although both alveolar macrophages and interstitial macrophages contribute to COPD pathology, most studies have focused on alveolar macrophages without distinguishing their developmental origin. Alveolar macrophages were found to contribute to alveolar destruction through the release of proteolytic enzymes and oxidative mediators.^114^, ^115^, ^116^ Although neutrophils also participate in this process, the number of alveolar macrophages correlates more strongly with emphysema severity.^117^ Moreover, animal studies demonstrated that emphysema development critically depends on macrophages and macrophage-derived matrix metalloproteinase 12 (MMP12), but not on neutrophils.^118^, ^119^, ^120^

More recent studies highlight that alveolar macrophages are also key regulators of lung repair after injury,^121^^,^^122^ a property that could be therapeutically exploited. A critical aspect of their function in the alveolar niche is crosstalk with alveolar epithelial progenitor cells.^123^ As mentioned before, macrophage function and phenotype are undeniably altered in COPD,^124^^,^^125^ but how this impacts repair is less understood. Aging and chronic exposure to noxious stimuli such as cigarette smoke can induce macrophage senescence, skewing their phenotype toward a proinflammatory state.^126^, ^127^, ^128^ In COPD, macrophages also exhibit impaired phagocytic and efferocytic function, leading to accumulation of apoptotic cells and persistent inflammation.^129^ This failure to clear debris not only delays resolution of injury but also alters epithelial-macrophage signaling, ultimately disrupting the fate and proliferation of alveolar type 2 progenitor cells. For instance, alveolar macrophage peroxisomes support AT2 self-renewal via lipid and mitochondrial regulation, and peroxisomal dysfunction through excessive inflammation was shown to result in progenitor cell dysfunction.^122^ Alveolar macrophages also secrete factors such as placenta-expressed transcript 1, which directly stimulate epithelial proliferation and barrier restoration.^121^ The importance of macrophage–epithelial interactions in lung regeneration was also highlighted by Rochford et al^130^, who showed that enhancing cyclic adenosine monophosphate (cAMP) signaling in proinflammatory monocyte-derived alveolar macrophages via PDE4b inhibition restored reparative capacity and resolved lung injury in mice. These findings suggest that therapeutic strategies aimed at reprogramming macrophages, such as through modulation of cAMP-PDE4b signaling or enhancement of peroxisomal function, could be used to restore proper epithelial–macrophages interactions and support alveolar repair in COPD.

#### 3 The microvascular endothelium

The alveolar capillary network is a vital component of the progenitor niche, supporting epithelial homeostasis and repair through both structural support and paracrine mechanisms. Alveoli are lined by a thin layer of pulmonary microvascular ECs, which form capillary networks tightly associated with the alveolar epithelium.^131^ These ECs are separated from the epithelium by a basement membrane, a specialized ECM layer that maintains the structural integrity of the blood-air barrier and enables efficient gas exchange.^131^ Although ECs are often considered passive conduits for oxygen and nutrient delivery, they also function as dynamic regulators of lung homeostasis and epithelial regeneration.^131^, ^132^, ^133^

ECs contribute to lung repair by interacting with alveolar epithelial progenitor cells to regulate their proliferation, self-renewal, and differentiation.^131^, ^132^, ^133^, ^134^, ^135^, ^136^ For instance, coculturing primary human AT2 cells with pulmonary ECs significantly enhanced alveolar organoid formation, demonstrating the role of endothelial-derived regenerative cues in epithelial repair.^137^ Additionally, studies have shown that intravenous administration of pulmonary ECs (CD45^−^VE-cadherin^+^CD31^+^) stimulated epithelial proliferation and differentiation and reversed elastase-induced emphysema in mice.^138^ A crucial mechanism through which ECs support lung repair is the secretion of angiocrine factors, which are signaling molecules that regulate epithelial progenitor activity.^131^, ^132^, ^133^ For example, in pneumonectomy models, ECs produced regenerative signals that drove epithelial progenitor expansion. One of these angiocrine factors, bone morphogenetic protein (BMP)4, and BMP6 have been identified as a key regulator of alveolar progenitor cell activity.^135^^,^^139^ Additionally, endothelial-derived hepatocyte growth factor (HGF) played a role in epithelial cell differentiation during lung development and repair, further reinforcing the importance of endothelial–epithelial crosstalk.^140^

In COPD, alveolar ECs become dysfunctional, which contributes to impaired epithelial repair and disease progression. Indeed, microvascular EC loss has been observed before alveolar destruction in patients with emphysema, suggesting that vascular dysfunction may be an early driver of disease rather than a secondary consequence.^137^^,^^141^ Furthermore, exposure of human pulmonary ECs to cigarette smoke (CS) reduced their ability to support alveolar organoid formation, indicating impaired regenerative signaling.^137^ This endothelial dysfunction likely deprives the progenitor niche of key angiocrine signals, compounding epithelial injury.

Endothelial injury contributes to COPD and emphysema; however, the roles of distinct EC populations remain unclear. Single-cell RNA sequencing has revealed significant EC heterogeneity in the lung, including macrovascular (maECs), microvascular (miECs), Car4-high ECs, and Atf3^+^ capillary ECs.^134^^,^^142^ After H1N1-induced injury, both Car4-high and other ECs proliferate, whereas Atf3^+^ ECs expand, supporting alveolar repair through genes regulating angiogenesis, migration, and development; endothelial-specific loss of Atf3 impairs regeneration, causing alveolar endothelial loss and emphysema-like changes. Car4 ECs form close contacts with AT1 cells across a thin, pericyte-free basement membrane and are lost after epithelial Vegfa deletion, leading to alveolar enlargement despite normal myofibroblasts.^143^ In patients with COPD, endothelial progenitor cells (EPCs, CD34^+^KDR^+^) are reduced and inversely correlated with emphysema, whereas circulating ECs remain largely unchanged but track with microvascular dysfunction.^144^ These findings highlight EC heterogeneity and indicate that impaired repair, rather than uniform loss, drives emphysema-related vascular pathology.

Although the role of ECs in lung regeneration is increasingly recognized, it remains incompletely understood. Further research is needed to explore whether endothelial-targeted therapies could enhance lung regeneration in COPD. Beyond restoring endothelial function, therapeutic strategies that harness angiocrine signaling pathways may offer novel opportunities to stimulate epithelial repair and improve clinical outcomes.

#### 4 Mesenchymal cells

Among the various components of the alveolar progenitor niche, lung-resident mesenchymal cells form a critical supportive element, particularly in regulating and fine-tuning epithelial development and repair.^145^ These cells, including various types of fibroblasts and lung mesenchymal stromal cells, are important for the production and maintenance of ECM that orchestrates tissue repair upon injury.^146^ They also secrete growth factors, inflammatory mediators, and extracellular vesicles (EVs), thereby providing paracrine cues to the surrounding endothelium and epithelium.^145^^,^^147^, ^148^, ^149^ Stromal fibroblasts are activated upon the release of transforming growth factor *β* (TGF-*β*) from injured epithelial cells and differentiate into ECM-producing myofibroblasts that proliferate and deposit ECM proteins.^148^ Meanwhile, fibroblasts also initiate paracrine signaling with AT2 cells and ECs through gaps in the basement membrane.^150^^,^^151^ The multidirectional interactions among fibroblasts, AT2 cells, and the endothelium guide immune cells from capillaries into interstitial space, and eventually across the alveolar epithelium to arrive in the alveolar airspace.^152^, ^153^, ^154^ In the small airways, similar fibroblast-driven fibrotic remodeling underlies airway wall thickening and luminal loss. Preclinical and pathological studies show that small-airway narrowing and loss precede emphysematous changes and correlate strongly with lung-function decline.^11^^,^^155^ Targeting myofibroblast activation, TGF-*β* signaling, or aberrant extracellular matrix crosslinking can partially reverse peribronchiolar fibrosis and reopen obstructed airways.^156^ Understanding how to re-establish a reparative rather than fibrotic fibroblast phenotype may be key to restoring small-airway patency in early COPD.

Fibroblasts derived from lung tissue from patients with COPD exhibit reduced proliferative capacity, diminished responsiveness to injury signals, increased senescence, and a profibrotic phenotype.^146^^,^^147^^,^^157^, ^158^, ^159^, ^160^, ^161^, ^162^ Evidence suggests that extensive exposure to CS may permanently alter the fibroblast responsiveness in COPD, where mesenchymal stem/stromal cells (MSCs) and fibroblasts exhibit functional deficiencies such as a reduction in growth factors (fibroblast growth factor [FGF]2; vascular endothelial growth factor [VEGF]; and HGF) secretion.^147^^,^^163^, ^164^, ^165^, ^166^ In some instances, COPD fibroblasts release more TGF-*β*1 but exhibit dysregulation of the TGF-*β*/Smad pathway and blunted transcriptional/ECM responses, leading to an impaired ability to produce ECM components.^161^^,^^167^ In addition to reduced secretion of key growth factors, COPD fibroblasts display an aberrant response to TGF-*β* stimulation, characterized by diminished proteoglycan production, impaired ability to support epithelial organoid formation in vitro, and a shift toward senescence or proinflammatory fibroblast phenotypes in response to matrix degradation.^167^, ^168^, ^169^ Consequently, the reduced growth factor secretion and altered growth factor signaling result in a dysregulated repair program characterized by excessive ECM deposition, impaired epithelial regeneration, and the development of emphysematous lesions.^145^^,^^158^

Senescence markers, such as laminin-B1, cyclin dependent kinase 1A (p21), cyclin dependent kinase 2A (p16), and senescence-associated *β*-galactosidase are elevated in fibroblasts from patients with COPD and this is associated with resistance to apoptosis and increased secretion of growth factors and proinflammatory cytokines. The latter are part of the senescence-associated secretory phenotype that amplifies inflammation and tissue remodeling.^158^^,^^159^ Crosstalk between fibroblasts and epithelial cells is important for maintaining homeostasis in lung tissue.^170^ In COPD, dysfunctional fibroblasts may propel epithelial-to-mesenchymal differentiation, with subsequent migration through a fragmented reticular basement membrane.^171^ This leads to a loss of epithelial–mesenchymal contact and impaired leukocyte clearance, contributing to leukocyte accumulation in the interstitial space and further disrupting alveolar repair.^152^ Together, these findings highlight mesenchymal dysfunction as a central contributor to impaired progenitor activity and regenerative failure in COPD.

#### 5 The extracellular matrix

In addition to cellular components, the ECM forms an integral part of the alveolar progenitor niche. The ECM is a network of proteins and other supporting molecules that provide structural and biochemical support to the surrounding cells, which dictates the tissue integrity and lung function.^172^^,^^173^ A crucial role of ECM is providing a scaffold that supports the lung architecture. Beyond its structural architecture, the ECM also serves as a dynamic growth factor reservoir and signaling interface that modulates immune cell migration, activation, and retention within lung tissue.^174^^,^^175^ Altered matrix degradation and composition in COPD ECM expose cryptic fragments and modify chemokine gradients, thereby dysregulating leukocyte trafficking and contributing to the perpetuation of chronic inflammation.^176^^,^^177^ This is primarily attributed to ECM proteins such as collagens, elastin, and proteoglycans (decorin, perlecan, biglycan, and veriscan), which together provide tensile strength, elasticity, and facilitate fiber assembly and signaling, respectively.^173^^,^^178^, ^179^, ^180^, ^181^ The alveolar structural and functional integrity relies on appropriate arrangement of these ECM proteins.^181^, ^182^, ^183^ In COPD, extensive ECM remodeling contributes to small airway fibrosis and narrowing. Although this review focuses on alveolar repair, resolving aberrant ECM deposition and restoring elastic recoil in the small airways will be equally important for functional regeneration of the distal lung.^180^^,^^184^ In emphysema, alveolar destruction results from progressive damage to the ECM network in the lung parenchyma.^185^ The loss of collagen type I and elastin correlate with a reduction of tissue stiffness, which makes the structure more susceptible to external forces applied during normal expiration that result in alveolar overexpansion, wall rupture, less oxygen exchange and collapse.^178^^,^^185^^,^^186^

Numerous ECM alterations are observed in lung tissue from patients with COPD, including increased ECM degradation, especially elastin and collagen I, along with dysregulated ECM turnover and abnormal remodeling.^71^^,^^187^ There are variable reports of ECM changes in COPD, possibly reflecting different tissue sampling and staining protocols. A recent study described altered signatures of ECM expression profiles in COPD parenchyma, including lumican and collagen type 6*α*1, with these changes correlating with disease severity.^184^ In parallel, decreased elastin levels in COPD ECM may result from CS-linked elastase/antielastase imbalance. This imbalance leads to the formation of dysfunctional elastic fibers, which greatly reduce elasticity of the lung tissue.^188^ Moreover, decreased decorin levels in the parenchyma disrupt the regulation of collagen fibrillogenesis and inhibit cellular responses to inflammatory cytokines.^146^^,^^167^^,^^189^^,^^190^ Elevated levels of versican in the parenchyma may further inhibit the assembly of elastic fibers and contribute to impaired matrix organization.^147^ In contrast, Annoni et al^178^ reported a proportional reduction in elastin and versican in the distal parenchyma of patients with COPD, highlighting the heterogeneity of ECM alterations across disease stages and lung regions. Taken together, these findings highlight that ECM remodeling in COPD not only reflects structural disintegration but also contributes to progenitor cell dysfunction and impaired alveolar repair.

### D Summary and outlook

In summary, COPD is characterized by progressive alveolar destruction and impaired tissue repair. Although the alveolar epithelium contains progenitor populations with regenerative capacity, this potential is disrupted in COPD due to chronic inflammation, cellular senescence, and dysfunctional niche signaling. The alveolar progenitor niche, which includes immune cells, ECs, fibroblasts, and the ECM, plays a central role in coordinating epithelial repair (Fig. 1). In COPD, alterations across all these components converge to create a nonpermissive (ie, inhibitory) environment for regeneration. A better understanding of these interactions will enable the development of regenerative therapies aimed at restoring alveolar structure and improving long-term outcomes for patients with COPD. Given the multifactorial nature of repair failure in COPD, a wide range of therapeutic strategies are currently being explored. These include small molecules that directly stimulate epithelial regeneration, compounds that inhibit processes contributing to regenerative dysfunction, cell-based therapies, and emerging approaches involving EVs or cell-derived proteins. The following sections will discuss these pharmacological and biological strategies in detail, highlighting their mechanisms of action, regenerative potential, and current stage of development.

## II Regenerative therapeutics for chronic obstructive pulmonary disease

### A Small molecules that directly activate regeneration

#### 1 Cyclic adenosine monophosphate-based drugs

cAMP is a key intracellular second messenger involved in various physiological processes, including lung homeostasis, inflammation, and metabolic regulation. Given its anti-inflammatory, bronchodilatory, and potential proregenerative properties, pharmacological strategies aimed at increasing intracellular cAMP levels have emerged as promising therapeutic avenues for COPD. In this context, PDE4 inhibitors and other cAMP-modulating compounds have been extensively investigated in both preclinical and clinical settings.^191^

**Fig. 2:**
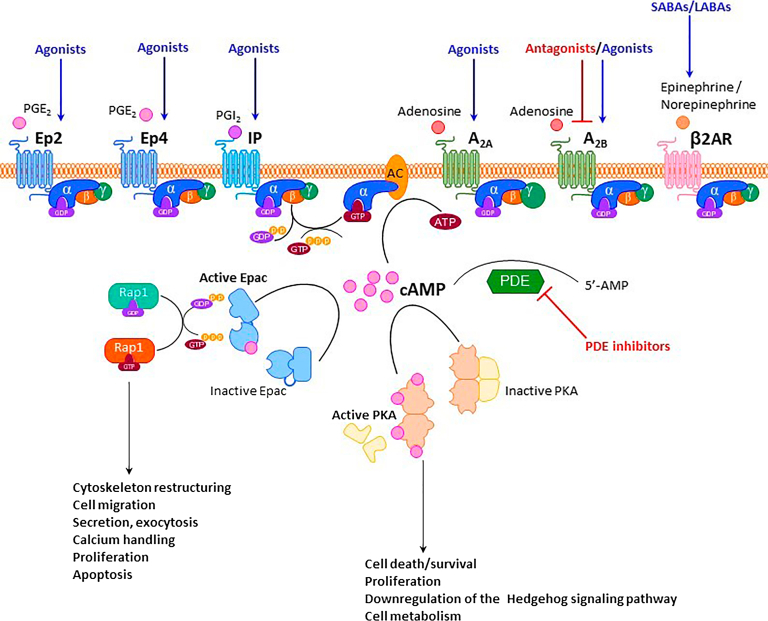
Activation of cAMP signaling by G protein-coupled receptors including EP_2_, EP_4_ and IP, prostanoid, A_2A_, A_2B_ adenosine, and *β*_2_ adrenoceptors (*β*_2_AR). Downstream effects have been linked to regeneration.

cAMP can be activated by a wide range of extracellular and intracellular stimuli, triggering downstream signaling effectors such as protein kinase A and exchange protein directly activated by cAMP (Epac). Through the activation of these key effectors, cAMP exerts regulatory effects on inflammation and energy metabolism (Fig. 2).^191^

##### a β-Adrenoceptor agonists

*β*2-Adrenoceptors are highly expressed in the alveolar walls, endothelium, pulmonary arteries, tracheal smooth muscle, and bronchial epithelium, where they contribute to airway tone and fluid clearance.^192^ Activation of the *β*2-adrenoceptor stimulates adenylyl cyclase via G proteins, thereby increasing intracellular cAMP levels.^191^ Consequently, *β*2-adrenoceptor agonists have become essential components of pharmacological therapy for asthma and COPD.^192^ These agents induce bronchodilation via cAMP-mediated activation of protein kinase A,^193^ while also promoting mucociliary clearance and attenuating inflammation.^194^

Short-acting *β*2-adrenoceptor agonists, such as salbutamol, pirbuterol, and terbutaline, provide rapid symptom relief, whereas long-acting *β*2-adrenoceptor agonists (LABAs), including formoterol, salmeterol,^192^ indacaterol,^195^ olodaterol,^196^ and vilanterol,^197^ offer sustained bronchodilation, although formoterol is now also used as a reliever medication based on its fast-acting properties. LABAs are commonly used in combination with long-acting anticholinergics based on the observation that dual bronchodilation is more effective than single bronchodilation and more effective than the combination of LABAs with inhaled corticosteroids.^198^^,^^199^ Beyond their bronchodilatory effects, LABAs have been shown to reduce airway smooth muscle proliferation, enhance ciliary function, and decrease the release of inflammatory mediators and neutrophil activation.^200^

Regarding regenerative potential, *β*2-adrenoceptor agonists have demonstrated beneficial effects on epithelial repair in models of acute respiratory distress syndrome^201^ and bovine bronchial epithelial cell wound healing.^202^ The potential role of *β*2-adrenoceptor agonists on inflammation and epithelial repair in acute respiratory distress syndrome has been reviewed in detail by Sriram et al.^203^ However, recent findings suggest that *β*2-adrenoceptor agonists may impair airway epithelial regeneration via cAMP-independent mechanisms, specifically through enhanced activity of protein phosphatase 2A.^204^ Indeed, epithelial *β*1 integrins play a role in alveolar homeostasis and restitution through the regulation of alveolar epithelial cell inflammation.^205^ Similar inhibitory effects have been observed on wound healing in keratinocytes^206^^,^^207^ and corneal epithelial cells.^208^ These findings raise concerns regarding the long-term impact of *β*2-adrenoceptor agonists on epithelial repair in chronic respiratory diseases, where delayed wound healing could increase susceptibility to infections and disease progression. With respect to mucus plugging, which is known to accelerate lung function decline,^19^ *β*2-adrenoceptor agonists may have beneficial effects as they increase ciliary beat frequency, enhancing mucociliary transport.^209^^,^^210^ However, this is less effective if mucus is very thick or infection/inflammation dominates and consequently the impact of LABAs on lung function decline may be present, but is not overwhelming.^211^

##### b Phosphodiesterase inhibitors

Intracellular cAMP levels are tightly regulated by PDE activity. Under physiological conditions, cAMP suppresses proinflammatory responses. However, in COPD, increased PDE activity leads to excessive hydrolysis of cAMP into its inactive form, 5’AMP, resulting in diminished cAMP signaling and exacerbated inflammation.^191^

There are 11 known PDE families in mammals, comprising over 50 isoforms, some of which exhibit tissue-specific expression patterns. Among them, PDE3, PDE4, and PDE7 are particularly enriched in the lungs.^212^^,^^213^ PDE4, the predominant isoform responsible for cAMP degradation in pulmonary tissues, has garnered significant interest as a therapeutic target in COPD because of its upregulation, particularly in macrophages from patients with COPD.^214^

PDE4 inhibitors prevent cAMP breakdown, thereby enhancing its signaling effects. Roflumilast, a selective PDE4 inhibitor approved for use in COPD, has demonstrated efficacy in reducing moderate and severe exacerbations by 12% and 16%, respectively.^215^^,^^216^ However, its widespread clinical use has been limited by systemic side effects such as nausea, diarrhea, weight loss, and abdominal discomfort.^217^

Ensifentrine is a novel dual PDE3/PDE4 inhibitor with the PDE3 inhibition responsible for bronchodilation being the most potent activity, which was recently approved for the treatment of COPD. It enhances lung function, quality of life and reduces the rate of exacerbations in patients with COPD, which is relevant to COPD progression.^218^ The impact of ensifentrine on lung regeneration is not clear, but it does inhibit injury of human microvascular ECs and alveolar epithelial cells in response to methicillin-resistant *Staphylococcus aureus* in vitro.^219^ It will be of interest to explore the impact of ensifentrine on lung regeneration further, because of its dual action, which broadens its mode of action, affecting both structural cells such as fibroblasts and epithelial cells as well as inflammatory cells.^220^

Apremilast, another oral PDE4 inhibitor approved for inflammatory conditions such as psoriasis and psoriatic arthritis, also holds potential for COPD management, especially in patients prone to recurrent lung infections because of its anti-infective and anti-inflammatory properties. Efforts to formulate apremilast for inhaled administration via aerosolized nebulization are currently underway.^221^ In a rat model, apremilast has shown the capacity to reduce lung inflammation and promote airway repair upon lipopolysaccharide (LPS) stimulus.^222^

Tanimilast (CHF6001) is a next-generation inhaled PDE4 inhibitor developed to enhance therapeutic efficacy within the lungs while reducing systemic exposure and associated side effects.^223^ Unlike selective inhibitors, Tanimilast targets all 4 PDE4 isoforms (A–D) without isoform preference. It has shown broad anti-inflammatory activity across various human immune and structural cell types, including neutrophils, eosinophils, macrophages, dendritic cells, lymphocytes, and bronchial epithelial cells. These effects have also been demonstrated in human lung explants^224^ and precision-cut lung slices.^225^ By elevating intracellular cAMP levels, Tanimilast suppresses the production and release of a wide array of inflammatory mediators, while also reducing chemotactic responses and reactive oxygen species production.^223^^,^^226^ In preclinical models of acute and subchronic pulmonary inflammation, Tanimilast has been effective in reducing neutrophil recruitment and overall inflammatory burden.^227^, ^228^, ^229^ It is currently under evaluation in 2 phase III clinical trials (NCT04636801 and NCT04636814) as an add-on therapy to inhaled corticosteroids, LABAs, and long-acting muscarinic antagonists in patients with COPD and chronic bronchitis who continue to experience symptoms despite triple therapy.

##### c Prostanoids

Prostanoids are a family of lipid mediators derived from the arachidonic acid cascade, including prostaglandin prostaglandin D_2_ (PGD_2_), PGE_2_, PGF_2*α*_, PGI_2_, and thromboxane A2. Among these, PGE_2_ and PGI_2_ have garnered particular interest in chronic respiratory diseases such as asthma, COPD, and idiopathic pulmonary fibrosis.^230^ PGE_2_ is ubiquitously produced by various lung cell types, with epithelial cells and macrophages being its principal sources,^230^^,^^231^ whereas PGI_2_ is predominantly produced by ECs.^230^

Prostanoids exert both autocrine and paracrine effects through binding to G protein-coupled receptors. PGE_2_ interacts with 4 distinct E prostanoid (EP) receptors (EP1–EP4), whereas PGI_2_ signals via the PGI_2_ recetor (IP) receptor. The downstream signaling outcome depends on the specific receptor subtype engaged. EP1 activates phospholipase C, leading to protein kinase C activation and an increase in cytosolic calcium, whereas EP3 inhibits adenylyl cyclase, thereby reducing cAMP levels. In contrast, EP2, EP4, and IP receptors stimulate adenylyl cyclase and increase intracellular cAMP. As such, the net effect of PGE_2_ signaling is determined by the expression pattern of its receptors on the target cell.^230^

The role of PGE_2_ and PGI_2_ signaling in lung repair in COPD remains controversial. Elevated PGE_2_ levels and increased expression of EP2/EP4 receptors and PGI_2_ have been detected in fibroblasts^161^^,^^232^ and airway secretions^233^^,^^234^ from patients with COPD. This has led to the hypothesis that enhanced prostanoid signaling may contribute to defective repair mechanisms and emphysema progression. However, several preclinical studies challenge this notion. Administration of a PGI_2_ analog conferred significant protection against CS extract-induced emphysema in rats,^235^ whereas treatment with stable analogs of PGE_2_ (16,16-dimethyl prostaglandin) and iloprost (a PGI_2_ analog) promoted epithelial regeneration and alveolar differentiation in lung organoid models exposed to CS extract.^236^ Furthermore, selective EP2 and EP4 receptor agonists have shown potential in inhibiting fibroblast-to-myofibroblast differentiation following TGF-*β* stimulation,^237^ and even in reversing it,^238^ suggesting a role in mitigating fibrosis and preventing mesenchymal exhaustion.

##### d Adenosine

Adenosine is a purinergic signaling molecule that accumulates extracellularly in response to tissue stress or injury. Elevated levels of adenosine have been reported in both healthy smokers and patients with COPD, showing a negative correlation with forced expiratory volume in 1 second (FEV_1_%) and increasing as disease severity progresses.^239^ These changes include the upregulation of CD73, which converts 5′-AMP into adenosine, and a downregulation of adenosine deaminase activity leading to reduced breakdown.^240^ Adenosine signaling can elicit proinflammatory or anti-inflammatory responses, as well as tissue-destructive or regenerative effects, depending on the receptor subtype and cellular context, which complicates its therapeutic targeting.

Adenosine exerts its effects via 4 G protein-coupled receptor subtypes: A_1_, A_2A_, A_2B_, and A_3_ receptors. Although A_1_ and A_3_ receptors inhibit adenylyl cyclase and reduce intracellular cAMP levels, A_2A_ and A_2B_ receptors activate adenylyl cyclase, leading to increased cAMP production.^241^ Interestingly, the therapeutic strategies differ by receptor: antagonists of A_1_, A_2B_, and A_3_ receptors, and agonists of A_2A_ receptors, have shown potential benefit in the treatment of asthma and COPD.^241^ Specifically, A_2A_ receptor agonism has demonstrated anti-inflammatory effects across various animal models of airway disease.^242^, ^243^, ^244^ In humans, A_2A_ receptors are expressed in bronchial and alveolar epithelial cells, as well as in smooth muscle and ECs.^245^ Despite encouraging preclinical data, clinical transition has been proven challenging. For example, the selective A_2A_ agonist UK432,097 was discontinued after failing to demonstrate efficacy in a phase II trial in COPD (NCT00430300).

Conversely, A_2B_ is the adenosine receptor with the lowest affinity but is highly inducible under inflammatory conditions. Its activation has been associated with airway inflammation and tissue remodeling,^246^^,^^247^ playing a key role in fibrosis development.^248^ Inhibiting A_2B_ with the selective antagonist CVT-6883 has been shown to reduce these effects in murine models^249^; moreover, blocking A_2B_ attenuated pulmonary hypertension in a murine model of emphysema and vascular remodeling.^250^ Although adenosine signaling is closely linked to COPD pathogenesis and disease progression, the complex interplay between receptor subtype distribution, affinity, and downstream effects presents significant challenges in translating this knowledge into effective, targeted therapies.

In summary, cAMP-elevating agents hold significant promise for improving lung regeneration in COPD through their anti-inflammatory, bronchodilatory, and potentially proreparative effects. Collectively, these findings highlight the therapeutic potential and current limitations of cAMP-based interventions, emphasizing the need for more targeted and cell-specific approaches to enhance lung regeneration in COPD, especially in patients with advanced epithelial injury (Fig. 2).

#### 2 Glucocorticosteroids

Glucocorticosteroids represent one of the most common classes of drugs prescribed for chronic inflammatory respiratory illnesses, although the perspective on their clinical use in COPD has changed over the past decades. Initially one of the mainstay drugs for COPD management, it is now increasingly clear that corticosteroids do not sufficiently counteract inflammation in all patients with COPD, whereas they do increase the risk of side effects such as pneumonia.^251^, ^252^, ^253^ Meta-analyses indicate that dual bronchodilation with a long-acting anticholinergic and a LABA is superior in terms of FEV_1_ outcomes and prevention of exacerbations in comparison with the combination of a corticosteroid and a LABA.^198^ On the other hand, the risk of hospitalization due to pneumonia increases with dose and duration of corticosteroid treatment in patients with COPD.^251^, ^252^, ^253^ A notable exception are patients with eosinophilic inflammation in whom postbronchodilator FEV_1_ improves with corticosteroid use and more so than in patients without eosinophilic inflammation, but this is not typical of emphysema.^254^ Accordingly, corticosteroid use is no longer the recommended initial treatment for patients with stable COPD, and is only considered for those with a high exacerbation risk if blood eosinophil numbers exceed 300/*μ*L.^255^

Corticosteroids are mostly used as inhaled corticosteroids, though systemic treatment with corticosteroids is also used in some patients, preferably for shorter time windows during acute exacerbation management to avoid side effects.^255^ Clinically used inhaled corticosteroids include budesonide, fluticasone, ciclesonide, and beclomethasone, whereas beclomethasone, dexamethasone, prednisone, prednisolone, methylprednisolone, hydrocortisone, and triamcinolone may all be used as systemic treatments for the management of COPD exacerbations.^256^ The mode of action of glucocorticosteroids involves binding to the glucocorticoid receptor (GR), which in its inactive state is cytosolic and bound to heat shock protein 90. Dimerization of ligand-bound GR and nuclear translocation allows for the binding to glucocorticosteroid responsive elements within the genome, resulting in the activation of anti-inflammatory genes such as lipocortin-1 and the repression of proinflammatory genes such as genes encoding for cytokines and cyclooxygenase-2. In addition, monomeric ligand-bound GR can bind to transcriptional regulators involved in proinflammatory gene expression such as AP-1 and nuclear factor-*κ*B (NF-*κ*B).^257^ Moreover, nongenomic effects of glucocorticosteroids have been reported, including smooth muscle relaxation and immunosuppression, which are possibly dependent on a membrane bound GR.^258^^,^^259^

Glucocorticosteroids do not appear to have major beneficial direct effects on epithelial regeneration. In an elastase rabbit model of emphysema, intratracheal instillation of porcine pancreatic elastase-induced changes in lung function and in airspace size, but these changes were not counteracted by dexamethasone.^260^ Similarly, in a mouse model of CS-exposure, treatment with budesonide failed to improve the regenerative capacity of AT2 cells.^236^ In fact, in vitro exposure to budesonide even reduced the formation of alveolar epithelial organoids.^236^ Studies using human airway epithelial cells report similar findings and show that the corticosteroid dexamethasone increases apoptosis and slows down wound closure.^261^ Possibly, this effect is related to the activation of differentiation programs by corticosteroids, limiting the progenitor capacity of epithelial cells, particularly when corticosteroids are applied before the injury.^261^^,^^262^ Glucocorticosteroids also reduce the expression of HGF in fibroblast cultures, providing an additional explanation for the negative effects on progenitor function.^263^

Despite these limited direct effects on progenitor cell function, glucocorticosteroids may contribute beneficially by inhibiting the inflamed lung microenvironment. Although indirect, and of limited effect size, beneficial effects of glucocorticosteroids on the progression of lung function decline, and on the progression of emphysema development assessed by CT imaging, have been reported.^264^, ^265^, ^266^ In addition, in at least a proportion of patients with COPD characterized by eosinophilia, glucocorticosteroids help to reduce the risk of exacerbations, disease events known to contribute to accelerated lung function decline.^267^^,^^268^ These indirect beneficial effects may be explained by changes in eosinophilic airway inflammation, by changes in matrix composition in the airways, or by changes in airway epithelium gene expression associated with cell cycle and oxidative phosphorylation.^269^^,^^270^

Moreover, corticosteroids inhibit mucus production by airway epithelial cells,^271^ which may have both direct and indirect effects on lung function decline. In summary, beneficial effects of glucocorticosteroids on disease progression may exist in at least a subgroup of patients with COPD, characterized by eosinophilic inflammation. These effects are unlikely to be the result of any direct beneficial effects of glucocorticosteroids on alveolar or airway repair, but instead related to suppression of inflammation in susceptible individuals. These findings highlight the unmet need for regenerative therapeutics in COPD, particularly for individuals with emphysema.

#### 3 Mucolytic agents

Mucus plugging appears to play a crucial role in COPD as those patients without notable mucus plugs or those with resolvable mucus plugs have similar rates of lung function decline, whereas patients with persistent presence of mucus plugs have substantially accelerated decline of lung function.^19^ Not only the presence but also the composition of mucus is altered in COPD. The presence of Mucin (MUC) 5B is higher in COPD and so is the expression of the insoluble MUC2. Moreover, goblet cell metaplasia and an increased ratio of mucus cells to serous cells in the submucosal glands contributes to COPD.^272^ Accordingly, mucolytic drugs are used in the treatment of COPD, which include N-acetylcysteine, carbocysteine, erdosteine, l-methylcysteine, and fudosteine, all of which break up the cysteine bridges present in mucin proteins leading to less viscous mucus, and bromhexine, which targets glycosylation of mucin proteins, leading to less viscous mucus as well. Moreover, ambroxol is sometime used, which is an expectorant drug that drives fluid secretion.^273^ The available clinical data indicate that mucolytics significantly reduce the rates of exacerbation, shortened the duration of antibiotic use and exacerbations, prolonged the time to first exacerbation, and had a tendency to reduce the occurrence of ≥2 exacerbations in patients with stable COPD compared with placebo.^273^ Mucolytics did not improve lung function, mortality, and quality of life. There is no direct evidence that mucolytics can be regenerative, but indirect effects driven by the beneficial effects on exacerbation management may lead to reductions in lung tissue injury.

#### 4 Peroxisome proliferator-activated receptor γ ligands

Peroxisome proliferator-activated receptor *γ* (PPAR*γ*) is, similar to the glucocorticosteroid receptor, a nuclear receptor that plays a critical role in regulating diverse cellular responses including glucose metabolism, lipid homeostasis, and adipocyte differentiation.^274^ Unlike the glucocorticosteroid receptor, which resides in the cytoplasm when inactive and requires ligand-induced nuclear translocation, PPAR*γ* is constitutively located in the nucleus and is activated through ligand-induced conformational changes. PPAR*γ* is activated by endogenous ligands such as fatty acids and eicosanoids (such as 15-deoxy-*Δ*12,14-prostaglandin J2 [15d-PGJ2]) as well as synthetic ligands such as thiazolidinediones.^274^ Upon ligand binding, PPAR*γ* undergoes a conformational change that facilitates its heterodimerization with the retinoid X receptor (RXR). This PPAR*γ*-RXR complex then binds to specific DNA sequences known as peroxisome proliferator response elements located in the promoter regions of target genes.^275^ The activation of PPAR*γ* leads to the recruitment of coactivators such as PGC-1*α* and the displacement of corepressors, ultimately resulting in the transcriptional regulation of genes.^275^

15d-PGJ2 exerts anti-inflammatory effects by suppressing proinflammatory cytokines in part via PPAR*γ* signaling and by inhibiting NF-*κ*B signaling.^276^ In addition, 15d-PGJ2 activates Nrf2 signaling to balance oxidant defense mechanisms.^277^ Thiazolidinediones including rosiglitazone and pioglitazone represent selective and more stable PPAR*γ* ligands, used for the management of type 2 diabetes, as they restore insulin sensitivity in peripheral organs such as the liver and fat tissues.^256^ But PPAR*γ* receptors are far from specific to fat and liver tissues, and are also widely expressed in structural and circulating cells present in the lung. Retrospective analysis of pioglitazone use in patients with COPD and type 2 diabetes hints to potential protective effects on COPD, but these would need to be confirmed in prospective studies.^278^ Similar protective effects of thiazolidinedione use in patients with COPD has been associated with reductions in exacerbation risk.^279^

Nonetheless, in vitro and in vivo evidence does support a beneficial role for thiazolidinediones in COPD. PPAR*γ* ligands reduce the production of proinflammatory cytokines in alveolar macrophages obtained from patients with COPD, and enhanced gene expression associated with the alternative activation pathway.^280^ PPAR*γ* ligands also enhance efferocytosis and inhibit NF-*κ*B signaling.^281^ In line with a protective, anti-inflammatory function, the expression of PPAR*γ* and of PGC-1*α* progressively decreases in the lungs of patients with moderate and severe COPD.^282^ The expression of 15d-PGJ2 is also reduced in COPD, whereas that of the oxidative stress indicators HO-1 and NOX4 is increased.^283^ Furthermore, PPAR*γ* supports the expression of GPx3, which protects against oxidative stress in COPD.^284^ Accordingly, treatment with thiazolidinediones has beneficial effects on the development of airway remodeling and emphysema development in mice and rats exposed to CS.^285^^,^^286^ These effects may in part be indirect, by reducing lung damage, as PPAR*γ* ligands have anti-inflammatory effects on macrophages and restore the protease/antiprotease balance.^285^, ^286^, ^287^, ^288^ On the other hand, the experimental PPAR*γ* ligand LJ-529, which also acts as an adenosine A_3_ receptor agonist, prevented emphysema development in a mouse model of elastase-induced lung injury, suggestive of direct beneficial effects on epithelial repair as well.^289^ A recent publication supports this contention and shows that rosiglitazone promotes lung organoid growth of both control and idiopathic pulmonary fibrosis-derived epithelial cells, whereas the PPAR*γ* inverse agonist GW9662 reduces lung organoid growth.^290^ The relevance of this effect for COPD remains to be established, but does warrant further investigation in view of the large number of patients with COPD who also have type 2 diabetes, in whom leveraging such a dual beneficial role for pioglitazone would be an attractive therapeutic strategy.

#### 5 Retinoids

Retinoic acid (RA) signaling has potential to modulate population health at scale in part because active ligands are derived from dietary vitamin A, obtained from meat and plants as retinyl esters and carotenoids. Ingested retinoids are distributed to target tissues either postprandially in chylomicrons, or via hydrolysis into retinol and transport in blood while bound to retinol-binding protein, entering cells via the cell-surface receptor stimulated by retinoic acid 6.^291^ Intracellular retinol is metabolized into retinaldehyde by retinol dehydrogenases, then into transcriptionally active all-trans-RA (ATRA) by retinaldehyde dehydrogenases (RALDH1, 2, and 3).^292^ Alternatively, intracellular retinol can be esterified by lecithin:retinol acetyltransferase to be stored as lipid droplets, creating a reservoir for future RA synthesis.^292^ Retinoids are unusual among vitamins for being stored at high levels within tissues to provide a buffer against periods of dietary vitamin A deficiency; these stores can be mobilized locally to respond to tissue damage, including in the lung.^293^ Inappropriate RA signaling is limited in part through tight regulation of local RA concentration by cellular retinoic acid binding protein (CRABP) 1, which transports RA to cytoplasmic cytochrome P450 26 enzymes (CYP26A1, B1, and C1) for degradation.^294^

Intracellular RA undergoes nuclear import by CRABP2, whereupon RA interacts with cognate receptors of the nuclear receptor family, retinoic acid receptors (RAR-*α*, -*β*, and -*γ*) to drive transcription.^292^ RARs reside at RA response elements in regulatory regions of target genes as heterodimers with RXR-*α*, -*β*, and -*γ*. Unliganded RAR:RXR heterodimers repress transcription through interactions with nuclear receptor corepressors (NCOR1 and NCOR2), which together with histone deacetylases and Polycomb proteins mediate chromatin compaction and gene silencing.^292^ ATRA binding causes displacement of corepressors by nuclear coactivators (NCOA1, 2, and 3), which recruit histone acetyltransferases and trithorax proteins (mixed lineage leukemia family) to mediate chromatin relaxation and activation of a diverse set of genes.^292^

##### a Retinoic acid signaling control of lung cellular function

In vitro and in vivo studies have revealed that RA can exert powerful and cell-type specific effects on the major cell types of the distal lung. Cultured interstitial fibroblasts isolated from human or rat lungs increased synthesis of elastin, a key component of alveolar septa, in response to ATRA treatment.^294^^,^^295^ RA has long been known to promote differentiation of tracheal airway epithelial cells in air-liquid interface cultures.^296^ A recent study found using organoids derived from adult distal lung tissue that ATRA promoted differentiation of distal lung epithelial progenitors including alveolar progenitors, whereas RA pathway inhibition blocked differentiation and promoted epithelial expansion.^297^ The arrested differentiation in expanded epithelial organoids after RA inhibition was partially rescued by subsequent treatment with ATRA combined with histone deacetylases inhibitors, suggesting agents that modulate chromatin accessibility at RA target genes could synergize with RA to improve regenerative outcomes in the lung.^297^ Another study found that RA signaling, potentially through RAR-*α*, promoted angiogenesis in isolated human lung microvascular ECs.^298^ Induction of angiogenesis is sufficient to induce regeneration in lung disease models^299^; together, this suggests that RA-driven angiogenesis could offer a potential strategy to induce lung regeneration. Accordingly, during lung development in mice, administration of vascular endothelial growth factor receptor 2 (VEGFR2) inhibitors, which block angiogenesis caused alveolarization defects that were rescued with exogenous ATRA.^300^

The lung is rich in retinoid esters, which were long thought to be stored in lipid-laden interstitial fibroblasts.^301^ A recent study found that many additional lung cell types including the microvascular endothelium and AT 2 cells possess retinoid-containing lipid droplets.^293^ Importantly, using a model of LPS-induced acute lung injury, this study found that mobilization of local retinoid stores is an immediate response to tissue damage that is critical for successful resolution and survival.^293^ It would be of interest to further investigate signals that trigger retinoid store mobilization after lung tissue damage.

##### b Retinoic acid in lung development, adult tissue maintenance, and chronic lung disease

A key role for RA in lung development was revealed by mouse studies where targeted mutations in genes encoding RAR-*α*, -*β*, or -*γ* caused impaired lung development including alveolarization defects.^302^, ^303^, ^304^ In humans, vitamin A deficiency led to reduced lung function in offspring that was alleviated by maternal vitamin A supplementation.^305^ Moreover, genetic studies in humans have identified associations between variants in numerous RA pathway genes and adult lung function including RARA, RARB, and NCOR2,^306^, ^307^, ^308^ which could reflect the role of RA signaling in lung development, but could also reflect a requirement for RA signaling in maintaining adult lung tissue integrity. A recent human study found that carotenoid intake and serum carotenoid levels in adults positively correlated with lung function, suggesting a protective role for RA signaling in adult lung maintenance.^308^ Accordingly, low serum carotenoids were associated with increased risk of COPD.^309^ It has long been recognized that vitamin A deficiency in adult rats can lead to parenchymal defects including emphysematous changes.^310^ Thus, dysregulated RA signaling may be causal in the development of chronic lung disease. In support of this, emphysematous lung tissue showed increased expression of CYP26A1, which could increase local RA catabolism.^298^ In addition, fibroblasts isolated from emphysematous lung had reduced levels of cellular retinoic acid binding protein 2 and failed to upregulate elastin in response to ATRA treatment.^294^ Importantly, these changes may need to be overcome for the diseased lung to respond to exogenous RA.

##### c Pharmacological treatment with retinoic acid

Among the first to explore regenerative pharmacology in the lung were studies from the 1990s and 2000s in which RA was administered in preclinical rodent models of chronic lung disease. ATRA administered in a rat model of elastase-induced emphysema induced lung regeneration, increasing alveolar numbers and restoring tissue architecture.^311^ Subsequent studies in adult rodent models of emphysema supported these findings.^312^^,^^313^ Other studies failed to find an effect,^314^^,^^315^ perhaps owing to differing sensitivities of different animal strains to retinoids.^316^^,^^317^ Nonetheless, this initial excitement led rapidly to human clinical trials for RA in chronic lung disease. Two studies investigated orally administered ATRA for patients with advanced emphysema, but failed to find an effect on CT, lung function, or quality of life scores.^318^^,^^319^ The reasons for the failures remain unclear but could be attributed to the advanced disease stage of the participants, where severe structural damage, depletion of progenitor cells, or a hostile local tissue microenvironment may have provided a barrier to therapeutic efficacy.^33^ Moreover, it is unclear whether orally administered ATRA can reach the alveolar niche in sufficient quantities to drive repair.

It is possible that genes activated by RA during lung development are silenced in the aging lung, and exogenous ATRA alone might be unable to overcome this. Further studies to characterize epigenetic changes in the aging lung, and investigations into combining ATRA with approaches that modulate chromatin accessibility, may be warranted.^297^ Encouragingly, a recent study showed that although lung regeneration after partial pneumonectomy was strongly impaired in aged mice, lung cells of aged mice remained responsive to exogenously administered ATRA, which indirectly activated PDGFRA signaling within resident PDGFR*α*+ alveolar fibroblasts, thereby augmenting alveolar regeneration.^320^

##### d Synthetic retinoids

Although ATRA has been in use therapeutically since the 1980s, known issues are off-target side effects and instability in solution.^292^ Novel, synthetic retinoid derivatives that are stable and modulate discrete points in the RA pathway thus hold appeal for regenerative pharmacology. For example, the synthetic RAR-*γ*-selective agonist palovarotene is primarily degraded by CYP3A4 enzymes and thus likely unaffected by increased CYP26A1 found in emphysematous lung.^298^ Palovarotene was investigated in a parallel-group, placebo-controlled trial in patients with emphysema due to *α*1-antitrypsin deficiency. Palovarotene appeared to cause small improvements in lung density and lung function relative to placebo, which although it failed to reach statistical significance, may indicate biological activity.^321^ Other synthetic retinoids have been developed that specifically modulate the activity of RARs, CRABP1 and CRABP2, lecithin:retinol acetyltransferase, and CYP26 enzymes.^322^, ^323^, ^324^ Proof-of-concept studies in lung cells in vitro could probe the efficacy of such compounds to help shed light on their potential to promote regeneration in chronic lung disease.

#### 6 WNT pathway modifiers

**Fig. 3:**
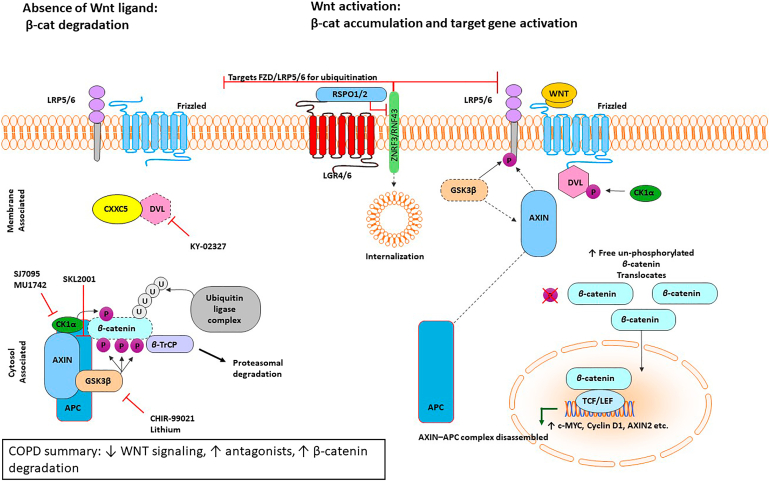
Canonical WNT/*β*-catenin signaling: mechanisms of activation, degradation and pharmacological modulation. In the absence of WNT ligand (left), cytosolic *β*-catenin is sequentially phosphorylated by CK1*α* and GSK-3*β* within the *β*-catenin destruction complex, which also includes axis inhibition protein (AXIN) and adenomatous polyposis coli (APC). Phosphorylated *β*-catenin is recognized by the E3 ubiquitin ligase adaptor *β*-transducin repeat–containing protein (*β*-TrCP), leading to polyubiquitination and proteasomal degradation. Upon WNT ligand engagement (right), the coreceptors Frizzled (FZD) and LRP5/6 cluster at the membrane, promoting CK1*α*-dependent phosphorylation and activation of DVL. This results in AXIN recruitment to the membrane, disassembly of the destruction complex, and stabilization of unphosphorylated *β*-catenin. Stabilized *β*-catenin accumulates in the cytosol, translocates into the nucleus, and interacts with T cell factor/lymphoid enhancer factor (TCF/LEF) transcription factors to activate WNT target genes.

WNTs are a family of secreted glycoproteins that act as ligands for receptors and play crucial roles in cell-to-cell communication, especially during development, tissue regeneration, and stem cell maintenance. WNTs (19 distinct members in humans) bind and activate cell surface receptors called Frizzled receptors (FZD_1_ through FZD_10_).^325^ The binding of individual WNT ligands to specific Frizzled receptors, in conjunction with coreceptors, can elicit the activation of distinct signal transduction pathways. The WNT signaling pathways are mainly categorized as being *β*-catenin-dependent (classically referred to as canonical WNT signaling) or *β*-catenin-independent (ie, noncanonical WNT signaling).^325^ In the absence of an extracellular WNT signal, cytosolic *β*-catenin is targeted for degradation by the so-called *β*-catenin destruction complex. Glycogen synthase kinase-3*β* (GSK-3*β*) plays a central role in the *β*-catenin destruction complex, serving as the main kinase that phosphorylates *β*-catenin, thereby targeting it for ubiquitination and proteasomal degradation. The destruction complex also includes several core components: axis inhibition protein (AXIN), a scaffold protein and the rate-limiting factor of the complex; adenomatous polyposis coli, a tumor suppressor protein; and casein kinase 1*α* (CK1*α*), which initiates *β*-catenin phosphorylation, priming it for further phosphorylation by GSK-3*β*. Specific WNTs (eg, WNT-3A) bind to specific FZD receptors and the coreceptors low-density lipoprotein-related receptors 5 and 6 (LRP5/6), resulting in inactivation of the destruction complex. Consequently, *β*-catenin degradation is reduced, it accumulates in the cytosol and subsequently translocates to the nucleus.^326^ Nuclear *β*-catenin associates with T-cell factor/lymphoid enhancer factor transcription factors to regulate gene expression (Fig. 3).

WNT signaling plays a crucial role in the development and maintenance of lung progenitor cells, particularly AT2 cells. It is essential for the proliferation and self-renewal capacity of AT2 cells, which are key for alveolar homeostasis and regeneration after lung injury.^327^ AXIN2 functions as a negative feedback regulator, mediating *β*-catenin degradation and promoting commitment of AT2 cells to differentiation toward AT1 cells.^327^^,^^328^ Importantly, AXIN2 expression peaks within WNT-active progenitor niches, establishing spatial gradients that define zones of self-renewal versus differentiation.^327^ AT2 cells adjacent to WNT-secreting fibroblasts (eg, WNT-3A and WNT-5A) maintain progenitor features, whereas AT2 cell positioned distally tend to differentiate into mature AT1 cells.^50^^,^^67^^,^^329^ Disruption of WNT signaling gradients in COPD may lead to depletion or senescence of AT2 progenitor cells, thereby impairing their ability to self-renew and progress toward an AT1-like fate.^330^ Restoring balanced WNT signaling is beneficial for lung tissue regeneration and, consequently, may offer therapeutic potential for the treatment of COPD.^330^ However, excessive or prolonged *β*-catenin activation disrupts normal alveolar epithelial maturation dynamics in vitro^329^ and the consequences of such overactivation on alveolar epithelial cell lineage progression in vivo remains incompletely defined.

##### a Glycogen synthase kinase-3 inhibitors

GSK-3*β* is the primary kinase responsible for the phosphorylation of the WNT effector protein *β*-catenin. Upon phosphorylation, *β*-catenin is ubiquitinated and targeted for proteasomal degradation.^325^ In COPD, *β*-Catenin expression is downregulated, particularly in the alveolar epithelium.^330^ Pharmacological inhibition of GSK-3*β* leads to stabilization of *β*-catenin and activation of *β*-catenin-mediated gene transcription in various lung cells, with beneficial effects observed in multiple preclinical models of COPD. For example, lithium chloride, an US Food and Drug Administration (FDA) approved drug used for the treatment of bipolar disorder, activates *β*-catenin signaling and has been shown to reduce elastase-induced emphysema in mice.^330^ This therapeutic effect was recapitulated in 3-dimensional ex vivo lung tissue cultures derived from patients with COPD.^330^ Similarly, the structurally unrelated GSK-3*β* inhibitor CHIR/CT99021, also known as Laduviglusib, activated *β*-catenin signaling in lung tissue cultures of patients with COPD. Therapeutic application of CHIR/CT99021 also reduced CS-induced emphysema in mice.^331^ Notably, pharmacological activation of *β*-catenin signaling by CHIR/CT99021 can partially restore the impaired function of distal lung progenitor cells, including AT2 cells, in experimental emphysema models.^332^ In addition, SB216763, another GSK-3*β* inhibitor, has demonstrated protective effects in a guinea pig model of lipopolysaccharide-induced pulmonary inflammation. In this model, which mimics aspects of COPD, treatment with SB216763 improved both lung pathology and skeletal atrophy.^333^^,^^334^ In addition to *β*-catenin activation, GSK-3*β* inhibitors also suppress NF-*κ*B signaling, a key driver of COPD-related inflammation.^335^ This might also be relevant for COPD pathogenesis as particulate matter (PM2.5) and CS-induced inflammatory responses in vitro were suppressed by SB216763 via suppression of NF-*κ*B signaling.^336^^,^^337^ Together, these findings support the therapeutic potential of GSK-3*β* inhibitors in COPD. GSK-3*β* is required for proper proliferation and maturation of lung epithelial progenitors both, and the timing of its inhibition appears essential for achieving regenerative benefit.^338^^,^^339^ In a murine model of inflammatory lung injury, transient GSK-3*β* inhibition alleviates LPS-induced damage and promotes epithelial repair.^338^ GSK-3 inhibition produced distinct effects on alveolar epithelial cell proliferation and differentiation depending on whether GSK-3 was blocked during the acute inflammatory phase or during the postacute inflammatory phase after LPS-induced injury.^338^ However, prolonged or sustained GSK-3*β* inhibition can impair the terminal differentiation of lung progenitors in vitro.^339^ Collectively, these findings indicate that both dosage and timing of GSK-3 inhibition are critical to harness regenerative benefit. Alongside the aforementioned GSK-3 inhibitors, there are many more small molecules that inhibit GSK-3*β* and their therapeutic potential is being investigated for various disease conditions, but without any available data for COPD.^340^

##### b Alternative mechanisms of β-catenin activation

In addition to classical GSK-3*β* inhibition, several drugs have been identified that activate *β*-catenin signaling through alternative mechanisms. For instance, the FDA-approved anti-inflammatory drug amlexanox and the pain reliever phenazopyridine hydrochloride both promote organoid formation in a *β*-catenin-dependent manner. Importantly, amlexanox has shown therapeutic efficacy in vivo by significantly reducing elastase-induced emphysema in a mouse model of COPD.^341^ However, the activation of *β*-catenin by these drugs is most likely not related to direct inhibition of GSK-3*β*.

In addition to specific WNTs that activate *β*-catenin-dependent signaling, other WNTs can activate alternative (noncanonical) signaling pathways. In this context, WNT-5A is of particular interest, as its effect on *β*-catenin signaling depends on the receptor environment at the cell membrane. In the presence of the FZD_4_ receptor, WNT-5A activates *β*-catenin-dependent signaling. FZD_4_ is involved in *β*-catenin–driven alveolar lung repair and is significantly downregulated in human and experimental COPD.^342^ In contrast, WNT-5A expression itself is upregulated in COPD and is associated with reduced *β*-catenin activity in alveolar epithelial cells.^343^ Therapeutic targeting of this pathway has yielded promising results. Inhibition of WNT-5A using either a neutralizing antibody or BOX5, a WNT-5A-derived, N-terminally butyloxycarbonyl-(Boc) protected hexa-peptide, attenuated lung tissue destruction, improved lung function, and restored expression of *β*-catenin-driven target genes and alveolar epithelial cell markers. These effects have been demonstrated in both elastase- and CS-induced models of COPD.^343^ Collectively, these findings suggest that restoring FZD4 expression or inhibiting WNT-5A may provide therapeutic benefit in emphysema by reactivating *β*-catenin signaling. In addition, WNT-5A has profibrotic actions by enhancing fibroblast-to-myofibroblast differentiation and activation of the profibrotic growth factor latent TGF-*β*.^342^^,^^344^ Although GSK-3 inhibition primarily enhances canonical WNT/*β*-catenin signaling and WNT-5A predominantly engages noncanonical pathways, crosstalk exists whereby canonical WNT activation can mitigate certain profibrotic effects of WNT-5A signaling, and vice versa.

##### c WNT ligand and Frizzled receptor modulators

Direct activation of canonical WNT signaling using ligand mimetics is emerging as a promising regenerative strategy in chronic lung diseases. One such approach involves the antibody R2M3-26, which has been engineered to simultaneously engage multiple Frizzled receptors (FZD_1_, FZD_2_, FZD_5_, FZD_7_, and FZD_8_) along with the LRP6 coreceptor. This multivalent targeting functionally mimics natural WNT ligands and activates *β*-catenin signaling across diverse target cells. A recent study demonstrated that R2M3-26 significantly enhanced alveolar organoid expansion in vitro using both mouse and human-derived AT2 cells.^345^ In vivo, R2M3-26 treatment in mice with bleomycin-induced pulmonary fibrosis reduced inflammation and collagen deposition, improved lung mechanics (increased lung compliance and decreased elastance), and upregulated Axin2 in epithelial, mesenchymal, and endothelial compartments, indicating widespread WNT pathway activation and tissue repair.^345^

Building on this strategy, receptor-specific agonists targeting individual FZD subtypes have also shown efficacy in modulating epithelial regeneration. FZD_5_- and FZD_6_-specific agonist antibodies were recently shown to potently activate canonical WNT/*β*-catenin signaling in AT2 cells, enhancing their stem cell activity.^346^ In this study, FZD_5_ was identified as essential for AT2 self-renewal and epithelial regeneration after injury. Interestingly, FZD_6_, which is traditionally associated with noncanonical signaling, was also found to activate *β*-catenin–dependent transcription in AT2 cells. Systemic administration of FZD_5_- or FZD_6_-specific agonists in vivo promoted AT2 proliferation and improved survival in bleomycin-treated mice.^346^ In a murine emphysema model, systemic delivery of WNT-3A loaded EVs enhanced AT2 cell proliferation, reduced alveolar space enlargement, and improved lung function.^65^ Notably, this approach also led to activation of regenerative gene programs across epithelial, mesenchymal, and endothelial compartments. Taken together, these findings support the feasibility of ligand-based WNT activation as a regenerative strategy in COPD. This approach carries important context-dependent considerations. GSK-3 inhibition may act synergistically with FZD-targeted agonists to enhance *β*-catenin activation and thereby promote epithelial regeneration. A key caveat is that GSK-3 intersects with multiple WNT pathways: depending on the cellular context, GSK-3 (inhibition) can also modulate noncanonical WNT5A-driven signaling, which has been linked to prosurvival and profibrotic responses in fibroblasts.^347^ Consequently, combining GSK-3 inhibition with FZD receptor agonism carries the potential for both proregenerative and profibrotic outcomes, depending on pathway bias and cellular context.^348^

##### d Casein kinase 1α inhibitors

CK1*α* is a regulatory kinase involved in the phosphorylation of several components within the *β*-catenin destruction complex. Inhibition of CK1*α* can stabilize *β*-catenin and thereby enhance WNT signaling. Although specific studies in COPD models are limited, the modulation of CK1*α* presents a potential strategy for restoring epithelial regeneration in chronic lung diseases. One example is SJ7095, a recently developed molecular glue degrader of CK1*α*. This compound induces a specific interaction between an E3 ubiquitin ligase and the target protein, leading to its targeted degradation.^349^ Whereas SJ7095 has shown promise in modulating WNT signaling through CK1*α* degradation, it remains to be tested in COPD models. Another compound, MU1742, has also been identified as a CK1*α* inhibitor with potential WNT-activating properties.^350^ Despite their promise, targeting CK1*α* carries important considerations. Similar to GSK-3*β*, CK1*α* is involved in a diverse array of cellular processes, including circadian rhythm regulation, DNA repair, and apoptosis.^351^ Additionally, CK1*α* also contributes to NF-*κ*B activation. Its inhibition may therefore suppress inflammatory pathways, potentially dampening immune responses and impairing host defense mechanisms. This is an important consideration given the heightened susceptibility to infections in patients with COPD.^352^ Taken together, although CK1*α* inhibitors represent a mechanistically compelling route to restore WNT activity and promote epithelial regeneration, their pleiotropic effects warrant careful evaluation in the context of COPD.

##### e Dishevelled activators/stabilizers

Dishevelled proteins (DVL1, 2, and 3) are central scaffolds in the WNT signaling cascade. They transmit signals from FZD receptors to downstream effectors, including *β*-catenin, and participated in both *β*-catenin-dependent and -independent pathways through their Dishevelled and Axin (DIX), PSD-95, Dlg1, and ZO-1 (PDZ), and Dishevelled, EGL-10, and Pleckstrin (DEP) domains.^325^ Within the canonical WNT pathway, DVL contributes to *β*-catenin stabilization by inhibiting the *β*-catenin destruction complex, thereby enabling transcription of WNT target genes.

Pharmacological activation or stabilization of DVL has been proposed as a strategy to reinforce canonical WNT signaling and promote alveolar epithelial repair. One known negative regulator of DVL is CXXC5, which binds to the PDZ domain of DVL and attenuates *β*-catenin signaling.^353^ Small-molecule inhibitors such as KY-02061 and KY-02327 block this interaction. By preventing the binding of CXXC5 to DVL, these compounds relieve negative feedback inhibition and enhance WNT pathway activation.^354^ These findings suggest that stabilizing DVL activity through targeted disruption of inhibitory protein interactions may represent a promising therapeutic approach to promote epithelial regeneration in chronic lung diseases such as COPD.

##### f Secreted Frizzled-related proteins

Secreted Frizzled-related proteins (sFRPs) are extracellular antagonists of WNT signaling that function by binding and sequestering WNT ligands, thereby preventing their interaction with FZD receptors.^325^ Among the sFRP family members, sFRP1 and sFRP2 have been implicated in the pathogenesis of COPD by contributing to impaired epithelial repair. sFRP1 is elevated in emphysematous lung tissue and correlates with increased expression of MMP-1 and MMP-9, implicating a role in ECM degradation and alveolar destruction.^355^ Similarly, sFRP2 expression is increased in the small airway epithelium of smokers and patients with COPD. This protein suppresses *β*-catenin signaling and may thereby hinder epithelial regeneration by interfering with canonical WNT pathway activity.^356^ Given this inhibitory role, neutralizing sFRPs has been explored as a strategy to restore WNT signaling. Antibody-mediated blockade of SFRP1 has been shown to relieve extracellular WNT inhibition: in stressed epithelial and fibroblast systems, anti-SFRP1 antibodies attenuated SFRP1-dependent senescence and restored downstream *β*-catenin signaling.^357^ Furthermore, small-molecule approaches have also been developed to target sFRPs. Bodine et al^358^ identified sFRP-1 inhibitors (eg, WAY-316606) through high-throughput screening, demonstrating that direct pharmacological inhibition of sFRP-1 selectively increases *β*-catenin activity in functional reporter assays.^358^ Although this data originates from skeletal biology, they provide proof-of-concept that pharmacologically releasing extracellular WNT brakes is feasible. In addition, intracellular DVL activators (eg, KY-02061 and KY-02327) can bypass extracellular ligand sequestration entirely by relieving CXXC5-mediated inhibition of DVL, restoring downstream signaling even under conditions of elevated sFRP expression.

##### g R-spondin proteins and R-spondin agonists

R-spondins (RSPO1 to RSPO4) are secreted glycoproteins that enhance *β*-catenin–dependent WNT signaling. They function by binding to leucine-rich repeat–containing G protein-coupled receptors (LGR4/5/6) and inhibiting the E3 ubiquitin ligases ZNRF3 and RNF43. This interaction prevents internalization and degradation of WNT receptors, thereby stabilizing Frizzled and LRP5/6 on the cell surface and amplifying WNT signal transduction.^359^ In the lung, RSPO2 is involved in epithelial patterning and branching morphogenesis during development, suggesting a potential role in progenitor cell regulation.^360^ In a murine model of bleomycin-induced lung injury, RSPO2 administration enhanced WNT target gene expression and accelerated epithelial repair, further supporting its regenerative potential.^361^ Despite these promising findings, the clinical application of recombinant RSPO proteins is limited owing to issues with protein stability and delivery. To address these limitations, recent research has focused on developing small-molecule agonists that mimic RSPO activity by targeting LGR4/5/6 receptors. Unfortunately, the development of small-molecule LGR4 agonists as RSPO mimetics has thus far not replicated RSPO’s effect on *β*-catenin–dependent WNT signaling. In one study, a *β*-arrestin-biased LGR4 agonist (referred to as compound 1) failed to enhance T-cell factor and *β*-catenin reporter activity and instead slightly antagonized RSPO1-mediated signaling.^362^ These findings suggest that current LGR4-targeting small molecules act via *β*-catenin-independent pathways, limiting their applicability for regenerative strategies aiming to restore alveolar *β*-catenin activity in COPD.

### B Small molecules that interfere with chronic obstructive pulmonary disease-specific regenerative defects

#### 1 Senotherapeutics

The incidence of COPD increases with age and is closely linked to the hallmarks of aging.^6^^,^^363^ Although genetic alterations in aging pathways are not well established in COPD, hallmarks of aging likely emerge from disease progression or environmental exposures such as CS.^364^ Individuals with early-life lung impairment are particularly susceptible to accelerated aging.^363^^,^^365^^,^^366^ Patients with COPD consistently show elevated markers of aging, with cellular senescence being among the most prominent.

Senescence is a stress-induced, irreversible cell-cycle arrest state associated with senescence-associated secretory phenotype (SASP) secretion, which drives chronic inflammation and induces senescence in neighboring cells.^367^^,^^368^ Senescent cells have been identified within several lung compartments, including AT2 cells,^60^^,^^369^ airway epithelium,^370^ endothelium,^371^^,^^372^ smooth muscle cells,^372^^,^^373^ and fibroblasts.^162^^,^^188^ Their SASP, in part mediated by EVs, promotes paracrine inflammation, tissue remodeling, and immune dysregulation in COPD.

CS-induced oxidative stress accelerates senescence via telomere shortening, DNA and mitochondrial damage, and activation of the ATM-p53-p21 and p16-Rb pathways.^374^ Accumulated senescent cells impair lung repair and sustain inflammation. Targeting senescence (senotherapy) is a promising therapeutic avenue in age-related diseases, including COPD,^367^^,^^375^ with 2 main strategies under investigation: elimination of senescent cells (senolytics) or functional reprogramming (senomorphics).

##### a Senolytics

**Fig. 4:**
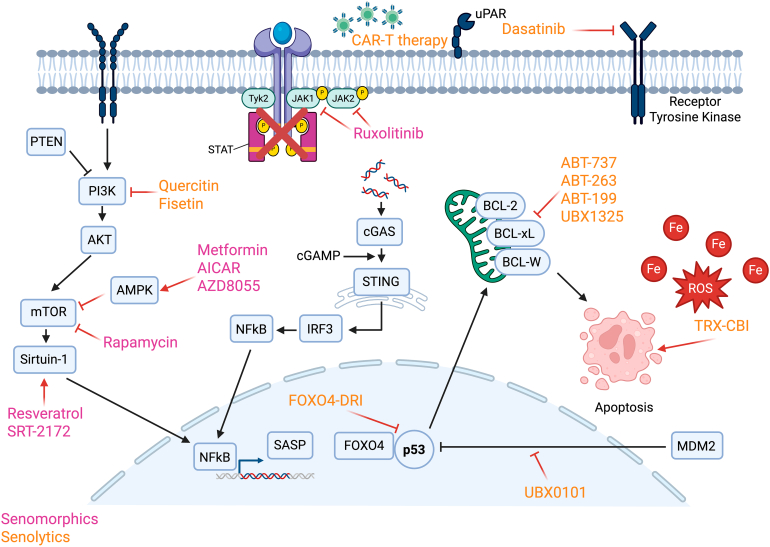
Mode of action of senotherapeutics. Cellular senescence is characterized by changes in mTOR signaling, NF-*κ*B signaling, cyclic GMP-AMP synthase (cGAS)/Stimulator of Interferon Genes (STING) signaling, mitochondrial dysfunction, and oxidative stress. Commonly used senotherapeutics target one or more of these pathways to balance cellular signaling (senomorphics) or to eliminate senescent cells (senolytics).

Senolytics are compounds that target and eliminate senescent cells by disrupting their resistance to apoptosis. These cells rely on antiapoptotic pathways to survive; senolytics reactivate programmed cell death specifically in these cells, enabling their clearance by the immune system. The idea of removing senescent cells to promote healthy aging originated from studies using the INK-ATTAC mouse model, where genetic ablation of p16(INK4a)-positive cells led to increased lifespan and reduced cancer incidence.^376^ This foundational work inspired the development of drug screening platforms to identify senescence-targeting therapies. First-generation senolytics primarily targeted survival pathways, whereas newer agents focus on senescence-specific surface markers and phenotypes to enhance selectivity (Fig. 4). The concept of depleting senescent cells is a promising concept since this would not require constant medication of the patient but a hit and run approach where the drugs can be administered intermittently.

The combination of dasatinib and quercetin (D+Q) remains one of the most extensively studied senolytic regiments.^377^ Dasatinib, a tyrosine kinase inhibitor with selectivity for Abl and Src, and quercetin, a polyphenol, selectively eliminate senescent cells in vitro. D+Q are under clinical investigation for aging-related diseases, including idiopathic pulmonary fibrosis, with promising feasibility and safety data.^378^^,^^379^

Although no clinical data yet exist for senolytics in COPD, preclinical evidence is accumulating. Quercetin alone reduced inflammation and disease progression in elastase/lipopolysaccharide (LPS)-induced emphysema models, though its senolytic effect was not assessed.^380^ Recent preclinical studies show that D+Q reduces CS–induced senescence, inflammatory cell infiltration, and cytokine levels in COPD mouse models.^381^ Similarly, D+Q reduced senescence and inflammation in air-liquid interface cultures from patients with COPD.^381^ A randomized trial confirmed quercetin safety in patients with COPD, though without assessing cellular senescence markers.^382^

A prominent target of senolytic strategies is the antiapoptotic BCL-2 family, which is often upregulated in senescent cells (Fig. 4). Compounds such as navitoclax (ABT-263), venetoclax (ABT-199), and ABT-737 mimic BH3 proteins by binding and inhibiting prosurvival proteins like B-cell lymphoma (BCL)-2, BCL-XL, and BCL-w. These agents show differential efficacy across senescent fibroblasts, partially restoring ECM regulation in vitro.^383^ Navitoclax also effectively eliminated senescent AT2 cells from patients with COPD.^384^ Cardiac glycosides, including ouabain and digoxin, have been identified as effective senolytics, acting in part through induction of the proapoptotic BCL-2 family member NOXA and by disrupting the intracellular sodium–potassium gradient.^385^^,^^386^ Although their senolytic efficacy has been demonstrated in animal models of fibrosis, no data are currently available regarding their use in models of COPD. Newer, potentially safer BCL-2 inhibitors such as UBX1325 have demonstrated senolytic activity, leading to improved retinal function and macular thickness in diabetic macular edema.^387^

Additionally, PROteolysis TArgeting Chimera (PROTAC)-based approaches that target BCL-XL for degradation via E3 ligases show promise in reducing senescence and inflammation while promoting proliferation in COPD small airway epithelial cells.^388^ Natural compounds such as fisetin, a flavonoid with anti-inflammatory properties,^389^ have also shown preliminary senolytic effects in COPD epithelial cells.^390^

Of note, tight regulation of proapoptotic and antiapoptotic signaling determines cell fate during tissue remodeling and repair.^391^ Apoptosis of epithelial cells has been described as a pathogenic mechanism in COPD,^392^^,^^393^ whereas upregulation of antiapoptotic pathways can exert protective effects on epithelial cells. This underscores that first-generation senolytics targeting proapoptotic pathways must be applied with caution to preserve a finely tuned apoptotic balance that supports tissue regeneration.

To enhance specificity, second-generation senolytics exploit unique features of senescent cells. One strategy targets increased lysosomal content and senescence-associated *β*-galactosidase activity using galacto-oligosaccharide-coated nanoparticles or *β*-gal-activated prodrugs.^394^^,^^395^ Another approach leverages iron dysregulation, a hallmark of senescent cells, which contributes to fibrosis and inflammation.^396^ Iron-activated prodrugs such as trioxolane-cyclopropylbenzindoline conjugate (TRX-CBI) selectively eliminate iron-overloaded senescent cells,^397^ a relevant strategy given the elevated iron levels in COPD and senescent airway cells.^375^^,^^398^ Forkhead box O (FOXO)4, a longevity-associated transcription factor, binds p53 in senescent cells to block apoptosis. Disrupting this interaction with FOXO4-D-Retro-Inverso (DRI) peptide induces senescent cell death and reduced fibrosis in an experimental fibrosis model.^399^ In CS-induced senescent lung fibroblasts, DNA nanoparticles targeting Foxo4 displayed senolytic activity.^400^

Senescent cells also show mitochondrial dysfunction and increased glutaminolysis. Elevated glutaminase-1 breaks down glutamine into glutamate and ammonium, supporting survival. Inhibiting glutaminase-1 with bis-2-(5-phenylacetamido-1,2,4-thiadiazol-2-yl)ethyl sulfide (BPTES) induces senolysis and improves age-related organ function in mice.^401^ Preliminary data suggests that it displays senolytic activity on senescent airway epithelial cells.^402^

Alternative strategies to reduce senescent cells and their impact on chronic diseases include enhancing immune clearance or reactivating aging immune responses (Fig. 4). Chimeric antigen receptor (CAR) T cell therapies, initially developed for cancer, have been adapted to target senescent cells using surface markers such as urokinase-type plasminogen activator receptor (uPAR) and natural killer group 2, member D (NKG2D) ligands, showing efficacy in preclinical models—though uPAR is unsuitable for the lung due to its broad expression on nonsenescent cell types including immune cells, endothelial and epithelial cells.^403^^,^^404^ In COPD and obesity, senescent T cells contribute to chronic inflammation, and targeted elimination, by vaccination against CD153^+^ T cells, has improved tissue function in models,^405^ suggesting immune modulation may offer therapeutic potential in chronic lung diseases.

Notably, senescent cells play essential roles in normal embryonic development, tumor suppression, and wound healing.^406^ In the adult murine lung, senescent fibroblasts have been shown to be required for epithelial regeneration.^407^ Conversely, indiscriminate targeting of senescent cells may have adverse effects on wound healing and tissue homeostasis, emphasizing the need for strategies that specifically eliminate pathologically senescent cells while preserving their physiological functions.

##### b Senomorphics

Senomorphics, which modulate senescence-associated pathways without directly eliminating senescent cells, represent a promising therapeutic strategy by attenuating chronic inflammation and tissue remodeling driven by the SASP. Key agents include mechanistic target of rapamycin (mTOR) inhibitors, Sirtuin activators, and Janus kinase (JAK)/signal transducer and activator of transcription (STAT) inhibitors. mTOR signaling regulates metabolism, proliferation, and senescence and is implicated in longevity. Its pharmacological inhibition has extended lifespan in model organisms.^408^ In emphysema models, rapamycin reduced senescence markers.^371^ Metformin, a widely used antidiabetic drug, activates AMP-activated protein kinase (AMPK) and inhibits mTOR signaling, thereby reducing oxidative stress and SASP-driven inflammation in airway epithelial cells and ECs,^409^^,^^410^ and protected mice from CS-induced injury in lung, kidney, and muscle.^409^ Retrospective cohort analyses suggest clinical benefits of metformin in COPD.^409^^,^^411^^,^^412^ JAK/STAT inhibition prevented senescence in emphysema models,^372^ with inhaled delivery improving tolerability and reducing SASP.^413^

Sirtuins (SIRT1, SIRT3, and SIRT6), NAD^+^-dependent deacetylases, regulate inflammation, senescence, and mitochondrial function, playing critical roles in chronic lung disease progression.^414^, ^415^, ^416^ Resveratrol, a sirtuin activator with lifespan-extending effects,^417^ has poor pharmacokinetic profile, prompting the development of more potent analogs as well as synthetic SIRT activators to reduce CS-induced lung inflammation. NAD^+^ supplementation, for example, via nicotinamide riboside, increased NAD^+^ levels and reduced lung inflammation and senescence markers in patients with COPD in a recent clinical trial.^418^

Modulating the SASP by targeting EVs, which are key SASP carriers, offers another novel therapeutic approach. Beyond targeting senescence, additional aging-related mechanisms in chronic lung disease include mitochondrial reactive oxygen species inhibition (eg, MitoQ and SkQ1), autophagy/mitophagy activation, and epigenetic modulation.^419^, ^420^, ^421^ Modifying the gut-lung microbiome or using epigenetic clocks as biomarkers further expands options for noninvasive monitoring and intervention. Collectively, targeting the hallmarks of aging may not only slow COPD progression but also promote lung regeneration after injury.

#### 2 Rho-associated coiled-coil kinase inhibitors

Rho-associated coiled-coil kinase (ROCK) has a role in interfering with fibroblast function and differentiation toward myofibroblasts. Lung fibroblasts are required for alveolar epithelial regeneration by secreting growth factors, by producing ECM, and providing mechanical support. Cytokines such as TGF-*β* promote a myofibroblast phenotype, which is less able to support lung organoid formation.^422^^,^^423^ Although TGF-*β* is primarily associated with lung fibrosis, its levels are increased in COPD lung tissue as well.^424^ The main mechanisms via which TGF-*β* pretreatment of fibroblasts restricts the support function of mesenchymal cells includes modulation of WNT pathway signaling as well as actin cytoskeletal remodeling. Thus, TGF-*β*–induced impairment of lung organoid formation can be mimicked by pretreating lung fibroblasts with jasplakinolide, which enhances actin cytoskeletal stiffening.^425^

Actin cytoskeletal remodeling depends on the conversion of globular actin to filamentous actin, which promotes the formation of stress fiber-like bundles of smooth muscle *α*-actin. This process also enhances smooth muscle *α*-actin gene transcription by releasing G-actin–bound transcriptional regulators, such as myocardin-related transcription factor (MRTF)-A, which translocate to the nucleus upon actin polymerization to activate target gene expression.^426^ TGF-*β* and other actin factors that promote actin remodeling such as WNT-5A and WNT-11 utilize signaling via ROCK to enhance actin remodeling and subsequent MRTF-A dependent smooth muscle *α*-actin expression.^427^^,^^428^ Accordingly, ROCK inhibitors are potential antagonists of actin cytoskeletal remodeling and its downstream effects. The most widely studied ROCK inhibitor is Y-27632, but other ROCK inhibitors are available, of which fasudil is even registered for clinical use in cerebral vasospasm.^429^

Similar beneficial effects can be achieved for epithelial cell growth. In fact, ROCK inhibitors such as Y-27632 are often provided during lung organoid cultures to enhance progenitor cell activation. For example, ROCK inhibition using Y-27632 enhanced alveolar epithelial cell growth, WNT pathway activation and expression of alveolar epithelial markers such as surfactant protein C.^430^ ROCK inhibitors are also able to reduce the negative effects of TGF-*β* on lung organoid formation, both if ROCK1/2 are simultaneously inhibited using compound A31 or when ROCK2 is inhibited selectively using compound A11.^425^ However, and in contrast to organoid number, the differentiation of lung organoids toward surfactant protein C (SFPTC)^+^ alveolar epithelial cells was repressed by TGF-*β* and not reversed by ROCK inhibition.^425^ The potential of ROCK inhibitors in reversing elastase-induced emphysema has not yet been reported, although CS-induced inflammation and vascular permeability have been shown to be ROCK-dependent.^431^ In addition, because of the effects of ROCK inhibition on myofibroblast differentiation, indirect effects on MMP production may be envisaged. In conclusion, although ROCK inhibitors show therapeutic promise, current data are insufficient to draw firm conclusions about their role in lung regeneration in COPD.

#### 3 Protease inhibitors

Lung emphysema is characterized by alveolar destruction, in part caused by an imbalance between proteases and antiproteases in the lung.^432^ This dysregulation leads to excessive proteolytic activity, resulting in ECM degradation and lung tissue damage. Extracellular proteases, particularly neutrophil elastase, MMPs, and cathepsins, are enzymes responsible for the degradation of matrix components such as elastin and collagens.^432^ As such, proteases play a physiological role in tissue remodeling, immune defense, and inflammatory responses. To prevent undesirable destruction of lung tissue, their activity must be tightly regulated by endogenous antiproteases, including *α*_1_-antitrypsin (A1AT), secretory leukocyte protease inhibitor, and tissue inhibitors of metalloproteinases, which neutralize proteolytic enzymes and maintain lung structural integrity.^432^^,^^433^

In COPD and emphysema, the protease/antiprotease imbalance favoring proteolytic activity results in excessive degradation of alveolar walls, leading to loss of elastic recoil, and emphysema development. This may be caused by exposure to toxic chemicals, particles and gases such as CS, which trigger neutrophilic and macrophage-driven inflammation, leading to the release of neutrophil elastase and MMPs, which if persistent, contributes to lung tissue damage.^434^ Moreover, oxidative stress generated by reactive oxygen species inactivates antiproteases such as A1AT, shifting the imbalance further. Genetic polymorphisms in the *α*_1_-antitrypsin gene, leading to *α*_1_-antitrypsin deficiency (AATD) is a well established genetic cause of COPD leading to early-onset emphysema.^435^

Targeting the protease–antiprotease imbalance for COPD and emphysema is an older concept, but with the exception of A1AT augmentation therapy for patients with AATD, these approaches have not yet reached the clinic. Partly, this may be explained by the complexity of the protease/antiprotease network, rendering inhibition of individual proteases insufficient for clinical efficacy. Nonetheless, understanding this dynamic interplay between proteases and antiproteases remains critical for developing novel interventions to halt or slow disease progression.

##### a α_1_-Antitrypsin augmentation therapy

AATD is a genetically driven form of emphysema characterized by reduced serum levels (below 80 mg/dL) of functional A1AT, a serine protease inhibitor primarily responsible for protecting lung tissue from neutrophil elastase-mediated degradation.^436^ The link between AATD and pulmonary emphysema led to the development of A1AT replacement as a potential therapeutic strategy. Initial efforts to develop augmentation therapy were reported in 1981, when Gadek et al^437^ found that weekly A1AT supplementation was able to restore A1AT levels to normal in affected individuals. Further developments led to the first FDA-approved plasma-derived intravenous A1AT therapy, Prolastin, which became available in 1987. Since then, additional formulations (eg, Aralast, Zemaira, and Glassia) have been introduced, to restore circulating levels in deficient individuals.^438^ A1AT augmentation therapy has demonstrated effects in reducing the progression of emphysema, by slowing the decline in lung density measured by CT imaging.^439^, ^440^, ^441^ Current guidelines recommend augmentation therapy for individuals with severe AATD (PiZZ or PiSZ genotypes) and clinically significant emphysema.^442^

Ongoing research is aimed at novel therapeutic strategies, including AAT replacement therapy using other administration routes and sources and gene therapy.^443^ PEGylated AAT is under development as an inhaled formulation, which is not feasible using regular AAT because of the rapid clearance. INBRX-101 is a recombinant human AAT-Fc fusion protein that was found to be well tolerated in patients with AATD in a phase I trial, which increased the plasma AAT levels as well as AAT levels in epithelial lining fluid.^444^ BEAM-302 is a lipid nanoparticle (LNP) formulation containing base editing reagents designed to correct the PiZ allele, which is now in phase I/II trials (NCT06389877). Clinical trials demonstrated safety with gene transfer of the *SERPINA1* gene that encodes for A1AT, though yet with limited efficacy.^445^ Since then, several attempts have been made to package the gene in adenoviral transduction systems for replacement expression of the functional gene, with variable success.^443^ However, the established safety of this approach is encouraging and suggest that adenoviral delivery holds promise for further optimization of effective gene therapy approaches.

##### b Matrix metalloproteinase inhibitors

MMPs are secreted proteolytic enzymes that are mainly provided by inflammatory cells such as macrophages, neutrophils, and T cells. These enzymes degrade ECM proteins such as collagens and elastin.^446^ In particular MMP-1, MMP-12, and MMP-28 have been demonstrated to contribute to emphysema development in mice,^119^^,^^447^^,^^448^ whereas MMP-1, MMP-2, MMP-3, MMP-7, MMP-8, MMP-9, MMP-10, MMP-12, and MMP-28 all have increased expression in COPD.^447^^,^^449^, ^450^, ^451^

Pharmacological intervention with dual inhibitors for MMP-9 and MMP-12 has been attempted, which led to successful inhibition of airway remodeling and emphysema development in CS-exposed guinea pigs using the inhibitor AZ11557272.^452^ The orally active dual MMP-9/MMP-12 inhibitor AZD1236 was evaluated in clinical trials in COPD, although not with emphysema progression or related outcomes as primary endpoints. The results showed no effects on inflammatory biomarkers such as differential cell counts and TNF-*α* levels in sputum, or in desmosine excretion in urine as a proxy for elastin breakdown.^453^ Another interesting strategy to inhibit MMP activity is to target the delivery of pentagalloyl glucose to the lung using inhaled nanoparticles loaded with this drug. This resulted in suppression of MMP-12 activity and the preservation of elastin integrity in the lungs of elastase treated mice.^454^ Collectively, although MMP inhibition appears effective in animal models, its clinical relevance remains to be established.

##### c Cathepsin inhibitors (including dipeptidyl peptidase 1 inhibitors)

Cathepsins are lysosomal proteases that have been implicated in the pathogenesis of COPD through their role in ECM degradation, inflammation, and tissue remodeling. Their activity is inhibited by cystatins, and the ratio of cathepsin to cystatin expression was found increased in plasma of patients with COPD and was found to correlate to the degree of emphysema.^455^ Cathepsin E expression is increased in COPD and its overexpression results in the activation of cell death and emphysema development in mice.^456^ Cathepsin C is also known as dipeptidyl peptidase 1 (DPP-1), and contributes to lung tissue damage as well. Inhibitors of DPP-1 are under development mainly for bronchiectasis^457^ but may be interesting to pursue as a therapeutic strategy for COPD too, given the protective effects of DPP-1 inhibition in animal models of COPD.^458^ This is not only because of the direct effects on tissue damage, but also because DPP-1 inhibitors reduce neutrophilic inflammation,^459^ which prevents ECM remodeling and potentially supports regeneration downstream. Indeed, the DPP-1 inhibitor brensocatib inhibits the activity not only of DPP-1 itself but of neutrophil elastase, proteinase 3 and cathepsin G as well, probably contributing to its broad mode of action.^460^

##### d Neutrophil elastase inhibitors

Given the central role of neutrophil elastase in elastolysis, neutrophil elastase inhibitors have long been considered for the inhibition of emphysema development. The inhibitor FR901277 prevents elastase-induced emphysema development in rodents.^461^ Furthermore, ONO-5046, another neutrophil elastase inhibitor, was shown to prevent CS-induced lung injury in mice.^462^ Neutrophil elastase inhibitors are in clinical development for bronchiectasis. For example, the inhibitor BAY 85-8501 was found safe and showed target engagement in patients with bronchiectasis.^463^ Alvelestat (MPH966), an orally active neutrophil elastase inhibitor, is currently being evaluated for bronchiolitis obliterans syndrome (NCT02669251). Whether neutrophil elastase inhibitors are suitable for long-term treatment and inhibition of emphysema progression is unclear at this moment.

#### 4 Lymphotoxin-signaling inhibitors

The progression and severity of COPD are associated with increasing infiltration of the airways by both innate, predominantly neutrophils and macrophages, and adaptive immune cells (B and T lymphocytes). These form inducible bronchus-associated lymphoid tissue (iBALT), composed of B cells surrounded primarily by T cells.^464^, ^465^, ^466^ The number of iBALT structures increases in the lung with disease severity,^464^^,^^467^, ^468^, ^469^ and it was recently shown that they contribute to the pathogenesis of CS-induced COPD.^331^^,^^470^^,^^471^ Furthermore, unbiased transcriptomics data obtained from the lungs of patients with COPD revealed activated adaptive immune cell signatures strongly associated with the development of emphysema,^472^, ^473^, ^474^ which is accompanied by a significant correlation between emphysema severity and lymphoid organ formation.^469^^,^^474^

Crucial to our understanding of COPD pathogenesis and subsequent treatment is to elucidate the molecular mechanisms underlying how iBALT contributes to both tissue injury (emphysema) and the dysregulated repair and regenerative pathways observed in COPD. Many of the pathways responsible for the development and maintenance of iBALTs in general, mirror those responsible for lymphoid organogenesis during ontogeny.^475^^,^^476^ Crucial is the interaction between the lymphotoxin-*β* receptor (LT*β*R) on stromal organizer cells and membrane bound lymphotoxin, heterotrimeric complexes of the TNF superfamily members LT*α* and LT*β* (LT*α*1*β*2 or LT*α*2*β*1),^477^ expressed on the surface of CD45^+^-CD3^–^-CD4^+^-ROR*γ*t^+^ lymphoid tissue inducer cells.^478^, ^479^, ^480^ In lymphoid tissue formation during chronic inflammation, lymphocytes are capable of fulfilling the role of lymphoid tissue inducer cells.^481^, ^482^, ^483^, ^484^ Lymphotoxin signaling triggers expression of downstream chemokines such as CCL19, CCL21, and CXCL13 and cellular adhesion molecules such as VCAM1 and ICAM1, which attract and retain more hematopoietic cells.^479^ LT*β*R signaling activates the noncanonical NF-*κ*B pathway via NF-*κ*B-inducing kinase (NIK), which phosphorylates and activates IKK*α* homodimers. Activated IKK*α* then phosphorylates the NF-*κ*B precursor protein p100, leading to its partial proteasomal processing into p52. The resulting p52/RelB heterodimer translocates to the nucleus, where it drives transcription of target genes.^485^^,^^486^ Indeed, mice with a mutation in NIK (aly/aly mice), which lack noncanonical NF-*κ*B signaling, have no lymph nodes and present disorganized thymic and splenic architecture with impaired T cell mediated immunity,^487^, ^488^, ^489^ a phenotype also observed in mice deficient in lymphotoxin.^476^ Furthermore, blocking lymphotoxin signaling using a LT*β*R-Ig fusion protein,^490^ impairs the development and maintenance of conventional lymphoid tissue.^491^^,^^492^ Indeed, LT*β*R-Ig treatment, used to block LT signaling both prophylactically and therapeutically in the presence of CS, significantly reduced iBALT formation and resulted in more dispersed immune cell localization.^331^ Crucially, quantitative morphological analyses of lung tissue damage for airspace enlargement and alveolar surface density revealed that CS-induced emphysema was prevented by prophylactic LT*β*R-Ig treatment. Therapeutic treatment starting from 4 months, a time point at which airspace damage was already fully established in mice, led to full restoration of lung tissue, even in the continued presence of CS exposure.^331^

Interestingly, LT*β*R-induced stabilization of NIK is crucial for TNF-*α*-mediated cell death.^493^ NIK is required for the activation of caspase-8 by promoting the assembly of the RIP1/FADD/caspase-8 death complex.^493^ Consistent with this, increased AT2 cell death was observed in the lungs of both patients with COPD and mice chronically exposed to CS. In vitro*,* LT*β*R-signaling enhanced TNF induced AT2 cell death.^331^ Single-cell RNA-sequencing clearly revealed that CS strongly induced a positive regulation of NIK-dependent signaling in AT2 cells, which was significantly reduced upon LT*β*R-Ig treatment. In line, high levels of *Ltbr* mRNA expression on AT2 cells were found indicating that NIK dependent signaling in AT2 cells can be triggered by LT*β*R-activation. This demonstrates the novel concept that therapeutic inhibition of LT*β*R-signaling restores lung architecture from smoking induced-emphysema by reinitiated endogenous WNT/*β*-catenin-driven alveolar regeneration. Mechanistically, LT*β*R activation in progenitor AT2 cells suppresses WNT/*β*-catenin signaling via the noncanonical NF-*κ*B pathway, mediated by the NF-*κ*B-inducing kinase NIK.^331^ In primary AT2 cells and stable human and mouse cell lines treated with LT*β*R agonists, there was a clear downregulation of key WNT/*β*-catenin target genes *Axin2*, *Tcf4*, *Nkd1*, and *Lgr5*. Indeed, both *AXIN2* and *TCF4* expression were also suppressed in ex vivo human precision-cut lung slices stimulated with an LT*β*R agonist. Moreover, noncanonical NF-*κ*B signaling induced by the alternative LT*β*R ligand TNFSF14 reduced *β*-catenin levels in a murine AT2 cell line.^331^ Crucially, inhibition of GSK-3*β* ligand-independent *β*-catenin transcriptional reporter activity was prevented by LT*β*R activation, implying intracellular signal modification downstream of the *β*-catenin destruction complex. Indeed, proteasome inhibition with bortezomib prevented LT*β*R driven *β*-catenin degradation.^331^

Although direct LT-signaling inhibitors are lacking, small molecules targeting its downstream noncanonical NF-*κ*B signaling pathway have been in development for a number of years.^494^, ^495^, ^496^ Indeed, murine AT2 cells treated with the NIK inhibitor CMP137 prevented LT*β*R-signal induced degradation of *β*-catenin,^331^ suggesting NIK inhibition may be an alternative option for inducing lung regeneration in COPD. These series of experiments elegantly demonstrate that inhibition of LT*β*R-signaling in alveolar progenitors can both prevent epithelial cell death and activate WNT-induced regeneration promoted by *β*-catenin signaling.

#### 5 Cytokine receptors and cytokine-targeted antibodies

**Fig. 5:**
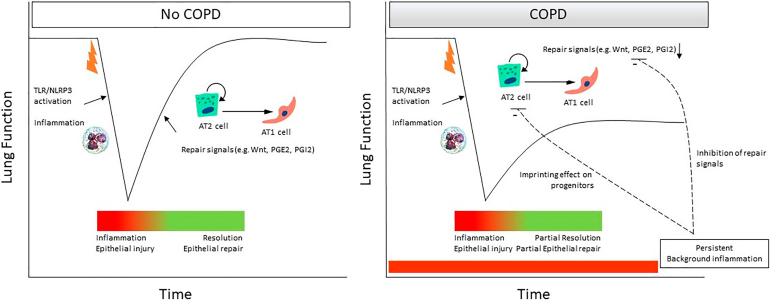
Interplay of persistent inflammation with defective repair. In otherwise healthy subjects without COPD (left panel), bacterial or viral infections will trigger NLRP3 inflammasome-dependent inflammation and a drop in lung function; this is followed by a resolution phase during which epithelial injury is repaired by signals such as WNT, or IL-1*β*. In COPD, there is a background of persistent inflammation that interferes with repair signals and/or negatively imprints on epithelial progenitors, leading to incomplete repair.

A defining feature of the COPD lung microenvironment is the presence of persistent inflammation characterized by the presence of neutrophils, macrophages, and lymphocytes, often present in iBALT structures as summarized in the previous section. These cells, and structural cells express several chemokines and cytokines to which the alveolar epithelial cell and its niche is continuously exposed.^497^ Exacerbations offer periods of enhanced exposure to these inflammatory stimuli. A key question from both a medical biology and pharmacology perspective is what the effect is of this inflammatory microenvironment on lung tissue repair. As summarized in section Proinflammatory cytokines, proinflammatory cytokines can have both detrimental effects and beneficial effects, dependent on the type of cytokine, and the duration of the exposure. It seems that acute inflammatory stimuli have beneficial effects on lung tissue repair, whereas chronic or persistent exposures have the reverse effect and counteract adequate lung tissue repair (Fig. 5).

These findings suggest that targeting specific cytokines or their receptors would be beneficial for lung tissue repair, particularly when directed at the background of persistent inflammation. Indeed, persistent exposure to IL-1*β* was associated with elevated expression of a range of CXC chemokines including CXCL1, CXCL5, and CXCL8. In this setting, inhibition of the common receptor CXCR2 using the drug reparixin effectively reversed the detrimental effects of IL-1*β* exposure on lung organoid growth in the exposure model where mesenchymal cells were pre-exposed to IL-1*β* before inclusion in the lung organoid assay.^88^ Thus, IL-1*β* can both enhance alveolar epithelial cell proliferation and differentiation via the NF-*κ*B pathway and, when dysregulated, impair mesenchymal support for epithelial growth.

Consistent with a detrimental role in emphysema development, mice deficient in IL1R1 or MyD88 develop reduced emphysema severity and ECM remodeling in response to elastase administration.^498^ Critically, inflammation in response to elastase was also reduced in these animals, suggesting that inflammatory responses, in addition to direct elastase effects, partially contribute to disease pathology. Likewise, mice deficient in IL-6 have reduced inflammatory cell counts in bronchoalveolar lavage fluid (BALF), and attenuated emphysema development in response to elastase exposure.^499^ The apparent contradiction with the previously mentioned protective roles for IL1R1 and IL-6 in lung repair^67^^,^^79^ is most likely explained by the difference in the model as these previously mentioned studies used influenza infection to model lung injury, which apparently produces different, even opposite, outcomes in comparison to the elastase model of emphysema.

Further support for targeted inhibition of proinflammatory cytokines comes from studies that used either genetically modified mice or function blocking antibodies against IL-17A. Mice deficient in IL-17A develop reduced levels of emphysema in response to elastase and have attenuated inflammation and cytokine levels in BALF. Levels of IL-17A were elevated already immediately (1 day) after elastase exposure in wild-type mice and remained elevated compared with nonexposed wild-type mice, suggesting that IL-17A may have contributed to the immediate development of inflammation in the elastase model.^500^ On the other hand, in a disease model in which weekly low-dose elastase exposure for 1 month was followed up with LPS exposure and/or respiratory syncytial virus (RSV) infection, an antibody targeting IL-17 was protective against inflammation and emphysema development even when administered after the elastase exposures. This suggests that IL-17 contributes to the perpetuation of either lung damage or a reduced tissue repair response after elastase exposure.^501^ This contention is further supported by the observation that antibodies targeting IL-23 or genetic ablation of IL-23 reduce the expression of T helper 17 cells and attenuate the development of emphysema in response to elastase in mice.^502^ While this does not indicate a direct role for IL-17 in tissue regeneration, there may be an indirect role because of inhibition of inflammation and subsequent epithelial injury.

Finally, the possibility of targeting type 2 inflammation is a strategy worth mentioning in the context of the recent developments on the protective effects of the IL-4R antibody dupilumab in patients with COPD.^503^ In this context, the observation that IL-4 plays a role in the development of inflammation and emphysema in response to elastase is of interest.^504^ Thus, interstitial macrophages produce MMP-12 in mice exposed to elastase and do so in an IL-4 dependent manner with basophils being the major source of IL-4 in the model. Mice deficient in IL-4 or mice with basophil-specific IL-4 deficiency fail to develop emphysema and have reduced expression of MMP-12 in response to elastase.^503^ Furthermore, the cysteinyl leukotriene receptor antagonist montelukast attenuates emphysema development in response to elastase in mice, and inhibits the ovalbumin-aggravated response in a model of combined allergen and elastase exposure.^505^ These findings raise the possibility that type 2 inflammation has an impact on emphysema development as well and that pharmacologically targeting this response may be beneficial.

#### 6 Other anti-inflammatory strategies

##### a Angiotensin pathway signaling

Angiotensin signaling is a complex biological pathway best known for the role of angiotensin II, which contributes to hypertension mediated by the AT_1_ receptor, and for which AT_1_ receptor blockers such as losartan and angiotensin converting enzyme (ACE) inhibitors such as captopril are used clinically.^506^ However, in addition to its role in the cardiovascular system, angiotensin contributes to pulmonary physiology and pathophysiology as well, both by signaling via the AT_1_ receptor and the AT_2_ receptor. The AT_2_ receptor, in contrast to AT_1_, generally exerts protective effects. Moreover, angiotensin II can be converted by ACE2 into angiotensin(1–7), which not only binds to the AT_2_ receptor but to the Mas receptor as well.^506^ Intriguingly, compound 21, a selective peptide agonist for the AT_2_ receptor, inhibits inflammation, p38 mitogen activated protein kinase (MAPK) pathway activation, lung function changes, and emphysema development in response to CS in mice.^507^ Moreover, an orally active formulation of angiotensin(1–7) was able to prevent emphysema development in response to elastase in mice, which was associated with repressed inflammation.^508^

##### b α7 Nicotinic receptor signaling

In contrast to the muscarinic receptor pathways, which are predominantly proinflammatory,^509^ *α*7 nicotinic receptor signaling has well established anti-inflammatory effects.^510^ *α*7 Nicotinic receptors are widely expressed on neurons and neuroendocrine cells, as well as on inflammatory cells such as macrophages.^511^ The selective *α*7 nicotinic receptor agonist PNU-282987 strongly inhibited the development of emphysema in response to elastase in mice, both as a preventive strategy and as a therapeutic strategy. This was associated with an equally strong inhibition of inflammation in response to elastase.^512^ PNU-282987 had similar protective effects on type 2 inflammation in animal models of allergen exposure.^513^

##### c Neuropeptide Y signaling

Neuropeptide Y (NPY) is a neuropeptide expressed by sympathetic neurons as well as by inflammatory cells and by epithelial cells, in particular neuroendocrine cells. Although there is limited literature available on NPY, reduced expression of the neuropeptide has been reported in the airways of patients with COPD.^514^ In addition, an interaction between the presence of NPY and emphysema development was reported in NPY−/− mice. Although NPY−/− mice have no emphysematous abnormalities themselves, the absence of NPY aggravates the inflammatory response and emphysema development in response to elastase exposure.^515^ It remains unknown whether NPY agonists could serve as therapeutic agents for emphysema.

##### d Receptor for advanced glycation end products signaling

Receptor for advanced glycation end products (RAGE) is highly expressed on AT1 cells and inflammatory cells, and its ligands (advanced glycation end products) are increasingly expressed in patients with COPD.^516^ Advanced glycation end products are considered damage associated molecular patterns with roles in linking tissue injury to inflammatory responses. In addition, they have negative effects on lung tissue repair as the antimicrobial protein LL-37 and HMGB1, both of which are endogenous RAGE ligands, reduce lung organoid forming capacity.^517^ In addition, they promote neutrophilic inflammation and emphysema development.^517^ Accordingly, inhibition of receptor for advanced glycation endproducts (RAGE) signaling using the drug N-benzyl-4-chloro--cyclohexylbenzamide (FPS-ZM1) prevented emphysema development, both in response to elastase and in response to LL-37 or HMGB1; it also reduced inflammatory cell infiltration, and suppresses damage associated molecular pattern-related signaling.^517^^,^^518^

### C Cell therapies

#### 1 Mesenchymal stromal cell-based therapy

The most widely described stem cell population used for cell-based strategies in regenerative medicine is the MSC. MSCs are multipotent stem cells that can be derived from various tissues, including bone marrow, adipose tissue, umbilical cord, and the lung.^519^^,^^520^ Because of the scarcity and limited numbers of adult human MSCs, human-induced pluripotent stem cells (iPSCs) are now increasingly used as a source of MSCs. iPSCs are derived by reprogramming of somatic cells from various tissues such as skin biopsies or urine samples, and can then be differentiated into iPSC-MSCs. Lung resident MSCs (LMSCs) are mesenchymal progenitors that replenish stromal cell populations, including lipofibroblasts, myofibroblasts, and smooth muscle cells. They reside within the microenvironment of alveolar epithelial cells and ECs and support site-specific proliferative and differentiation responses, secreting trophic factors such as FGF10, which is critical for embryonic lung development as well as adult lung homeostasis.^521^ In mice, a subpopulation of FGF10-expressing cells has been reported to represent resident MSCs that are able to self-renew.^522^^,^^523^ MSCs as well as (lipo)fibroblasts are an important source of FGF10. Of note, MSCs and fibroblasts are difficult to distinguish on the basis of their secretory, surface molecule or gene expression profiles in vitro. However, the regenerative potential of MSCs may be related to the higher proliferative capacity and lower susceptibility to undergo senescence upon expansion compared with fibroblasts.^524^ As described above, increasing numbers of studies show that communication between mesenchymal cells and the alveolar epithelium is crucial for alveologenesis, normal lung homeostatic maintenance and alveolar epithelial repair upon lung injury. A wide variety of growth factors secreted by MSCs have been implicated in mesenchymal-epithelial crosstalk during alveolar epithelial developmental and repair processes, including FGF10 and other FGFs, keratinocyte growth factor (KGF), WNT ligands, BMPs, and HGF. In addition, MSCs can secrete microRNAs and anti-inflammatory factors into the damaged microenvironment, suppressing allograft rejection and protecting against inflammation-induced injury. Rather than by their direct engraftment, MSCs are thought to exert their therapeutic effects mainly through their paracrine function. In addition to growth factors and (anti-)inflammatory mediators, their secretome consists of other soluble proteins and EVs, including exosomes. In preclinical models of acute lung injury (ALI) and acute respiratory distress syndrome (ARDS), administration of MSC’s secretome has been shown to improve survival, restore lung architecture, decrease fibrin deposition, and attenuate inflammation.^525^ The cell-free nature of secretome-based therapy offers advantages over live cell transplantation, including reduced risk of tumorigenicity and immune rejection.^526^ Bone marrow-derived MSC (BM-MSC)-derived EVs were shown to be safe in a clinical trial on the treatment ARDS in patients with COVID-19;^527^ however, secretome-based therapy offers new challenges such as limited duration of effects. Moreover, clinical translation is limited because of the heterogeneity in secretome composition, optimal dosing, delivery methods, and large-scale manufacturing challenges. EV treatment in COPD will be further discussed in section Extracellular vesicles.

Besides their paracrine effects, MSCs derived from adult tissues and iPSC-derived MSCs have been shown capable of mitochondrial transfer, reducing lung tissue damage upon smoking and oxidative stress and protecting against mitochondrial dysfunction in animal models.^528^, ^529^, ^530^ Accordingly, various animal studies have shown that MSCs are beneficial in lung disease, with the ability to ameliorate emphysematous lesions when administered either prophylactically or therapeutically.^531^

MSCs from adult tissues have been extensively and successfully used in clinical trials aiming at dampening immune reactions and enhancing tissue regeneration, such as for the treatment of Graft-versus-Host Disease,^532^ Crohn’s disease,^533^ cardiac ischemia, and after solid organ transplantation.^534^ However, the clinical application of MSCs in lung disease is still in its infancy and evidence for beneficial clinical effects of transplanted MSCs on lung function is limited. In the first clinical trial in COPD, BM-MSCs were delivered intravenously. The treatment was well tolerated and resulted in a significant, early reduction in systemic C-reactive protein levels, but without effect on lung function.^535^ In a post hoc analysis, the treatment significantly improved lung function in those patients with high C-reactive protein levels, indicating clinical benefit through anti-inflammatory effects.^536^ In another clinical trial in patients with severe emphysema undergoing lung volume reduction, treatment with BM-MSCs confirmed that treatment was safe. Moreover, this study reported increased CD31 expression, suggesting responsiveness of microvascular ECs, yet again without a beneficial effect on lung function.^537^

Because of the complex architecture of the lung and the extensive alveolar destruction in emphysema, the challenge of achieving lung tissue repair is considerable, and important questions on the optimal route, dosage, frequency of treatment, and source of MSCs remain to be answered. Various animal studies have compared the use of MSCs from different sources. In an elastase-based mouse model, comparison of LMSCs to BM-MSCs showed higher retention of LMSCs in the lungs, which was accompanied by higher ICAM-1, integrin-*α*2, and PDGFR*α* expression, and may thus relate to higher ability of MSCs to adhere to ECs and migrate into the lung tissue.^538^ LMSCs and BM-MSCs showed similar growth factor receptor and inflammatory mediator expression profiles, and both cell types reduced elastase-induced lung damage. In another study comparing the effects of intravenous and intratracheal installation of adipose-derived MSCs (AD-MSCs), BM-MSCs, and LMSCs in a mouse model, cells from all sources reduced elastase-induced mean linear intercept, neutrophil infiltration and alveolar epithelial and EC damage, and increased elastic fiber content, independent of administration route. However, only BM-MSCs displayed beneficial systemic effects, whereas AD-MSCs and LMSCs showed a more significant reduction in the fractional area of alveolar collapse than BM-MSCs.^539^ This may be linked to the immunomodulatory/anti-inflammatory profile of BM-MSCs,^540^ which translated into reduced systemic cytokines^541^ and protection not only of the lung but also in extrapulmonary tissues in models of acute lung injury upon their administration in a preclinical model.^542^ In contrast, the more localized effects of AD-MSCs and LMSCs may be attributed to their tissue-specific gene expression profiles. LMSCs, in particular, express higher levels of growth factors such as FGF10 and HGF.^164^

For their application in the clinic, one of the hurdles that needs to be taken, irrespective of the cell source and route of delivery, is the short retention time of MSCs in the lung. Even though intravenously administered MSCs initially become trapped in pulmonary capillaries, they are cleared within a few days.^543^ Although retention was initially higher in mice with elastase-induced emphysema,^543^ a hostile lung microenvironment in COPD, with high levels of oxidative stress, inflammation and loss of ECM may significantly impact on the attachment and survival of MSCs.^544^ Furthermore, administered MSCs may be cleared rapidly by phagocytosing immune cells. This needs to be taken into account when considering the most appropriate dosing frequency and route of administration. A potential solution is the use of a delivery scaffold, such as microgel encapsulation to protect the cells and improve their retention time.^545^ Insight into factors in the MSC secretome that are crucial for alveolar repair will further guide the design of such a delivery scaffold and/or preconditioning strategies of the cells. When the strategy is aimed at the use of autologous MSCs, it is important to consider abnormalities in gene expression profiles and pathways of MSCs derived from patients with COPD. COPD-derived LMSCs express lower levels of HGF and FGF10.^546^ Strikingly, even more differences in gene expression were observed between BM-MSCs and AD-MSCs from patients with COPD and controls.^164^ One of the pathways that may be dysregulated in MSCs from patients with COPD is the Hedgehog (Hh)-Glioma-associated oncogene 1 axis, which is regulated by COPD susceptibility gene *HHIP*,^547^ encoding Hh interacting protein. Glioma-associated oncogene 1 was found higher expressed in fibroblasts, which share mesenchymal stem cell features with MSCs, from smokers and patients with COPD (PMID: 25815884). In animal models, loss of HHIP expression resulted in activation of Hh signaling in fibroblasts, promoting emphysematous manifestations^548^ and potentiating the release of IL-7 by Gli^+^ fibroblasts.^549^ Moreover, Gli1^+^ MSCs were shown to contribute to abnormal alveolar differentiation upon injury.^550^ Animal models provide evidence that the HHIP/Hh axis is a reachable target and pharmacological modulation of Hh pathways may thus represent an opportunity to enhance lung tissue repair.^551^

Besides the dysregulation of regenerative pathways in COPD, another limitation of autologous MSC is in vitro expansion of MSCs, which can lead to the induction of replicative senescence, and the induction of senescence is accompanied by lower levels of FGF10.^524^ Of note, when compared with fibroblasts from lung tissue of the same donors, MSCs showed lower sensitivity toward both stress-induced and replicative senescence, indicating that MSCs are less likely to senesce upon in vitro expansion.^524^ Additionally, the use of iPSC-derived MSCs may overcome this issue, but this comes with the risk of tumorigenesis. Here, microencapsulation to prevent proliferation may be an option to explore for future strategies. Together, with continued insight into the action of (iPSC-induced) MSCs and how to overcome their limitations, the application of these cells holds significant promise in regenerative medicine in the lung.

#### 2 Organoid and induced pluripotent stem cell-induced epithelial cell therapy

In COPD the epithelial barrier is compromised due to disrupted tight and adherens junctions, leading to increased permeability and susceptibility to pathogens. In the upper airways, the local stem cells, called basal cells, exhibit dysregulated differentiation, contributing to ciliary dysfunction and mucus overproduction. Similarly in the distal lung, the alveolar progenitor cells (AT2) show impaired differentiation into AT1 cells, compromising gas exchange.^552^ Aggravating risk factors such as cigarette smoke and other pollutants are thought to impair regeneration by increasing oxidative stress, leading to senescence, sustained tissue damage and remodeling.^553^^,^^554^ However other mechanisms for failed regeneration likely also contribute and our understanding remains limited. Recent advances in regenerative medicine, particularly involving iPSCs, lung organoids, and adult stem cell transplantation, are opening new avenues for both disease modeling and therapeutic intervention. These innovative approaches aim at providing the damaged tissue with an alternative source of progenitor cells required for lung repair and integrity.

##### a Transplantation of induced pluripotent stem cell-derived lung cells

Several recent studies have demonstrated that when iPSC-derived basal cells are transplanted into injured airways of immunocompromised mice, they can engraft, self-renew, and contribute to the long-term regeneration of functional airway epithelium.^555^, ^556^, ^557^ In addition to basal cells, other lung-resident epithelial stem and progenitor populations have been derived from iPSCs and tested in preclinical models. For example, lung tip progenitor cells, characterized by expression of transcription factors such as SOX9 and ID2, have been generated and successfully engrafted into the distal lung of mice after naphthalene-induced injury, supporting localized repair.^558^ Similarly, studies have reported the generation and engraftment of AT2 from patient-specific iPSCs, which are critical for surfactant production and alveolar homeostasis. These iPSC-derived AT2s have been shown to survive and integrate into the alveolar niche in vivo, contributing to alveolar regeneration after injury.^559^^,^^560^ These findings suggest that multiple kinds of lung progenitors could be employed to repair or replace damaged epithelial tissues in patients with COPD, especially in scenarios where chronic inflammation and repeated injury disrupt epithelial integrity and repair capacity.

##### b Transplant of lung primary cells and their derivatives

Whereas iPSC-derived cell therapies offer a customizable and patient-specific approach, concerns about immune system avoidance, tumorigenic potential, and genomic instability remain significant barriers to clinical translation. As an alternative, transplantation of endogenous airway and alveolar progenitor cells is emerging as a promising and potentially safer strategy for long-term lung regeneration.

Basal cells have demonstrated stable engraftment and long-term regenerative capacity in preclinical models. In a key study,^556^ human basal cells were expanded in ex vivo culture and transplanted into the airways of immunocompromised mice, where they engrafted successfully, maintained basal cell identity, and differentiated into multiple airway epithelial lineages over time. Similar studies demonstrated efficient expansion of human airway basal cells and their successful engraftment into bleomycin, elastase, and LPS mouse lung injury models.^561^, ^562^, ^563^ These findings suggest that isolated and expanded adult basal cells could support durable epithelial repair after transplantation.

Beyond the proximal airways, efforts have also focused on regenerating distal lung structures, particularly the alveoli. Lung epithelial organoids derived from adult alveolar progenitor cells—primarily AT2 cells—have shown significant regenerative potential in preclinical models. Organoid-derived AT2 cells have been transplanted into bleomycin injured immunocompromised mouse lungs. These cells were engrafted in the alveolar regions and contributed to epithelial repair. Importantly, recipient mice showed decreased fibrosis and immune infiltration compared with control mice, indicating the therapeutic potential of alveolar stem cell delivery.^564^^,^^565^ A similar study showed AT2 organoid transplant in flu-injured mice aids in oxygen saturation recovery and lung repair,^552^ and another demonstrated whole lung cell transplantation can aid in the resolution of a pulmonary fibrosis bleomycin mouse model.^566^

Together, these studies support the growing consensus that transplant of airway and alveolar progenitor cells represent a promising approach to restore the lungs regenerative capacity in chronic lung diseases impacting epithelial cells such as COPD.

##### c Autologous transplantation in human: P63^+^ lung progenitor cell transplantation

A step toward clinical translation was recently reported with the first-in-human trial investigating the autologous transplantation of P63^+^ airway basal progenitor cells in patients with COPD.^567^^,^^568^ These cells were isolated via bronchoscopic brushing and expanded ex vivo in a pharmaceutical grade culture system before patient transplantation via bronchoscopy. Patients who received transplanted cells showed improved diffusing capacity for carbon monoxide, 6-minute walk distance, and patient-reported respiratory symptoms, whereas control patients had continued lung function decline. This on-going clinical trial underscores the potential of patient cell transplantation for therapeutic benefit and epithelial regeneration in patients with COPD, although consideration must be taken for possible genomic instability in transplanted cells from ex vivo culture.

##### d Challenges and future directions

Despite these promising developments, several challenges remain. Efficient differentiation of iPSCs into functional lung epithelial subtypes, the time required to derive iPSCs from each patient, and long-term engraftment stability are critical barriers to overcome. Perhaps one of the most significant challenges of iPSC-derived cells are their lack of maturation compared with cells from adult tissues; further development of culturing platforms may aide this deficiency in the future.

It is notable that the human lung is significantly larger than murine lungs, which have been used for the proof-of-principle transplantation and engraftment studies; a major question that remains is what is the number of cells required for engraftment to have a therapeutic benefit for patients with COPD? The number of cells needed may be proportional to the size difference, however it is possible that engraftment of a portion of the lungs will be sufficient, and overall likely is highly dependent on the severity of their disease. To mimic lung injury and to promote engraftment, all developed methods to date require preconditioning of the recipient lung with damaging agents, akin to the ablation of the bone marrow that precedes transplantation of hematopoietic stem cells. However, it is not clear how to utilize these treatments in patients with lung disease. Furthermore, as we learn more about the lung microenvironment in repair and disease, new transplant approaches need to consider the role of endothelial and mesenchymal cells as both cotransplant adjuvant as well as in engraftment efficiency. Finally, immune barriers must be addressed in clinical settings to prevent rejection and allow for enough cell engraftment to effectively restore lung function.

iPSC and organoid technologies are rapidly advancing from experimental models to potential clinical interventions for COPD. As demonstrated by recent studies, regenerative epithelial cell therapies have the potential to restore epithelial integrity and regenerative capacity in patients with COPD. Continued interdisciplinary research and carefully designed clinical trials will be essential to investigate therapeutic potential of these novel approaches.

#### 3 Endothelial cell therapy in chronic obstructive pulmonary disease

Recent investigations have uncovered the functional role of ECs in lung regeneration, suggesting targeting pulmonary ECs is an effective intervention to restore functional gas exchange in respiratory disease. Currently, corneal EC therapy is widely conducted in clinical trials,^569^, ^570^, ^571^ but limited information is available for respiratory diseases. Intravenous infusion of autologous EPCs is feasible and safe and may be beneficial to patients with idiopathic pulmonary arterial hypertension.^572^ The delivery of EPCs overexpressing endothelial nitric oxide synthase is also tolerated hemodynamically in patients with pulmonary arterial hypertension^573^ with a trend toward improvement in total pulmonary resistance during a short delivery period, although one severe adverse event occurred after discharge.

##### a Therapeutic potential of endothelial progenitor cells therapy in chronic obstructive pulmonary disease

COPD is significantly associated with endothelial dysfunction contributing to both airway remodeling and alveolar destruction.^574^, ^575^, ^576^ Pulmonary ECs lining along the arteries, veins and capillaries mediate the interactions between blood and lung tissue, which is vital for angiogenesis, regulation of blood flow, vascular permeability, wound healing, and inflammation.^577^^,^^578^ The primary function of the respiratory system is gas exchange where the functional compartment responsible for it is the alveolus that is comprised of multiple epithelial, endothelial, and mesenchymal cell subtypes.

Though there is currently no EC therapy trial conducted in patients with COPD, EC therapy has been beneficial in animal models of elastase-induced and CS-induced COPD.^138^^,^^579^^,^^580^ Specifically, cotransplantation of tissue-resident alveolar macrophages and EPCs improves the efficacy of EPCs therapy in hyperoxia-injured lungs.^580^ To identify the regenerative potential of EC therapy in emphysema, GFP-labeled E4ORF1-transduced lung ECs were intravenously delivered at days 7 and 14 after elastase treatment. This intervention significantly reduced parenchymal destruction and decreased mean cord length.^138^ Delivery of human iPSC-derived distal ECs together with pneumocytes in an elastase-induced rat emphysema model via intratracheal injection led to about 15% engraftment in the host alveoli and these cells integrated to form vascularized alveoli together with host cells.^579^ In animal models of COPD induced by cigarette smoke exposure, systemic administration of EPCs has been shown to alleviate multiorgan senescence and modulate disease-associated pathways, including the USP7/p300 axis, where USP7 stabilizes the coactivator p300 involved in gene regulation and cell differentiation. However, these interventions have not reversed established tissue morphological changes.^581^ Taken together, these studies indicate that EPCs possess significant value for restoration of alveolar destruction associated with chronic lung diseases in different animal models, and their regenerative potential can be achieved both via intravenous and intratracheal delivery.

#### 4 Platelet-rich plasma therapy for chronic obstructive pulmonary disease

Platelet-rich plasma (PRP) therapy has been postulated as a potential adjunct therapy for COPD, with the notion that it could slow the progression of the disease and improve patient quality of life. PRP is an autologous product, derived from the patient’s own blood; a number of methods have been developed to extract the desired components from whole blood in a short period of time (∼15 minutes) for administration back to the patient.^582^ Previous studies have demonstrated that positive therapeutic outcomes after musculoskeletal injury can be achieved with doses around 3.5 billion platelets per administration, with cumulative doses reaching up to 10 to 12 billion platelets in multiple dosing strategies.^583^ PRP contains an array of biological factors that have the potential to modulate inflammation and remodeling processes in disease tissues.

PRP has the capability to modulate chronic disease pathology via a number of mechanisms. PRP has been found to reduce tissue inflammation, a primary characteristic of the COPD lung. In the context of musculoskeletal injuries, PRP administration was able to reduce the levels of proinflammatory cytokines (IL-17A, IL-1*β*, TNF-*α*, IL-6, and IFN-*γ*), increase the expression of angiogenic factors (HGF, VEGF, PDGF, IGF-1, and TGF-*β*), and improve joint structure assessed by magnetic resonance imaging.^584^ In the context of lung disease, PRP has been shown to reduce IL-1*β* levels in patients with COVID-19.^585^

The administration of PRP to damaged tissue may also have regenerative potential. Both nebulized and nonnebulized PRP promoted fibroblast proliferation in vitro compared with controls.^586^ In the lung, PRP has been found to increase lung vascularity and alveolar regeneration in mice after right lung pneumonectomy.^587^ Mechanistically, this was found to occur through a WNT-dependent pathway involving LRP5 phosphorylation and activation of the Tie2 receptor in ECs. PRP treatment resulted in accelerated EC sprouting in vitro and improved lung tissue regeneration in mice after unilateral pneumonectomy. Platelet-derived factors, in particular CXCL12 (SDF-1), have also been shown to prime the pulmonary capillary vascular niche and promote alveolar regeneration. After left lobe pneumonectomy in mice, platelet-derived CXCL12 stimulation of the CXCR4/7-Akt pathway in pulmonary capillary ECs induced metalloproteinase MMP14 expression and caused the release of HB-EGF, thereby stimulating the proliferation of alveolar epithelial cells driving neo-alveolarization.^588^

Recently, a clinical case series described the use of submucosal injections of autologous PRP in 3 patients with tracheobronchial fistulae; this treatment was successful in all patients with no treatment-related complications, suggesting the potential of PRP to promote localized tissue repair.^589^ In patients with COPD, a number of small cohort studies have been performed to assess the ability of PRP to improve lung function and quality of life. Although some of these studies have reported an increase in FEV_1_ and symptom scores, due to the lack of data on patient COPD severity and comorbidities, as well as incomplete reporting of lung function data,^590^ it remains to be seen if PRP is a viable adjunct treatment strategy for COPD. Large-scale double-blinded and controlled studies are eagerly anticipated to explore this further.

Currently, it is thought that much of the regenerative effect of PRP therapy for tissue repair is mediated by EVs released from activated platelets.^591^ Although the mechanistic basis for the regenerative potential of PRP-ECs is an area of continuing investigation, recent studies focusing on the cargo of platelet-derived EVs have revealed that target cell pyroptosis could be inhibited via EV-delivered long noncoding RNAs and microRNAs interfering with the SIRT1 axis.^592^ The advantages of EV-based treatments, including lower immunogenicity, improved tissue penetration, and the ability to deliver bioactive molecules directly to target cells certainly make EV-based therapies attractive for tissue regeneration, but this enthusiasm must be tempered by the current lack of standardization regarding the cell type of EV origin and EV dosing strategies.

### D Extracellular vesicles

As mentioned previously, MSCs have emerged as a promising option for regenerative therapies, particularly in the treatment of respiratory diseases such as COPD.^521^^,^^593^^,^^594^ MSCs have shown great potential in preclinical studies, where they have demonstrated the ability to reduce inflammation, modulate immune responses, promote angiogenesis, and support tissue repair through their paracrine effects.^521^^,^^593^^,^^594^ However, despite these promising preclinical results, clinical trials with MSCs have often yielded disappointing outcomes. One of the challenges has been the rapid clearance of MSCs by the body’s immune system, particularly by macrophages, which limits their effectiveness in the targeted lung tissue. Additionally, the complexity and high costs associated with MSC-based therapies, including the need for careful formulation, delivery, and sometimes surgical procedures, have further complicated their clinical application.^593^, ^594^, ^595^ An alternative approach is to induce or enhance lung repair using biologically active factors from the secretome of mesenchymal cells, which could be applied at an early stage of the disease and on a larger scale.^594^ MSCs and other cell types within the alveolar niche release EVs, which locally influence neighboring cells. Initially, EVs were thought to function primarily as a mechanism for cellular waste disposal.^596^ However, subsequent research has revealed their critical roles in intercellular communication and regulation of various biological processes, offering promising applications for disease diagnosis and treatment.

EVs are heterogeneous, cell-secreted particles enclosed by a phospholipid bilayer membrane.^597^ The 2 most studied EV subtypes, large vesicles (microvesicles) and small vesicles (exosomes), are classified based on their size and biogenesis. Microvesicles, which range from 0.1 to 1–2 *μ*M in diameter, bud directly from the plasma membrane. In contrast, exosomes, typically 30 to 150 nm in diameter, originate from endosomal multivesicular bodies and are released when these structures fuse with the plasma membrane.^598^ Due to their overlapping size, density, and protein markers, isolating pure vesicle populations remains challenging. In line with the Minimal Information for Studies of Extracellular Vesicles guidelines, the term EVs will be used generically in this review to describe all lipid-bilayer-delimited particles naturally released from cells that lack replication ability.^599^ EVs carry various bioactive molecules, including proteins, lipids, and genetic material (mRNA and microRNA). Once released, they interact with target cells via ligand-receptor interactions or are internalized through phagocytosis, endocytosis, or direct membrane fusion.^600^ The activation of specific membrane receptors on target cells triggers signaling cascades that modulate biological processes, influencing cell behavior and tissue homeostasis.

EVs have recently attracted significant attention as potential regenerative agents, with an increasing body of research exploring their therapeutic role in tissue repair and regeneration. In this context, EVs from a variety of cellular sources have been investigated as potential treatments for COPD, further underscoring their relevance in regenerative pharmacology. Among these, MSCs have been the most widely studied source, with EVs derived from bone marrow, umbilical cord, and adipose tissue being evaluated in several preclinical models, such as mouse models of emphysema (Table 1).^541^^,^^601^, ^602^, ^603^, ^604^ Although bone marrow- and umbilical cord-derived MSC EVs have demonstrated anti-inflammatory effects and protection against emphysema progression, adipose-derived MSC EVs failed to improve lung function, highlighting the variability in MSC-derived EV efficacy depending on their cellular origin.^541^^,^^601^, ^602^, ^603^, ^604^ Beyond MSCs, other cell types have also shown promise as EV sources. For instance, we demonstrated that alveolar lung fibroblast-derived EVs improved lung function and reduced elastase-induced lung injury, suggesting that resident lung cells may play an important role in alveolar repair.^149^ Positioned in situ near alveolar progenitor cells, these fibroblasts may facilitate localized EV signaling, directly supporting progenitor cell survival and regeneration.^149^ Similarly, platelet-derived EVs have shown protective effects in a CS-induced COPD model, whereas genetically modified HEK293T cell-derived EVs (WNT-3A-transfected) enhanced alveolar repair and lung function recovery in an elastase-induced emphysema model.^65^^,^^605^ These findings suggest that EVs derived from non-MSC sources may provide alternative strategies for lung regeneration, particularly if their cargo can be engineered or optimized for targeted therapeutic effects.

**Table 1:** EVs as mediators of tissue repair and regeneration in COPD

EV Source	Isolation Method	Concentration/Dose/Time Frame	Preclinical COPD Model Employed	Route of Administration	Effects Observed	Mechanism of Action	Reference
Human lung fibroblasts (MRC5)	Ultrafiltration and SEC	1.5 × 10^9^ and 4.5 × 10^9^ EVs, 5 doses, 8 days	Elastase-induced lung injury in mice	Intratracheal	Improved lung function and reduced lung injury	Not mentioned	van der Koog et al^149^
Human umbilical cord MSCs	Ultracentrifugation	EVs isolated from 2.5 × 10^6^ cells, once	12-week CS model in rats	Intratracheal	Reduced inflammation and decreased emphysema	Modulation of the NF-*κ*B pathway	Ridzuan et al^601^
Transfected (WNT-3A) HEK293T cells	Ultracentrifugation	2 × 10^9^ EVs, 4 doses, 14 days	Elastase-induced lung injury in mice	Intravenous	Improved lung function and reduced lung injury	WNT-3A signaling	Gao et al^65^
Healthy and emphysematous MSCs	Ultracentrifugation	EVs isolated from 1 × 10^6^ cells, once	Elastase-induced lung injury in mice	Intravenous	Healthy EVs reduced lung injury and inflammation	Reduction of proinflammatory cytokines (IL-1*β*, TGF-*β*, and IL-10)	Antunes et al^602^
Human adipose-derived stem cells	Ultracentrifugation	Dose unclear (based on protein concentration), once, 14 days	Elastase-induced lung injury in mice	Intratracheal	No improvement on lung injury	FGF2 signaling	Kim et al^604^
Human platelets	Not mentioned	2.5 × 10^10^ and 5.0 × 10^10^ EVs/mL, 12 doses	16-week CS model in mice	Nebulized	Improved lung function and reduced lung injury	Reduced NF-*κ*B activation and apoptosis	Xuan et al^605^
Human bone marrow-derived MSCs	Ultracentrifugation	EVs isolated from 4 × 10^6^ cells, once	16-week CS model in mice	Intratracheal and intravenous	Intratracheal administration reduced lung injury, intravenous not	Not mentioned	Jin et al^603^
Human bone marrow-derived MSCs	Size exclusion chromatography and affinity chromatography	0.5 × 10^8^, 1.0 × 10^8^, or 1.5 × 10^8^ EVs/kg, 5 doses	4-week CS model + intratracheal LPS in rats	Nebulized and intravenous	Improved lung function, reduced inflammation. Most pronounced with lowest dose, no effect intravenous	Suppression of the WNT/*β*-catenin signaling pathway	Wang et al^541^

SEC, size exclusion chromatography.

In addition to the wide range of cellular sources under investigation, the dosing strategies employed in EV-based therapies for COPD vary substantially across studies (see Table 1). Reported doses range from approximately 0.5 × 10^8^ to 5.0 × 10^10^ EVs per dose, with differences not only in the absolute quantity but also in the number of administrations used. Most preclinical studies investigated a single EV dose without assessing dose-response relationships, thereby limiting our understanding of the optimal therapeutic window. Furthermore, it is important to acknowledge that reported EV doses are typically quantified based on total particle number, which is an indirect measurement and potentially confounded by non-EV contaminants.^598^ This lack of a biologically meaningful and standardized quantification method complicates dose comparison across studies and presents a significant challenge for therapeutic standardization. Several studies report EV yields relative to the number of cultured donor cells rather than an absolute EV quantity, which introduces significant variability due to differences in culture conditions and isolation protocols. Compounding this issue is the use of diverse isolation methods across studies, which can affect the purity and biological composition of the final EV preparations.^606^ Most notably, differential ultracentrifugation remains the most widely applied method, yet it is known to coisolate soluble proteins and other non-EV components unless followed by additional purification steps.^607^^,^^608^ These impurities may contribute to biological effects that are erroneously attributed to EVs themselves. Indeed, recent evidence suggests that non-EV components of conditioned media can account for a substantial portion of the observed regenerative activity.^608^ Collectively, these challenges underscore the urgent need for standardized EV quantification, purification, and reporting practices to improve reproducibility and facilitate meaningful comparisons across studies.^599^

The route of administration is a critical factor in the therapeutic application of EVs for COPD. Most preclinical studies have used either intratracheal or intravenous delivery of EVs (Table 1). In many cases, both administration routes yielded therapeutic effects. However, direct comparisons have highlighted differences in efficacy. For instance, Jin and colleagues demonstrated that although intratracheal administration of EVs significantly reduced lung injury, the same EVs administered intravenously failed to show efficacy.^603^ These findings suggest that local pulmonary delivery may be more effective than systemic approaches for targeting lung tissue. Despite the promising results of intratracheal administration in rodents, this method is not feasible in clinical practice. An alternative strategy is nebulization, which allows noninvasive aerosolized delivery of EVs directly to the lung. Several studies have demonstrated the feasibility and therapeutic benefit of nebulized EVs. In a recent study, nebulized EVs improved lung function and reduced inflammation, whereas intravenous administration had no observable effect.^541^ Similarly, nebulized EV were found to reduce lung injury and enhance lung function in other models of lung damage.^605^^,^^609^

Although nebulization enables the delivery of relatively high doses of active pharmaceutical ingredients directly to the lungs, it is also associated with several limitations. These include limited delivery efficiency, considerable interpatient variability, prolonged administration times, and challenges in achieving consistent dosing.^610^^,^^611^ Furthermore, many biopharmaceuticals, including EVs, exhibit instability in aqueous solution or suspension, which complicates long-term storage and distribution without appropriate formulation strategies.^610^^,^^611^ Dry powder inhalers represent a promising alternative for pulmonary delivery of EVs, as dry powder formulations can significantly enhance the storage stability of sensitive biologics.^610^, ^611^, ^612^ Two principal strategies have been explored for formulating EVs as dry powders: freeze drying and spray-drying. Freeze drying is a well established technique used to preserve biological products by removing water through sublimation.^613^, ^614^, ^615^ Several studies have demonstrated that EVs retain their physical properties and biological activity after freeze drying.^616^, ^617^, ^618^, ^619^ Moreover, distribution studies in murine and nonhuman primate models have shown that inhaled, freeze-dried EVs carrying GFP mRNA successfully localize to both bronchioles and parenchyma, resulting in functional protein expression within lung cells.^620^ These findings indicate that EVs can reach their cellular targets and deliver biologically active cargo via the inhaled route. Spray-drying offers an alternative, single-step method for producing respirable dry powders.^610^^,^^614^^,^^615^ This process typically yields spherical and homogeneously sized particles with favorable aerodynamic flow properties.^611^ Although research in this area is still limited, our work shows that lung fibroblast-derived EVs can be successfully spray-dried using excipients such as inulin and leucine, as stabilizer and dispersibility enhancer, respectively.^616^ The resulting powder retained their structural integrity and biological activity for at least 12 weeks and exhibited properties suitable for deep lung deposition using a dry powder inhalers.^616^ Although these findings are currently based on in vitro data, they provide a promising foundation for future in vivo studies aimed at confirming the therapeutic efficacy of spray-dried EV formulations delivered via inhalation.

Although preclinical studies offer compelling evidence for the regenerative potential of EVs in COPD, their clinical translation is still in its infancy. To date, only a limited number of clinical studies have explored the therapeutic use of EVs in respiratory disease, and in the context of COPD, only 1 published study has been identified. In this study, patients received weekly inhalations of Exo-d-MAPPS, a formulation containing MSC-derived EVs supplemented with high concentrations of immunomodulatory factors.^621^ Thirty patients with COPD were treated once per week for 3 weeks with 0.5 mL of Exo-d-MAPPS via inhalation.^621^ All treated patients exhibited improvements in pulmonary function and quality of life, as evidenced by increases in FEV_1_, peak expiratory flow, 6-minute walking distance, and reductions in Clinical COPD Questionnaire scores.^621^ Although it remains unclear which components of the formulation drove these effects, the treatment was well tolerated, demonstrating the feasibility and safety of inhaled EV-based therapies in a clinical COPD population. This study offers a valuable first benchmark for future trials, particularly regarding the use of inhalation as a delivery method.

However, several translational challenges must still be addressed. Scalable, GMP-compliant EV production systems are needed to ensure consistent yield and purity, yet current manufacturing practices often result in variable product composition and potential contamination.^621^, ^622^, ^623^, ^624^ Most efforts still rely on multilayered cell factories, though advances such as hollow-fiber bioreactors, microcarrier-based stirred tanks, and 3-dimensional spheroid cultures show promise for clinical upscaling.^622^^,^^625^, ^626^, ^627^ Beyond production, EV therapies demand rigorous quality control, including batch-to-batch consistency, validated potency assays, and comprehensive molecular characterization.^625^^,^^628^ Finally, dedicated regulatory frameworks for EV-based products have not yet been established, requiring concerted efforts from regulatory agencies, scientific societies, and industry stakeholders to guide safe and effective clinical implementation.^598^

### E Cell-derived therapeutic proteins

**Fig. 6:**
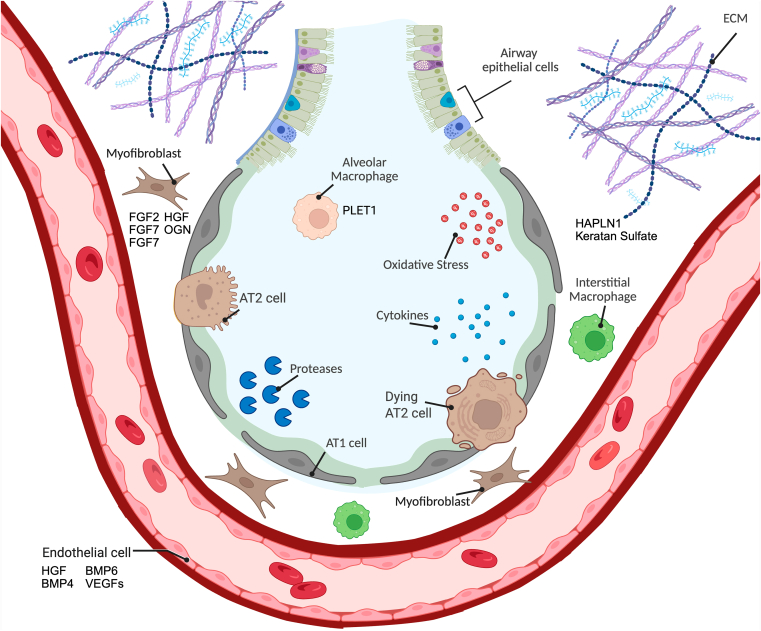
Mining the niche for regenerative therapeutics. The alveolar niche has well established roles in guiding epithelial repair and regeneration, and many secreted factors derived from lung fibroblasts, ECs and immune cells have been identified. Some of these have been used as drugs or as leads for drug development. Created in BioRender (https://BioRender.com/o0uhysv) by Van der Koog, L.

In view of the well established roles of the alveolar niche in guiding epithelial repair and regeneration, major research efforts have been directed at identifying the secreted factors derived from lung fibroblasts, ECs and immune cells in an attempt to utilize these as drugs or as leads for drug development. This section will summarize the niche-derived proteins with potential therapeutic value (Fig. 6).

#### 1 Fibroblast growth factors

FGFs represent a large family of growth factors mainly expressed by MSCs, which signal to FGF receptors (FGFRs), for which 4 different receptor tyrosine kinase subtypes (FGFR1–4) exist. The ligand-receptor interactions between most FGFs (eg, FGF10) and FGFR involve FGF binding to heparan sulphate, which maintains FGFs localized to the tissue of origin, ensuring a paracrine function.^629^ On the other hand, endocrine FGFs that circulate in the bloodstream such as FGF23, require binding to *α*-Klotho to activate FGFR signaling.^630^ The degree of heparan sulphate binding of paracrine FGFs is a key determinant of their biological action radius. For example, FGF10, which binds to heparan sulphate with higher affinity than FGF7 does, has a more restricted action radius than FGF7, which is key to the differential roles of FGF10 and FGF7 in epithelial gland budding (FGF10) versus branching (FGF7) during development.^631^ Downstream of the FGF–FGFR interaction is the tyrosine kinase-dependent activation of proliferation and survival pathways such as PI3K, p42/p44 MAPK, and FAK.^629^

In lung fibroblasts, the main FGFs expressed are FGF2, FGF7, and FGF10 and these FGF family members are also the best studied in the context of lung tissue repair and regeneration.^82^^,^^632^ On the other hand, FGFR1 and FGFR2 are the main receptors expressed, for which FGFR1 is mainly expressed by lung fibroblasts and FGFR2 mainly by epithelial cells,^82^^,^^632^ underscoring the role of FGF/FGFR signaling in both autocrine fibroblast functions and in mesenchymal to epithelial cell signaling in the lung. The expression of FGF7 is upregulated in lung fibroblasts exposed to a cocktail of cytokines relevant to the COPD exacerbation,^82^ whereas TGF-*β* reduces the expression of FGF7 and FGF10, while increasing the expression of FGF2.^422^ The expression of FGF1, FGF2, and FGFR1 is increased in the airways of patients with COPD,^633^ whereas the expression of FGF7 and FGF10 by lung fibroblasts is decreased in COPD.^634^ In addition, an interaction with single nucleotide polymorphism (SNP)s in the FGF7 gene region and COPD susceptibility has been reported,^635^ although it is difficult to disentangle whether this is due to a direct role for FGF7 in COPD development or in lung development, predisposing to COPD later in life.

The biological roles of FGF7 (also known as KGF) and FGF10 in lung regeneration have been consistently reported to be supportive. Recombinant FGF7 and FGF10 promote survival and proliferation of epithelial cells in culture.^422^^,^^636^^,^^637^ FGF7 appears to be a stronger stimulus than FGF10 in this respect^422^; in fact, FGF7 signaling is required for alveolar epithelial cell organoids formation in fibroblast-free culture conditions.^638^ In vivo, FGF10 signaling is consistently reported to be required for both airway and alveolar epithelial repair. Thus, in the airways, FGF10 is expressed by bronchial smooth muscle and its conditional deletion impairs the epithelial repair response to naphthalene injury.^639^ In the alveolar region, lipofibroblasts express FGF10 in abundance^523^ and deletion or reduction in FGF10 expression is associated with hypomorphic lungs and impaired alveolar epithelial growth and differentiation during development.^640^ FGF7 plays key roles in lung development as well, particularly in branching morphogenesis^641^ and in alveolar epithelial cell survival and differentiation.^642^, ^643^, ^644^ Interestingly, and in addition to direct effects on epithelial repair, FGF signaling has also been reported to control elastin turnover by lung fibroblasts. Deficiency in FGFR3 and FGFR4 leads to aberrant lung development, characterized by enlarged airspaces and defective regulation of genes involved in elastogenesis. Isolated lung fibroblasts obtained from these mice did produce elastin, indicating that FGF signaling controls alveolar development and elastogenesis in utero by supporting mesenchymal–epithelial interactions that govern these responses.^645^ Because of these critical roles in both epithelial regeneration and matrix homeostasis, FGFs have been extensively studied as potential therapeutic agents for COPD.

##### a Fibroblast growth factor receptor 2

In spite of the subtle differences in FGF spatial regulation during homeostatic lung development and maintenance of lung tissue, exogenous administration of recombinant FGF proteins or modified versions hereof, tend to have quite similar and consistently protective effects in animal models. FGF2 deficiency fails to resolve the inflammation and epithelial repair response after either bleomycin or naphthalene injury.^646^ Furthermore, recombinant FGF2 protein, administered via intraperitoneal injection, reduced the inflammatory response and alveolar capillary leak in response to LPS exposure.^647^ Most relevant to the topic of this review, however, modified versions of FGF2, being either collagen-binding FGF2 or protein transduction domains conjugated FGF2, improved both airspace enlargement and inflammatory outcomes in mouse models of elastase-induced emphysema.^648^^,^^649^ Recombinant FGF2 also improves inflammatory outcomes in an animal model of CS exposure,^166^ and appears to be safe as an inhaled drug, although short-term (during a period of 2 weeks) administration in patients with stable COPD did not improve lung function or Borg scale outcomes.^166^ It will be of interest to evaluate the efficacy of this treatment in the context of COPD exacerbations as will be discussed in the section on clinical outcomes below.

##### b Fibroblast growth factor receptor 7/keratinocyte growth factor

Recombinant FGF7 (KGF) is one of the most extensively studied of these 3 FGF proteins, and is protective in vivo as well. KGF pretreatment improves lung repair outcomes in a mouse model of idiopathic pneumonia syndrome after bone marrow transplantation.^650^ KGF administration in vivo in rats also enhances the subsequent growth and repair response of AT2 cells in vitro indicating a direct relationship with epithelial cell activation.^651^ Similar protective effects were observed in a mouse model of oleic acid induced acute lung injury.^652^ When applied as a MSC-therapy approach that overexpresses KGF, the treatment protected against LPS-induced acute lung injury as well.^653^ Relevant to COPD, KGF administration in a preventive therapeutic regimen, attenuated the inflammatory response, and protected from elastase-induced emphysema development in a mouse model, but not when administered in a therapeutic regimen.^654^ This is at odds with a study that reported therapeutic effects of recombinant KGF in a mouse model of elastase exposure, in which effects on AT2 cell proliferation and the activation of p42/p44 MAPK signaling were also reported.^655^ The main differences between these 2 studies is the mode of administration, which was subcutaneous^654^ and via oropharyngeal instillation.^655^ On a speculative note, this may have impacted on the local bioavailability of KGF in the lung, hence explaining the better outcomes shown by Muyal et al.^655^ Interestingly, recombinant KGF is available as a clinically approved formulation (Palifermin) for chemotherapy-induced oral mucositis and has been clinically evaluated for safety and preliminary efficacy outcomes in human volunteers exposed to inhaled LPS. The results of this trial indicate that the preparation is safe, and that early beneficial effects on surfactant protein D and IL-Ra were observed.^656^ Moreover, BALF of volunteers treated with KGF, promoted alveolar epithelial repair and fibroblast proliferation in vitro.^656^ While these studies are promising, the clinical trial mentioned is over 10 years old already indicating no immediate additional follow-up was pursued. Clinicaltrials.gov does mention a trial in asthma, but without publication of results.

##### c Fibroblast growth factor receptor 10

Similar protective effects are reported for recombinant FGF10. Intratracheal application of FGF10 inhibits oxidative stress and ferroptosis in response to particulate matter in mouse lungs.^657^ This is associated with Gpx4 and Nrf2 pathway activation. A related study also reported protective effects on pyroptosis.^658^ In line with these findings, recombinant HGF protects against emphysema development in response to either CS or elastase in mice.^659^ Interestingly, in that study protective effects on pulmonary vascular changes of FGF10 were also observed, which is of interest in the context of altered EC function in emphysema. Further to this point, FGF10 restores the defective endothelial glycocalyx and prevents EC apoptosis in a mouse model of CS-induced COPD.^660^

#### 2 Hepatocyte growth factor

HGF is a secreted protein in the human lung that is among the best studied factors driving epithelial repair, both from a biological and from a pharmacological point of view. HGF signals via the receptor tyrosine kinase c-MNNG HOS transforming gene (MET) and plays critical roles in epithelial homeostasis and in lung cancer development. In that respect, any potential therapeutics targeting the HGF/c-MET pathway will have to balance the benefit of tissue repair with the risk of promoting lung cancer, in which HGF/c-MET inhibition is a desirable outcome.^661^ FGF10 is mainly expressed by lung fibroblasts and myofibroblasts as well as pericytes and pulmonary neuroendocrine cells.^662^ Its expression is also higher in LMSCs than in adipocyte or bone marrow–derived stromal cells.^164^ Its receptor c-MET on the other hand is abundantly expressed in most epithelial cell types and in the endothelium,^662^ in line with a role in lung mesenchymal to epithelial communication. Downstream of the c-MET receptor are the typical receptor tyrosine kinase-induced signaling pathways such as PI3K signaling, p42/p44 MAPK signaling, and FAK signaling.^663^

Consistent with a link between lung injury and repair, the expression of HGF increases in otherwise healthy patients with community acquired pneumonia, however, this increase is not observed in patients with acute exacerbations of COPD.^664^ Furthermore, patient with COPD-derived LMSCs have reduced expression of HGF mRNA,^164^ and lung fibroblasts obtained from patients with emphysema have reduced capacity for HGF production.^165^ Furthermore, cytokines relevant to COPD pathology such as TGF-*β* reduce HGF expression in lung fibroblasts, contributing to the negative effects of TGF-*β* on epithelial repair.^422^ Intriguingly, whereas 1 study reports reduced expression of HGF in the epithelial lining fluid of patients with COPD,^665^ other studies report no change or increased expression of HGF in BALF or in plasma.^666^^,^^667^ Thus, whereas the reduced capacity for mesenchymal HGF production in COPD is consistently reported, this is not necessarily reflected in other pulmonary compartments or in the systemic compartment. It will be of interest to investigate this apparent contradiction in further detail and to involve severity of COPD and the presence of emphysema versus airway disease in the analysis to understand this relationship more.

Consistent with a major role for HGF in emphysema development, genetic deletion of the c-MET receptor in mice is sufficient to induce airspace enlargement in mice.^668^ HGF based therapeutics have been evaluated both for emphysema and for other respiratory conditions in need for lung tissue repair such as acute lung injury and lung fibrosis. The first published report on the potential use of HGF in emphysema dates from 20 years ago already and used in vivo gene transfection of HGF in rat lungs as a proof-of-concept approach. In this study, an hemagglutinating virus of Japan (HVJ) packaged plasmid encoding for HGF was administered intravenously as a single dose, which provided sustained increases in HGF gene expression for 1 week in the rat lung tissues. This was accompanied by improvements in airspace enlargement associated with reduced apoptosis and improved proliferation of alveolar cells after elastase exposure.^669^ Interestingly, the treatment also restored pulmonary microvascular changes and functional changes on lung function.^669^ Similar beneficial effects of HGF were demonstrated for intranasal administration of recombinant HGF protein, which was effective even after 1 week of treatment duration already in an elastase model of emphysema in mice, without additional benefit from prolonged treatment for up to 4 weeks of duration.^670^ Cell therapy with human MSCs is also HGF dependent since both MSC therapy and treatment with conditioned media from these cells improved elastase-induced emphysema, whereas MSCs deficient in HGF were nearly ineffective.^671^ In vitro studies confirm that these effects are achieved by acting on alveolar epithelial cells, since HGF depletion inhibited the beneficial effects of bone marrow–derived stem cells on the growth and differentiation of AT2 cells,^672^ whereas direct administration of recombinant HGF boosts growth of alveolar epithelial cells grown in lung organoids.^422^ Antisense oligonucleotides against HGF also interfere with the growth and differentiation of fetal rat lung explants.^673^ On the other hand, alveolar organoids can form in the absence of HGF,^638^ indicating that its presence is supportive, but not strictly required.

#### 3 Bone morphogenetic proteins

BMPs are members of the TGF-*β* superfamily, and were initially discovered for their role in bone and cartilage formation.^674^, ^675^, ^676^ Besides their classical functions, BMPs have since emerged as pleiotropic factors involved in iron homeostasis, immune modulation, angiogenesis, stem cell regulation, and tissue repair. BMP ligands bind to type I receptors (activin receptor-like kinase 2 [ALK2 (ActR-I)], ALK3 [BMPR-IA], and ALK6 [BMPR-IB]), as well as type II receptors (activin receptor type IIA [ActR-IIA], ActR-IIB, and BMPR-II). This binding promotes phosphorylation of SMAD1/5/8 proteins. These combine with cytoplasmic SMAD4 as an active transcriptional complex and translocate to the nucleus, resulting in the activation of gene transcription.^674^

The best studied BMP ligands in the context of alveolar regeneration are BMP2, BMP4, and BMP6, which have the highest expression in whole lung tissue.^139^ The BMP signaling pathway plays a crucial role in AT2 cell growth and differentiation, though in ligand and context specific manners. For example, recombinant BMP4 appears to reduce alveolar epithelial organoid growth, whereas antagonists of BMP signaling such as follistatin and noggin promote AT2 self-renewal.^677^ In sharp contrast, cocultures of bronchioalveolar stem cells with ECs require BMP4 for alveolar lineage specification, indicating the context-dependent effect of BMPs.^135^ In line with this contention, BMP6 promotes alveolar epithelial cell growth,^139^ whereas BMP2 promotes AT2 cell differentiation into AT1 cells.^678^ Moreover, *Bmpr1a* ablation disrupted club cell regeneration in mice.^679^ However, recombinant BMP6 was unable to restore elastase-induced lung injury in precision-cut lung slices.^139^

BMP-Smad1/5/8 signaling is important for maintaining lung homeostasis and lung function. Interestingly, Smad1/5/8 signaling is downregulated in emphysema and mice that express the BMP antagonist Noggin selectively in AT2 cells develop emphysema spontaneously.^680^ In COPD, BMP6 expression is decreased in the lungs, an effect that is also observed in smokers and in mice exposed to CS.^681^ An association between BMP6 and lung function has been described in mice as well, leading to reduced total lung capacity and increased dynamic elasticity and tissue damping in *Bmp6−/−* mice.^681^ This is further supported by genome-wide association studies reporting associations between variants in the *BMP6* gene and forced vital capacity.^682^ Moreover, *BMP6* mRNA levels are downregulated during acute exacerbations compared with stable COPD.^683^ Similarly, BMP6 was upregulated in COPD rats treated with high-intensity electroacupuncture, which correlated with increased lung function and reduced inflammation.^684^

Mechanistically, *Bmp6*-deficient mice showed iron accumulation in multiple organs and loss of iron regulatory feedback mechanisms.^685^ Iron overload may further harm the surrounding tissues by promoting oxidative stress and cell death. Indeed, BMP6 is proangiogenic in both in vitro and in vivo.^686^^,^^687^ This angiogenic activity is important to COPD, as emphysema is associated with microvascular dysfunction and remodeling resulting from a reduction in capillary length and density.^136^ BMP6 works in both canonical and noncanonical pathways (SMAD-dependent and independent). Specifically, it triggers cell migration via p38-HSP27 signaling axis in tip cells, while inducing the activation of SMAD1/5 signaling in stalk cells.^686^ Consequently, increased migration in tip cells and proliferation in stalk cells occurs, leading to enhanced angiogenesis. BMP6 appears to be preferentially expressed by pulmonary microvascular ECs, but with functional effects on alveolar epithelial organoid growth as well, which is mechanistically explained by reduced oxidative stress signaling and enhanced WNT signaling.^139^ These findings highlight BMPs as a key regulator of alveolar and vascular repair, which is impaired in COPD. Given its dual role in epithelial regeneration and angiogenesis, further research into BMP-based therapies is warranted.

#### 4 Vascular endothelial growth factor

VEGF is a key regulator with important proangiogenic activity. Its role as a family of signaling proteins for vascular development and angiogenesis is well established.^688^ The VEGF family of growth factors consists of several subtypes, being VEGF-A, -B, -C, and -D, as well as placental growth factor. VEGF-A (often called “VEGF”) primarily functions as the main mediator of new blood vessel formation, whereas VEGF-C and -D are key regulators of lymphatic vessel formation. VEGF-B and placental growth factor, along with VEGF receptor-1 (VEGFR-1), have more restricted roles, with a less clear contribution to angiogenesis.^689^

VEGFs specifically interact with one or more type V receptor tyrosine kinases, VEGFR-1, -2, and -3 and with distinct coreceptors such as neuropilins and heparan sulfate proteoglycans.^690^ VEGFR1 plays a regulatory role by negatively modulating VEGFR2 activity and promoting the migration of monocytic cells. VEGFR2 serves as the driver of angiogenesis, orchestrating the differentiation of blood vascular EPCs, EC migration, proliferation, and survival. It also regulates sprouting angiogenesis, flow sensing, and vascular permeability.^691^ In contrast, VEGFR3 is predominantly involved in lymphangiogenesis, supporting the migration of lymphatic EPCs, lymphatic vessel expansion, and, to a lesser extent, contributing to blood vascular sprouting angiogenesis.^691^

Decreased VEGF levels have been reported in sputum from patients with emphysema compared with healthy individuals.^692^ In emphysema, reduced VEGF may contribute to or result from alveolar capillary loss and tissue destruction, whereas in chronic bronchitis, elevated VEGF may reflect ongoing angiogenesis, vascular remodeling in inflamed small airways.^693^^,^^694^ This indicates VEGF can be both beneficial and harmful. It may act as a protective feature to prevent emphysema while potentially detrimental by exacerbating inflammation in bronchial disease. Additionally, VEGF is emerging as a biomarker that might help to differentiate COPD phenotypes and possibly indicate underlying emphysema development.^695^, ^696^, ^697^

VEGF signaling may intersect with multiple pathogenic processes in COPD. Inhibition VEGF receptor signaling disrupts maintenance of alveolar structure, promotes oxidative stress and cell apoptosis, thereby contributing to pathogenesis of emphysema.^698^ Conversely, VEGF agonism supports cell survival by preventing increased oxidative stress, apoptosis, and modulates inflammation by affecting immune cell trafficking and survival.^693^^,^^699^ Both in silico and in vivo modeling showed that prominin-1-derived peptide, a novel short peptide that increases VEGF binding to ECs, prevents proteolytic degradation by enzymes such as elastase and plasmin, and reduced lung injury in 4- and 21-day elastase-induced murine emphysema models.^699^^,^^700^ Unlike direct treatment with VEGF protein, which could have off-target effects, stabilizing VEGF via prominin-1-derived peptide may be considered safer. Prominin-1-derived peptide is currently in the preclinical stage, but still represents a promising therapeutic avenue for emphysema treatment. Because no such treatment is currently available to clinically address COPD, recent studies provide a hopeful foundation suggesting that further investigation of the VEGF pathway as a therapeutic target may benefit patients in the future.

#### 5 Osteoglycin and its active fragments

Osteoglycin (OGN), also known as mimecan, has recently emerged as a promising candidate in regenerative pharmacology for COPD. Initially referred to as osteoinductive factor due to its role in bone formation, OGN was later found to be ubiquitously expressed. It is an endogenous small leucine-rich proteoglycan involved in various biological processes, including tissue development, ECM organization, and fibrosis regulation.^701^ The mature protein (∼37 kDa) comprises 7 tandem leucine-rich repeats and a C-terminal tail, and contains multiple glycosylation sites.^701^^,^^702^ Our recent proteomics-guided drug discovery approach identified OGN as a key factor secreted by lung fibroblasts that promotes alveolar epithelial repair.^703^ This aligns with the growing recognition of the pivotal role lung fibroblasts play in orchestrating epithelial regeneration within the alveolar niche.

Using murine and patient with COPD-derived lung epithelial organoids, OGN was shown to significantly increase the colony-forming efficiency of alveolar epithelial progenitors without affecting organoid size, suggesting a specific effect on progenitor cell activation rather than a broad proliferative response.^703^ Notably, OGN enhanced alveolar (surfactant protein C^+^) organoid differentiation even under injurious conditions such as CS extract exposure, indicating its potential to support epithelial maturation under COPD-relevant stressors.^703^ Interestingly, a smaller C-terminal fragment (∼15 kDa), comprising leucine-rich repeats 4 through 7 (MC002), was equally effective in supporting organoid formation and differentiation. Both OGN and MC002 also reduced elastase-induced lung injury in murine precision-cut lung slices, and high doses of MC002 significantly improved lung function parameters in an elastase-induced lung injury mouse model.^703^

Although initially identified for its role in bone formation, subsequent studies revealed that OGN is widely expressed across tissues, including the lung.^701^^,^^702^ In nondisease human lung samples, OGN expression was positively correlated with age.^704^ In contrast, OGN expression appears dysregulated in the lungs of individuals with COPD. Lin et al^705^ reported reduced OGN mRNA expression in lung biopsies of patients with severe COPD.^705^ Similarly, immunostaining for OGN on human lung tissue has shown proportionally lower expression in current smokers compared with nonsmokers, with ex-smokers displaying intermediate levels, suggesting that CS may exert a lasting suppressive effect on OGN expression. A trend toward lower OGN levels has also been observed in lung tissue from patients with severe early-onset COPD.^703^ These findings suggest that age-related upregulation of OGN in the lung may be disrupted by smoking, potentially contributing to impaired alveolar repair capacity. Given that reduced OGN levels are also present in otherwise healthy smokers, this dysregulation could represent an early molecular event that predisposes individuals to COPD, possibly requiring additional environmental or genetic insults to drive disease progression.

Although OGN expression appears to correlate with disease severity and smoking history, the mechanism of action of OGN and its active fragment is not fully understood yet. Transcriptomic analyses of OGN-treated lung epithelial cells revealed upregulation of protective epithelial pathways, including those involved in oxidative stress detoxification and iron homeostasis, processes increasingly implicated in COPD pathogenesis.^703^ Although these pathway-level changes provide initial mechanistic insight, the specific binding partners or downstream signaling cascades mediating OGN’s regenerative effects are currently unknown. However, emerging evidence suggests that OGN may modulate several key tissue repair pathways. For instance, studies in pulmonary fibrosis models demonstrated that OGN downregulation by microRNA-140 was associated with activation of the WNT/*β*-catenin signaling pathway.^706^ OGN has also been shown to modulate TGF-*β* signaling, a central pathway in alveolar remodeling. In cardiac fibrosis, OGN suppressed fibroblast proliferation and migration through inhibition of LPAR3/MMP2/EGFR signaling, reducing ECM deposition.^707^ In cancer, OGN overexpression inhibited epithelial-to-mesenchymal transition and reduced cell proliferation via downregulation of the PI3K/Akt/mTOR pathway.^708^ Beyond the lung, OGN also appears to play a role in systemic metabolic regulation, as knockout models revealed increased bone formation and altered insulin sensitivity.^709^

Taken together, these findings highlight OGN as a promising therapeutic candidate for COPD with demonstrated regenerative potential. However, further studies are required to elucidate its precise molecular binding partners and mechanisms of action, as well as to guide future clinical development.

#### 6 Other extracellular matrix-based strategies

In addition to OGN, several other matrix proteins or fragments hereof have been proposed as therapeutic options in preclinical models of emphysema. The protein hyaluronan and proteoglycan link protein 1 was shown to increase expression of sirtuins and reduce markers of cellular senescence in AT2 cells. Recombinant hyaluronan and proteoglycan link protein 1 protein also reduced emphysema development in an elastase-induced mouse model of emphysema.^710^

Similar protective effects were reported for keratan sulfate. In this study, a disaccharide repeating unit of keratan sulfate was shown to reduce emphysema development in response to elastase in the mouse, and to attenuate inflammation in both the elastase model and in an exacerbation model in which CS exposure is combined with LPS.^711^

## III Clinical outcomes and feasibility

In recent years, significant progress has been made in discovering therapeutic strategies aimed at tissue repair and regeneration in COPD. Numerous preclinical studies have demonstrated promising regenerative effects in experimental models, sparking optimism about the potential of these therapies to modify disease progression. However, despite scientific progress, none of these candidates have successfully advanced to clinical approval. The translation of regenerative therapies from bench to bedside remains challenging owing to the complexity of COPD and the difficulty of demonstrating disease modification in clinical trials. Here, we will discuss the clinical endpoints typically used to evaluate therapeutic efficacy in COPD trials, the feasibility of applying these endpoints to regenerative interventions, and how emerging biomarkers and alternative trial designs may help to overcome current translational barriers.

Safety issues may form a significant barrier to clinical introduction. Patients with COPD suffer from many comorbidities, among which lung cancer is most prominent in patients with a history of smoking, showing a 2- to 6-times higher risk compared with the general population.^712^ This should be kept in mind when evaluating the safety of regenerative therapies, since they intrinsically may further increase this risk. Cell-based therapies (MSC- or iPSC-based) carry a tumorigenic risk, therefore, these therapies should be evaluated already in early development stages on their tumorigenicity.^713^ On the other hand, stem cell-based therapies used in COPD were proven to be relatively safe so far.^714^^,^^715^ However long-term data are scarce.

Similar to cell-based therapies also EVs or exosomes may contain growth factors that may favor tumor progression. From this perspective, regenerative therapies that consist of only a single therapeutic compound should be preferred since the safety of such a single entity is much easier to establish than of the complex mixtures that occur in cells or cell-derived EVs or other mixtures.

### A Clinical endpoints

Although preclinical research into regenerative therapies for COPD has shown promising results, translating these findings into effective clinical treatments remains a major hurdle. According to the European Medicines Agency, demonstrating disease modification or slowing of disease progression requires long-term clinical trials that convincingly show a change in the trajectory of lung function decline.^716^^,^^717^ This is typically assessed through periodic measurements of FEV_1_, the volume of air a person can forcibly exhale in the first second.^718^ In clinical trials, the trough or prebronchodilator FEV_1_ is the most commonly used parameter.^719^ A minimal clinically important difference of 100 mL is generally accepted to represent a meaningful improvement.^258^^,^^718^, ^719^, ^720^ However, FEV_1_ alone is now considered insufficient to fully capture the therapeutic benefit of an intervention.^719^ When FEV_1_ is used as a primary endpoint, regulatory guidelines require it to be supported by a coprimary endpoint that reflects symptoms and patient-reported outcomes.^716^^,^^719^ Among the most frequently used additional clinical endpoints is the Transition Dyspnea Index, which evaluates changes in the severity of dyspnea across 3 domains: functional impairment, magnitude of task, and magnitude of effort. Each is scored from −3 to +3, resulting in a total score ranging from −9 to +9.^721^^,^^722^ A change of at least one point is considered clinically meaningful.^717^, ^718^, ^719^^,^^721^^,^^722^ Another widely accepted endpoint is the St. George’s Respiratory Questionnaire, a self-administered instrument that assesses health status across symptoms, activity limitations, and psychosocial impacts.^719^ Total scores range from 0 to 100, with a 4-point change considered the minimal clinically important difference.^717^, ^718^, ^719^^,^^721^^,^^722^ Additional outcomes commonly used in COPD trials include exacerbation frequency, exercise capacity (eg, 6-minute walk distance), rescue medication use, and imaging-based endpoints such as quantitative CT to assess emphysema progression or airway remodeling.^716^^,^^719^ Recent updates in regulatory and academic consensus now emphasize integrated and multidimensional endpoints, including composite measures such as clinically important deterioration or clinically important improvement, to better capture the complexity of COPD and the potential for disease modification.^722^ Moreover, the use of biomarkers, functional respiratory imaging, and digital monitoring tools is gaining importance in evaluating treatment response, particularly for precision-medicine approaches and regenerative interventions aiming to restore lung structure and function.^722^

To detect changes in disease trajectory, particularly in the context of regenerative therapies aimed at modifying disease progression, clinical trials require large patient cohorts and extended follow-up periods of at least 3 to 5 years.^723^^,^^724^ This duration is necessary to generate statistically and clinically meaningful data, particularly when assessing the slope of FEV_1_ decline over time between treatment groups. However, the long timelines, substantial costs, and logistical complexity of such trials present a significant barrier to the clinical translation of regenerative approaches in COPD. These challenges highlight the urgent need for alternative trial designs or early biomarkers that can serve as surrogates for long-term disease progression, thereby facilitating the development of disease-modifying therapies in this space.

To date, fibrinogen remains the only prognostic biomarker formally recognized by both the FDA and the European Medicines Agency for use in COPD drug development.^725^ As a blood clotting factor and acute-phase reactant, fibrinogen plays a key role in the body’s response to inflammation. In the context of COPD, the lung epithelium has been shown to produce fibrinogen in response to inflammatory stimuli. Elevated plasma fibrinogen levels (>3.5 g/L) have been associated with an increased risk of acute exacerbations and higher overall mortality among patients with COPD.^726^, ^727^, ^728^, ^729^, ^730^ As such, fibrinogen serves as an important biomarker for disease prognosis and identifying high-risk patient populations in clinical trials. In addition to fibrinogen, promising analytical techniques such as proteomics, metabolomics, single-cell and single-nucleus RNA sequencing, mass cytometry, and advanced imaging are increasingly being used to identify novel biomarkers in COPD.^731^, ^732^, ^733^, ^734^ For instance, soluble RAGE is under consideration for COPD to help identify subjects at risk of emphysema progression.^725^^,^^735^ Another proposed biomarkers is the peptide midrange proadrenomedullin as a predictor for mortality in COPD.^730^^,^^736^^,^^737^ Additionally, elevated levels of serum surfactant protein D, a multimeric glycoprotein involved in pulmonary innate immunity, correlated with progressive lung function decline and worsening of the health status of severe patients with COPD.^738^, ^739^, ^740^, ^741^ Many types of COPD biomarkers have been identified, including blood protein biomarkers, cellular markers, and protease enzymes, which have been collected from diverse biological sources such as peripheral blood, sputum, bronchoalveolar lavage fluid, exhaled air, and genetic material.^742^^,^^743^ Moreover, an emerging area of interest is the use of composite biomarker panels, which combine multiple markers to better predict disease severity, progression, and mortality.^744^, ^745^, ^746^ These multianalyte approaches may ultimately improve patient stratification and the development of personalized treatment strategies in COPD.^747^ Taken together, integrating biomarkers with traditional clinical measures could significantly enhance the monitoring of COPD progression during clinical trials, potentially reducing trial duration and enabling the rapid identification and discontinuation of ineffective compounds (“fast fail” strategies).

### B Disease heterogeneity

In addition to clinical readouts, the lack of progress in COPD drug development may largely be attributed to the heterogeneity of the disease. The traditional phenotypic distinctions of “pink puffers” and “blue bloaters” offer a simplistic view of the heterogeneous conditions encompassed by COPD. Studies have identified several phenotypic subpopulations within COPD, including eosinophilic, emphysema, and respiratory failure phenotypes.^748^ Furthermore, COPD exacerbations are categorized as eosinophil-driven, bacteria-driven, or frequent exacerbators.^748^ Patients with COPD can also be classified according to significant risk factors, including genetics, early-life events, infections, smoking and environmental tobacco smoke, and environmental exposure.^363^^,^^749^ Understanding the various trajectories and taxonomy of COPD, along with recent advances in COPD pathophysiology, has expanded its definition from a single disease to a syndrome with diverse manifestations and underlying mechanisms.^3^^,^^363^^,^^745^^,^^749^ To improve clinical trial outcomes, it is essential to address this complexity by designing trials that reflect the complexity of COPD. Advances in high-throughput methods offer new opportunities for personalizing treatments and drug discovery. By leveraging these methods, clinical trials can be designed to better match the diverse manifestations of COPD. For example, patient biopsies could be subjected to transcriptomic, proteomic, or metabolic profiling to identify specific molecular phenotypes.^750^^,^^751^ Single-cell sequencing of lung biopsies can reveal progenitor cell populations that regenerative therapies may target.^33^^,^^752^ Alternatively, creating lung organoids from patient-derived cells can serve as a platform for high-throughput compound screening, facilitating the discovery of personalized therapeutics.^32^

An emerging framework to address COPD heterogeneity is the treatable traits approach, which focuses on identifying and targeting specific, measurable characteristics that contribute to an individual patient’s disease burden, regardless of traditional diagnostic labels.^753^^,^^754^ These traits may include airway inflammation type (eosinophilic vs neutrophilic), bacterial colonization, exacerbation susceptibility, comorbidities, or impaired tissue repair capacity.^753^^,^^754^ By focusing on what mechanistically goes wrong in each patient, rather than the broad disease category, this approach enables more personalized interventions. However, applying a treatable traits framework to repair or regeneration therapy remains challenging, as the loss of lung function in COPD results from multifactorial processes, such as chronic inflammation, infection, protease imbalance, and cellular senescence. Nevertheless, defining traits related to regenerative potential, such as progenitor cell exhaustion or matrix remodeling capacity, could help identify subgroups most likely to benefit from regenerative or reparative interventions in future clinical trials.^755^ This approach will ensure that clinical trials are tailored to these well defined patient cohorts, improving the likelihood of translating preclinical successes into clinical practice. By adopting these strategies and embracing a broader, more nuanced understanding of COPD, clinical trials can more effectively address the complexity of the disease and enhance therapeutic outcomes.^749^^,^^756^

### C The exacerbation period as a key window of opportunity

**Fig. 7:**
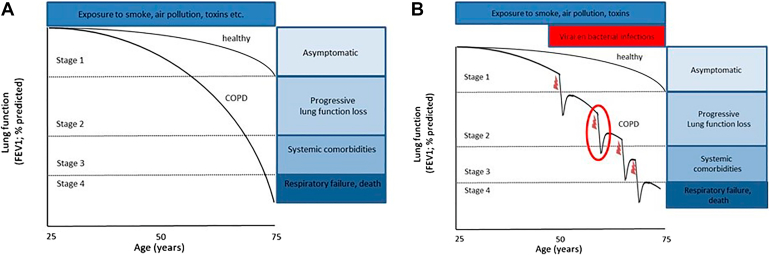
Progressive lung function loss in patients with COPD throughout life. (A) Exposure to smoke, air pollution, and toxins can cause lung injury, which—if not adequately repaired—contribute to loss of lung function, which is progressive and results in systemic comorbidities, and eventually respiratory failure and death. (B) Revised view on lung function loss in COPD. At present, lung function loss in COPD is no longer viewed as a gradual decline as depicted in panel (A), but as an intermittent process driven by episodes of disease worsening (exacerbations). These episodes are often associated with bacterial and viral infections and represent opportunities for targeted pharmacological treatment.

Although patient heterogeneity is a major barrier to therapeutic success in COPD, another challenge lies in trial design, specifically the long timelines required to demonstrate clinical efficacy. An alternative approach may lie in reconsidering the timing of therapeutic intervention. Instead of focusing solely on long-term decline, the exacerbation period may represent a critical and underutilized window of opportunity for the prevention of excessive lung function decline. These exacerbations are a hallmark of COPD, particularly in patients with more advanced disease and many of which are triggered by viral and/or bacterial infections.^757^^,^^758^ Prior history of exacerbations, older age, the presence of comorbidities, COPD severity and the presence of eosinophilic inflammation are the most significant predictors for the occurrence of exacerbations, underscoring their multifactorial nature.^759^ Clinically, an exacerbation is defined as a worsening of the patient’s baseline dyspnea, cough, and/or sputum that is acute in onset and necessitates a change in regular medication.^14^ Beyond their immediate impact on symptoms and quality of life, exacerbations are increasingly recognized as key drivers of disease progression. It is a relatively new understanding that progressive lung function decline in COPD is not linear, but rather episodic in nature as a result of incomplete recovery from loss of function during an exacerbation (Fig. 7). In fact, exacerbations are estimated to account for the majority of the accelerated lung function loss throughout the life of a patient with COPD.^760^ Therefore, treatments aimed at reducing exacerbation risk may have an important preventive effect in the management of lung function decline.

In the context of regenerative pharmacology, the exacerbation period offers an additional and compelling therapeutic opportunity. In the healthy lung, bacterial and viral infections trigger robust activation of alveolar epithelial progenitors to facilitate repair after injury.^761^^,^^762^ Although this repair response is also observed in COPD, it is often incomplete, leading to a net decline in lung function after each exacerbation (Fig. 7).^760^ Thus, the exacerbation period is characterized by both heightened disease burden during the exacerbation phase and the concurrent activation of endogenous repair pathways in the period following an exacerbation. This makes the exacerbation window itself an opportunity for therapeutic intervention, particularly for therapies that antagonize the detrimental effects of the inflammatory microenvironment on lung repair, while the recovery phase after an exacerbation may be suitable for therapeutics that directly promote repair. Targeting the relatively well defined time window of (the aftermath of) an exacerbation offers several advantages. Lung function recovery postexacerbation can be assessed over a matter of weeks, rather than years, and within a relatively controlled clinical setting using the patient as their own control. This could substantially reduce the duration and complexity of clinical trials for regenerative therapies. Moreover, this temporally confined setting may allow for the use of therapeutics that are unsuitable for long-term maintenance, thereby reducing cumulative risk exposure. Taken together, the exacerbation period may represent a biologically and clinically optimal window to evaluate and deliver regenerative interventions aimed at restoring lung function in COPD.

### D Route of administration and formulation

Drugs and advanced therapy medicinal products under development for regenerative lung therapies in COPD do not differ from any other medicinal product in the sense that their route of administration and formulation are mainly determined by the combination of their physicochemical properties, their structure, their site of action and their intended therapeutic objectives. The formulation scientist must evaluate and balance these 4 interrelated technical and biopharmaceutical factors, each presenting specific opportunities and constraints, when making decisions during the design and development of the dosage form. What may make the formulation and administration of products for lung regeneration special is that so far, the described therapies, cover the full range of possible drug substance options varying form small organic molecules, therapeutic proteins, nanosized vesicle-like structures to advanced cell therapies as summarized in the sections above.^32^^,^^749^ In addition, inhalation of the medicine offers a unique option for targeted drug administration to the lungs, which may substantially increase the therapeutic effect that can be obtained with certain therapies through increased exposure of the lungs to the drug.^763^^,^^764^ However, at the same time this route of administration is not suitable for all medicines, since mucosal and epithelial barriers may prevent the drug from reaching the target, especially when this target is not at the luminal side of the epithelium.

Oral administration is most convenient for the patient; however, its application for administering drugs for lung regeneration is limited to those drugs that have sufficient oral bioavailability. The oral route is especially suitable for drugs that show a tendency to accumulate in the lungs to concentrations surpassing blood levels, irrespective of the route of administration. The antituberculosis drug bedaquiline is an example of such a drug.^765^

In contrast to oral administration the inhalation route, applying liquid or solid aerosols, offers a more targeted approach for therapies acting locally in the lungs. This route of administration enables high drug concentrations at the site of action while avoiding hepatic first-pass metabolism. As such inhalation is an attractive option for a wide range of drug substances, from small molecules to large biopharmaceuticals. Basically, an inhaled drug has to overcome 2 barriers before it may exert any therapeutic activity. First, penetration and deposition of the aerosol into the airways is required. This, physical barrier can be overcome by generating aerosols with a size range between 1 and 5 *μ*m. Larger particles will not sufficiently penetrate the airways, whereas particle in the nanometer range will not show deposition and merely be exhaled again. Secondly, the drug must reach its site of action, which may often require the passage of the epithelial barrier of the airways or alveoli. The airway and alveolar epithelium are highly permeable to orally inhaled small molecules, which allows these molecules to reach also targets beyond the luminal side of the lung epithelium. Over the past decade a several excellent reviews and books have been published on the development and use of inhalation systems and the formulations used for small molecules and will therefore not be further discussed here.^766^^,^^767^

Many of the drugs and advanced therapy medicinal products currently under investigation for regenerative therapies in COPD are biopharmaceuticals.^749^ In addition, sparked by the development of inhaled insulin,^766^ there is today a plethora of information on the formulation and administration of peptide- and protein-based drugs. In general, the protein’s instability is a major issue in formulating them. Approaches to tackle their instability includes the application of stabilizing excipients such as, buffers, (poly)saccharides, polyols, surfactants and specific salts and drying of the formulation by lyophilization or spray-drying.^768^, ^769^, ^770^ Next to the formulation, the inhalation device is relevant to the success of the therapy. Because dried formulations produced through lyophilization or spray-drying are more stable than liquid formulation, dry powder inhalers may be more suitable for the administration of proteins than the liquid-based nebulizers. Furthermore, ultrasonic nebulizers may affect the structural integrity of the protein.^771^^,^^772^ Proteins were among the first biopharmaceuticals explored for the treatment of COPD. Whether the pulmonary route is suitable for a protein is determined by the protein’s molecular weight (size) and the location of the target. The molecular mass of proteins that exert their action in the lumen of the airway or alveoli, is not relevant. However, for proteins with a site of action beyond the epithelial lining of the lungs, the molecular weight is important. Proteins with a molecular mass over 1.0 to 1.3 kDa do not pass the airway epithelium, whereas the proteins with a molecular mass over 22 kDa are not absorbed via the alveolar lining. Higher molecular weight proteins may therefore be unable to reach their site of action after inhalation.^771^^,^^773^

Currently, the field of biopharmaceutics has developed beyond peptides and proteins, and today also includes nucleic acid-based therapies (mRNA, small interfering RNA, and antisense oligonucleotides) and EVs, similar to proteins, these therapies may also be suitable for pulmonary administration. Nucleic acid-based therapies are often formulated into LNPs. These particles protect the genetic material from enzymatic breakdown and enhance the cellular uptake by endocytosis or pinocytosis. LNPs consist of ionizable cationic lipids, pegylated lipids, phospholipids and cholesterol, and microfluidic technologies have been used to encapsulate the oligonucleotide (eg, mRNA) in these particles.^774^ There is evidence that after inhalation, LNPs can transfect lung cells.^775^^,^^776^ Recently, it was demonstrated that pulmonary endothelial-targeted LNPs were capable to deliver mRNA to enhance vascular repair.^777^ It is important to realize that LNPs are too large to pass the airway epithelium. Instead, they are internalized by cells located in the superficial layer of the airway epithelium, where the cargo delivered by the LNPs can subsequently exert its therapeutic effect. As an alternative to inhalation, intravenous administration of self-assembling, one-component ionizable Janus dendrimer-based LNPs has been proposed for lung-targeted gene delivery, demonstrating effective mRNA delivery and potential for lung regeneration.^778^

As summarized in section Extracellular vesicles, EVs have recently emerged as a potential therapeutic for regenerative treatments.^594^^,^^598^^,^^749^ EVs are characterized by their poor stability, which makes them unsuitable as regular therapeutics, certainly when they are dispersed in a liquid where they require storage at −80 °C. However, recently it has been found that EVs incorporated in an inulin matrix in the glassy state by freeze drying, stabilized the fragile vesicle structure even at temperatures up to 20 °C for 12 weeks at 43% relative humidity, while maintaining the biophysical properties and regenerative capacity. A similar spray-dried powder formulation, which next to the inulin also contained 4% leucine was suitable for inhalation via a dry powder inhaler.^616^ Recently, various techniques to produce powders for inhalation containing different biopharmaceuticals, including EVs were reviewed.^779^

Finally, bone marrow mononuclear cells such as mesenchymal BM-MSCs or iPSCs are used for regenerative therapies.^32^^,^^714^^,^^749^ Since autologous stem cell therapies are fully personalized therapies, the sourcing, isolation growing and formulation of the cells can only be done in the hospital or in highly specialized nearby the hospital. Cells are usually kept in culture media such as Dulbecco’s modified Eagle’s medium. For storage the cells can be frozen in liquid nitrogen when the Dulbecco’s modified Eagle’s medium is supplemented with 10% dimethyl sulfoxide. For infusion the cells are generally formulated in saline or PBS, which may be supplemented with human serum albumin. Cells are unsuitable for administration via the inhalation route. Having sizes significantly over 5 *μ*m (15–20 *μ*m) implies that upon inhalation lung deposition would not reach beyond the first 2 bifurcations and most of the cells would end up in the throat. When the size of the cells would be reduced to <5 to 7 the cellular structure would be destroyed, and the cells would lose their functionality.

## IV Conclusions

The field of regenerative medicine in COPD is advancing rapidly, propelled by new insights into epithelial progenitor biology, inflammatory signaling, and the molecular pathways that govern alveolar repair. This review has highlighted how COPD represents not merely a disease of progressive tissue destruction, but a failure of endogenous repair systems, a concept that reshapes both our understanding of pathogenesis and our therapeutic ambitions.

The alveolar epithelial niche is shaped by a dynamic interplay between epithelial cells, immune cells, fibroblasts, ECs, and the ECM. Key signaling pathways such as WNT/*β*-catenin, FGF, BMP, and HGF have emerged as central regulators of epithelial proliferation, differentiation, and survival. However, these pathways are frequently disrupted in COPD through inflammatory cytokines, cellular senescence, oxidative stress, and altered mesenchymal-epithelial crosstalk. Strategies that restore balance in these signaling networks, whether through direct activation, suppression of antagonistic signals, or modulation of the niche environment, have shown encouraging results in preclinical models.

At the same time, this review has underscored the multifaceted challenges that remain. COPD is a disease with heterogeneous endotypes and stages, and regenerative approaches must contend not only with damaged epithelium but also with persistent inflammation, matrix remodeling, vascular dysfunction, and cellular senescence. As such, future therapies are unlikely to succeed through single-target strategies. Instead, multimodal interventions, combining regenerative, anti-inflammatory, and senolytic components, may be needed to overcome the entrenched pathological milieu.

The preclinical advances summarized here provide a robust foundation, but translation to patients will require rigorous clinical validation, optimized delivery systems (eg, inhaled biologics and vesicle-mediated delivery), and improved patient stratification tools. Biomarkers that predict regenerative potential or senescence burden could enhance trial design and treatment outcomes. Moreover, the timing of intervention, early versus late-stage disease, will likely determine therapeutic success. In this context, exacerbations may offer a clinically actionable window of opportunity: a transient phase of epithelial injury and heightened niche activity that could be leveraged to support regeneration. Designing clinical trials around these episodes may therefore improve both biological efficacy and measurable outcomes.

In conclusion, the field stands at a pivotal moment. A growing arsenal of regenerative candidates, ranging from small molecules and cell therapies to EV-loaded ligands and cell-derived proteins, is entering a phase of translational readiness. The next decade may witness a paradigm shift in COPD care: from symptom management and damage control, to interventions that restore lung architecture and function.

## Conflict of interest

Luke van der Koog and Henderik W. Frijlink are employees and stock owners of MimeCure.
